# A Dual-Stream Feature Learning Framework with Adaptive Bio-Inspired Protozoa–Starfish Optimization for Melanoma Classification

**DOI:** 10.3390/biomimetics11100701

**Published:** 2026-10-02

**Authors:** Sreelakshmi Jayasankar, Thomas Brindha

**Affiliations:** 1 Department of Computer Science and Engineering, Noorul Islam Centre for Higher Education, Kanyakumari 629180, India; 2 Department of Information Technology, Noorul Islam Centre for Higher Education, Kanyakumari 629180, India; brindha@niuniv.com

**Keywords:** Cross-Scale Adaptive Semantic Fusion, dermoscopic images, lesion boundary attention, MaxViT-Tiny, melanoma, Res2Net-50

## Abstract

Melanoma accounts for a small fraction of skin cancer cases but for the majority of skin-cancer-related deaths, making accurate early detection essential. Although deep learning-based dermoscopic image analysis has shown strong diagnostic potential, its performance depends strongly on training hyperparameters and loss-function weighting, which are commonly tuned manually or through computationally expensive grid search. To address this, Adaptive Combined Artificial Protozoa–Starfish Optimization (AC-APSO) is introduced as a bio-inspired hyperparameter optimization strategy that dynamically regulates the contributions of the Artificial Protozoa Optimizer (APO) and the Starfish Optimization Algorithm (SFOA) at each iteration according to population diversity, fitness improvement, and optimization progress. AC-APSO configures RM-LBAT-Net, a hybrid CNN–Transformer architecture that integrates Res2Net-50-based multi-scale feature extraction, MaxViT-Tiny-based global contextual learning, a Deformable Lesion Boundary Attention Module (DLBAM), and Cross-Scale Adaptive Semantic Fusion (CSASF). The optimized framework achieved an accuracy of 98.85%, with precision, recall, and F1-score of 98.70%, 99.00%, and 98.85%, respectively. Controlled ablation studies showed that each architectural component contributed under both fixed and optimized configurations and that adaptive coordination outperformed APO alone, SFOA alone, and a fixed-weight APO–SFOA combination. In a head-to-head comparison under an identical protocol, the framework outperformed contemporary architectures, including ConvNeXtV2-Tiny and Swin Transformer V2-Tiny. Five-fold cross-validation and external validation on the ISIC 2016 dataset (97.36% accuracy) further indicated stable performance and promising cross-dataset generalization.

## 1. Introduction

The skin represents the body’s most extensive organ system and serves as a critical defensive interface, shielding the body from environmental stressors, pathogenic invasion, mechanical injury, and ultraviolet (UV) exposure [1]. Structurally, it is organized into three distinct strata—the epidermis, dermis, and hypodermis—which collectively support physiological functions and protect underlying tissues [2]. Melanocytes located in the epidermis produce melanin, which helps protect cellular DNA from UV-induced damage. However, prolonged UV exposure, genetic predisposition, and environmental factors can disrupt normal cellular processes and facilitate the onset of skin cancer.

Among cutaneous cancers, melanoma poses the greatest clinical threat, largely due to its rapid progression and high metastatic potential [3,4]. Although melanoma accounts for a comparatively small percentage of diagnosed skin malignancies, it drives a majority of associated fatalities, largely due to its propensity to metastasize to remote organs. Recent global estimates indicate more than 325,000 new melanoma cases and approximately 57,000 deaths annually [5]. Early detection is therefore essential for improving patient outcomes, as prognosis decreases considerably after progression to advanced or metastatic stages. Clinically, melanoma is commonly assessed using characteristics such as asymmetry, irregular borders, color heterogeneity, and lesion diameter, as reflected in the ABCD diagnostic criteria [6]. However, distinguishing melanoma from benign skin lesions remains challenging because of their considerable visual similarity, particularly during the early stages of disease.

Histopathological examination following biopsy remains the diagnostic gold standard for melanoma [7]. Nevertheless, biopsy is invasive, time-consuming, and costly. Dermoscopy provides a non-invasive imaging modality that enhances visualization of subsurface skin structures and can support more accurate lesion assessment [8]. Despite these advantages, dermoscopic interpretation remains dependent on clinical expertise and experience, which can introduce diagnostic variability. These constraints have consequently motivated the development of computer-aided diagnostic (CAD) methods capable of providing accurate, consistent, and efficient melanoma classification.

Early CAD approaches predominantly relied on conventional machine learning methods using handcrafted color, texture, and shape descriptors. Although such approaches provided useful initial results, their effectiveness was constrained by limited feature representation and generalization capability. With the rapid growth of artificial intelligence and machine learning across biomedical research, convolutional neural networks (CNNs) have emerged as a preferred approach for melanoma classification, given their inherent capability to extract hierarchical features from dermoscopic images without manual intervention [9,10,11,12]. However, conventional CNN architectures may lose fine-grained spatial information through repeated downsampling and pooling operations, limiting their ability to represent irregular lesion boundaries, heterogeneous pigmentation patterns, and lesion-wide structural relationships.

To address these constraints, this study develops a hybrid deep learning architecture termed RM-LBAT-Net (Res2Net-50-MaxViT Lesion Boundary-Aware Transformer Network) for automated melanoma detection using dermoscopic images. The architecture employs a dual-stream feature extraction framework consisting of a Res2Net-50 branch for hierarchical multi-scale local representation learning and a MaxViT-Tiny (MaxViT-T) branch for global contextual dependency modeling. Res2Net-50 captures discriminative local characteristics such as micro-textural variations, pigmentation heterogeneity, asymmetric structures, and border discontinuities, whereas MaxViT-Tiny combines convolutional operations with local window and global grid attention to establish lesion-wide contextual relationships. To further improve sensitivity to clinically relevant lesion structures, the Deformable Lesion Boundary Attention Module (DLBAM) incorporates edge-aware processing and deformable spatial attention to emphasize irregular and asymmetric lesion boundaries. The extracted representations are subsequently integrated using the Cross-Scale Adaptive Semantic Fusion (CSASF) strategy, which generates input-dependent fusion coefficients to dynamically combine local texture, global contextual, and boundary-enhanced semantic information according to the characteristics of each lesion. The fused representation is further refined through residual enhancement and channel recalibration before classification using Global Average Pooling (GAP) and fully connected (FC) layers. A hybrid objective combining Focal Loss and Categorical Cross-Entropy (CCE) Loss is employed to support robust discrimination between benign and malignant lesions.

In addition to architectural design, the selection of appropriate training hyperparameters is important for obtaining stable convergence and effective generalization. Learning rate, batch size, dropout rate, weight decay, and the relative weighting of the hybrid-loss components can substantially influence the training behavior and classification performance of RM-LBAT-Net. Manual or empirical selection of these parameters may not consistently provide an effective configuration for a complex hybrid architecture. Therefore, an automated hyperparameter optimization mechanism is incorporated to determine a suitable training configuration while keeping the established network architecture unchanged.

To address this requirement, an Adaptive Combined Artificial Protozoa–Starfish Optimization (AC-APSO) strategy is proposed as a bio-inspired hyperparameter optimization mechanism for RM-LBAT-Net. The proposed strategy combines the complementary search characteristics of Artificial Protozoa Optimization (APO) and Starfish Optimization Algorithm (SFOA), while introducing an adaptive mechanism to regulate their relative contributions according to population diversity, fitness improvement, and the current optimization state. Unlike a fixed or sequential combination of optimization algorithms, AC-APSO dynamically balances the contributions of the two bio-inspired search mechanisms throughout the optimization process. The approach is employed to optimize the training and hybrid-loss-related hyperparameters of RM-LBAT-Net while preserving the established network architecture. Consequently, the final classification system consists of an AC-APSO-optimized RM-LBAT-Net framework capable of jointly exploiting multi-scale local features, global contextual information, lesion-boundary characteristics, and an automatically optimized training configuration. The primary contributions of this research are:

- A novel RM-LBAT-Net architecture is developed by integrating Res2Net-50-based multi-scale local feature extraction and MaxViT-Tiny-based global contextual learning to capture complementary fine-grained and lesion-wide representations from dermoscopic images.
- A Deformable Lesion Boundary Attention Module (DLBAM) is proposed to enhance lesion-sensitive feature learning using Sobel gradient extraction, Laplacian edge enhancement, learnable edge convolutions, and deformable spatial attention for improved representation of irregular and asymmetric lesion boundaries.
- A Cross-Scale Adaptive Semantic Fusion (CSASF) strategy is introduced to dynamically integrate local texture, global contextual, and boundary-sensitive semantic representations using input-dependent fusion coefficients generated from the joint feature representation, thereby allowing the relative contribution of each feature stream to vary across lesion samples.
- An Adaptive Combined Artificial Protozoa–Starfish Optimization (AC-APSO) strategy is proposed as a bio-inspired hyperparameter optimization mechanism that adaptively combines Artificial Protozoa Optimization and Starfish Optimization to determine effective training and hybrid-loss configurations for RM-LBAT-Net.
- A comprehensive experimental evaluation is conducted using optimization ablation, architectural ablation, five-fold cross-validation, external dataset validation, comparative analysis, and Grad-CAM visualization.

The remainder of this paper is organized as follows: Section 2 provides a review of relevant literature. Section 3 describes the proposed RM-LBAT-Net architecture and the AC-APSO-based hyperparameter optimization strategy. Section 4 presents the results. Section 5 offers concluding remarks and suggests avenues for future work.

## 2. Related Works

Skin cancer classification has progressively evolved from traditional machine learning techniques to advanced deep learning and transformer-based frameworks, driven by the need for accurate and early diagnosis. Initially, conventional machine learning approaches relied on handcrafted features extracted from dermoscopic images. Although these approaches demonstrated moderate performance, they were highly dependent on feature engineering and failed to generalize effectively across datasets due to variations in lesion appearance and imaging conditions.

Alrabai et al. [13] employed transfer learning using VGG16, DenseNet169, and DenseNet201 on a Kaggle dermoscopic dataset containing 3297 images, where DenseNet201 attained an accuracy of 87.88% and AUC of 0.9532 due to enhanced feature propagation; however, the study was limited by dataset homogeneity and lack of computational efficiency analysis. Similarly, Nguyen et al. [14] utilized CNN models such as ResNet50, InceptionV3, and EfficientNet on ISIC Archive and HAM10000 datasets, achieving 92.5% accuracy, 91.8% sensitivity, and 93.1% specificity, but the model suffered from sensitivity to image variability and lack of clinical metadata integration. Mahmud et al. [15] developed an Xception-based model using two melanoma datasets (2597 and 2081 images), achieving accuracies of 95.23% and 96.48%, respectively, though limitations included dataset diversity issues and computational complexity.

To further enhance classification performance, hybrid models combining deep learning with optimization and feature engineering techniques were introduced. Mittal et al. [16] introduced a framework integrating optimization algorithms with a convolutional deep spiking neural network on the ISIC 2017 dataset, achieving up to 95.07% accuracy, but the model relied heavily on handcrafted feature engineering and lacked interpretability. Saleh et al. [17] combined CNN architectures such as AlexNet and ResNet with Grey Wolf Optimization and machine learning classifiers on ISIC 2017 data, achieving 94.5% accuracy; however, the approach exhibited high computational complexity and limited generalizability. Claret et al. [18] developed a wavelet-based CNN model using the HAM10000 dataset (10,015 images), achieving 94% sensitivity and 91% specificity, but increased computational cost and reduced interpretability were noted due to complex feature transformations.

Furqan et al. [19] proposed a Vision Transformer-based multimodal framework integrating dermoscopic images with clinical metadata using the HAM10000 dataset (10,015 images), achieving 93.4% accuracy, though the model required structured metadata and high computational resources. Prasanna et al. [20] introduced STViTDA-Net using the ISIC2019 dataset (25,331 images), incorporating GAN-based augmentation and deformable attention, which outperformed baseline models but suffered from high training cost and potential bias from synthetic data. Huang et al. [21] developed LSATrans using clinically annotated datasets, achieving AUC up to 0.9801, though it required accurate lesion localization and involved high computational overhead. Ellis et al. [22] evaluated pathology foundation models on 2130 whole-slide images, achieving AUROC of 0.925, but limitations included dataset bias and reliance on advanced digital pathology infrastructure. Chaurasia et al. [23] proposed a self-supervised Vision Transformer using 1.13 million histopathological patches, achieving strong agreement (k = 0.898), yet faced challenges such as class imbalance and limited melanoma representation.

Chiu et al. [24] implemented a two-stage ensemble combining EfficientNet, Vision Transformer, and Swin Transformer on ISIC datasets (19,163 images), achieving 96.9% accuracy and reduced false negatives, but the model exhibited high computational complexity and limited generalizability due to smaller clinical datasets. Khan et al. [25] proposed a transfer learning framework with ROI extraction and PCA-based feature reduction on HAM10000, achieving approximately 87% accuracy, though the approach was computationally intensive and dependent on preprocessing. Saha et al. [26] developed a federated learning-based CNN framework on ISBI2016 data, achieving 92.08% accuracy while preserving data privacy, but faced challenges related to communication overhead and heterogeneous data bias. Hamim et al. [27] introduced the SmartSkin-XAI model using DenseNet121 on ISIC 2020 and Kaggle datasets (~10,000 images), achieving 97% accuracy, though limitations included sensitivity to demographic variations and reliance on post hoc explainability.

More recently, Aksoy et al. [28] evaluated Vision Transformer, Swin Transformer, and ConvNeXt models on a Kaggle dermoscopic dataset comprising 13,900 images, where ConvNeXt achieved the highest accuracy of 91.5%; however, image acquisition conditions, model explainability, and prediction uncertainty were not examined. Mateen et al. [29] proposed a hybrid framework integrating U-Net-based lesion segmentation, Inception-ResNet-v2 feature extraction, and Vision Transformer-based classification on the ISIC 2020 dataset (33,126 images), achieving 98.65% accuracy, 99.20% sensitivity, and 98.03% specificity, although the multi-stage pipeline depended on a separate segmentation stage and increased computational cost. Metaheuristic optimization has also been applied to the configuration of CNN-based melanoma classifiers. Hermosilla et al. [30] compared CNN models optimized using Cuckoo Search, the Firefly Algorithm, the Whale Optimization Algorithm, Particle Swarm Optimization, the Grey Wolf Optimizer, and the Crow Search Algorithm on a balanced subset of HAM10000 (10,410 images), achieving a best accuracy of 91.25%; however, the optimized models were restricted to shallow CNN backbones. Nasiri et al. [31] optimized a custom CNN using a hybrid Grey Wolf Optimization–Particle Swarm Optimization strategy on 10,000 dermoscopic images, achieving 95.3% accuracy, 94.8% precision, 96.5% recall, and an AUC of 0.98, although no external validation was reported. Sajid et al. [32] introduced a morphology-guided attention network incorporating explainability and uncertainty estimation for seven-class skin lesion classification on HAM10000, achieving 99.12% accuracy, yet the evaluation was limited to a single dataset.

### Research Gap

However, existing methods for melanoma detection still exhibit several limitations that restrict their ability to achieve robust and accurate classification. First, CNN-based methods tend to emphasize local feature extraction and are prone to losing fine-grained spatial detail as a result of repeated downsampling operations, limiting their ability to characterize subtle lesion structures. Second, variations in lesion appearance, imaging conditions, and class distributions across dermoscopic datasets can adversely affect the generalization capability of existing approaches, and several recent studies have been evaluated on a single dataset without independent external validation [30,31,32]. Third, irregular lesion boundaries, which represent important morphological characteristics of melanoma, are not always adequately captured by conventional feature extraction mechanisms. Such approaches may emphasize dominant regional appearance while providing limited representation of contour fragmentation, boundary asymmetry, and abrupt pigmentation transitions. Fourth, many hybrid and ensemble approaches rely on direct concatenation or static feature fusion, which may not sufficiently regulate the relative contribution of local, global, and boundary-sensitive representations according to the characteristics of individual lesions. In addition to these representation-level limitations, the effectiveness of deep learning-based melanoma classification is influenced by the selection of appropriate training and loss-related hyperparameters. Parameters such as dropout rate, learning rate, batch size, weight decay, and the relative weighting of complementary loss functions can affect convergence, generalization, and class-wise discrimination. Empirical selection of these parameters may not consistently provide an effective configuration for complex hybrid architectures integrating convolutional and transformer-based representations. Although metaheuristic algorithms have been applied to configure melanoma classifiers [17,30,31], existing approaches typically employ either a single optimizer or a fixed combination of optimizers, in which the relative contribution of each search mechanism remains constant throughout the optimization process [31]. Moreover, such approaches have largely been applied to relatively shallow CNN architectures [30,31] rather than to hybrid convolutional–transformer models. Therefore, an adaptive optimization mechanism capable of systematically exploring the hyperparameter search space, and of dynamically regulating the contributions of complementary search strategies according to the optimization state, is desirable for improving the training configuration of melanoma classification models. This motivates the development of a unified framework that combines multi-scale local representation, global contextual modeling, lesion-boundary-aware feature refinement, adaptive semantic fusion, and bio-inspired hyperparameter optimization within a single melanoma classification framework.

## 3. Materials and Methods

The proposed system for automated melanoma lesion classification from dermoscopic images begins with dataset partitioning into development and independent test subsets to ensure unbiased evaluation, as shown in Figure 1. The preprocessing stage consists of pixel-intensity normalization, hair artifact removal using DullRazor, and Contrast Limited Adaptive Histogram Equalization (CLAHE) to obtain standardized and enhanced image representations. Online data augmentation is subsequently performed solely on the training set. The resulting preprocessed images are provided to the dual-stream hybrid RM-LBAT-Net architecture, which integrates Res2Net-50 and MaxViT-Tiny (MaxViT-T) branches for complementary local and global representation learning. The Res2Net-50 branch performs hierarchical multi-scale feature extraction to capture discriminative local lesion characteristics, while the MaxViT-Tiny branch combines MBConv embedding with local window and global grid attention to model contextual dependencies and lesion-wide structural relationships. To further enhance lesion-sensitive representation learning, the DLBAM is incorporated to emphasize irregular lesion contours and boundary variations through edge-aware deformable attention refinement. Subsequently, the CSASF strategy generates input-dependent fusion coefficients from the joint feature representation to adaptively integrate local texture representations, global contextual embeddings, and boundary-enhanced semantic information. Unlike globally shared fusion weights, the resulting coefficients vary according to the feature characteristics of each input lesion, enabling dynamic weighting of the three representation streams. The fused representation undergoes residual refinement and channel recalibration using global contextual weighting before classification. Finally, GAP and FC layers are employed to perform benign and malignant melanoma classification. To optimize the training configuration of RM-LBAT-Net, an AC-APSO strategy is incorporated as a bio-inspired hyperparameter optimization mechanism. The optimized hyperparameter configuration is subsequently fixed and used for final training of RM-LBAT-Net, while Adam remains responsible for gradient-based optimization of the trainable network parameters. Thus, AC-APSO operates at the outer hyperparameter-optimization level, whereas Adam performs the inner gradient-based optimization of the network weights. The effectiveness of the resulting AC-APSO-optimized RM-LBAT-Net framework is evaluated using multiple performance metrics, component-wise and optimization-wise ablation analyses, five-fold cross-validation, external dataset validation, baseline-model comparison, state-of-the-art comparison, and visualization-based interpretation using Grad-CAM.

### 3.1. Dataset

The melanoma dermoscopic images used in this study were acquired from a publicly available Kaggle repository [33]. According to the repository description, the images were compiled from diverse sources; however, the specific original clinical or archival sources, acquisition devices, and imaging protocols are not documented. The dataset version used in this study consists of 13,879 dermoscopic images categorized into two lesion categories: benign and malignant, provided as separate train and test directories comprising 11,879 images (6289 benign, 5590 malignant) and 2000 images (1000 benign, 1000 malignant), respectively. The images are provided in a standardized spatial resolution of 224 × 224 pixels, ensuring compatibility with deep learning-based classification frameworks. The dataset is provided at the image level only: the images are distributed with sequential numeric file names and without accompanying metadata, such as patient- or lesion-level identifiers or original archive image identifiers, and the procedure used to assign images to the training and test directories is not described by the repository. Accordingly, the predefined training and test partition was adopted as provided, consistent with its intended use. The dataset captures substantial diversity in lesion pigmentation, texture distribution, boundary irregularity, asymmetry, lighting conditions, and morphological characteristics of lesions, which play a key role in classification of melanoma. Representative images from the dataset and the corresponding class distribution are depicted in Figure 2 and Figure 3, which reveal a reasonably balanced overall distribution of 7289 benign and 6590 malignant cases.

### 3.2. Data Splitting

The dataset was provided with a predefined train–test partition. The training directory, containing 11,879 images, was used for model development, while the test directory contained 2000 images. To examine potential duplication between the two partitions, all 11,879 training images and 2000 test images were compared using SHA-256 hashes of the image files and hashes of the decoded pixel arrays. No exact file duplicates and no pixel-identical images were identified between the training and test sets. However, because lesion-level identifiers are unavailable, the possibility of near-duplicate images, such as re-encoded or slightly transformed copies, or of different images of the same lesion or patient across the two partitions could not be completely ruled out. Within the training directory, the images were further divided using a stratified 85:15 split into 10,098 training images, comprising 5346 benign and 4752 malignant lesions, and 1781 validation images, comprising 943 benign and 838 malignant lesions. As patient- and lesion-level identifiers were not available, this training–validation split was performed at the image level. The validation subset was kept separate from the training subset and used to monitor model convergence, evaluate candidate configurations during AC-APSO-based hyperparameter optimization, and support selection of the final training configuration. No class-balancing or resampling techniques, including oversampling or undersampling, were applied. The original class distribution was retained throughout model development, while class imbalance was addressed through the class-balancing factor incorporated into the Focal Loss formulation. The final optimized configuration was subsequently trained using the designated training data and evaluated on the 2000-image test set.

### 3.3. Preprocessing

Prior to model training, a systematic preprocessing pipeline was employed to improve image quality, enhance lesion visibility, and ensure consistent feature representation for the proposed framework. Initially, pixel intensities were normalized to the [0, 1] range to standardize the input intensity distribution and stabilize gradient propagation during backpropagation, as expressed:

(1) $$I_{\mathrm{norm}}=\frac{I}{255}$$

where *I* is the original pixel intensity and *I_norm_* is the normalized pixel value. Subsequently, image enhancement operations were performed to improve diagnostically significant lesion structures. A DullRazor-based hair removal process using a 17 × 17 rectangular morphological structuring element and morphological black-hat filtering was employed to identify and remove hair artifacts, occlusions, and undesired noise that could interfere with lesion characterization. Detected hair regions were reconstructed using Telea inpainting with an inpainting radius of 1 pixel. Furthermore, CLAHE was used to enhance local lesion contrast, pigmentation visibility, low-intensity dermoscopic regions, and border clarity while avoiding excessive noise amplification using adaptive histogram clipping. CLAHE was performed on the luminance component of the CIELAB representation, with the contrast enhancement controlled by a clip limit of 2.0 and an 8 × 8 tile arrangement. This setting improves localized contrast in the images while preventing excessive enhancement in areas with relatively uniform intensity. These preprocessing methods enhance texture discrimination, lesion boundary visibility, and feature consistency, allowing for more effective lesion-sensitive representation learning within the deep learning framework. Figure 4 shows the successive preprocessing stages, including the original images, hair-removed outputs, CLAHE-enhanced images, and final preprocessed dermoscopic samples.

### 3.4. Augmentation

Model robustness was enhanced through online data augmentation, performed exclusively on the training data during model training. Instead of pre-generating and storing additional augmented images as a separate dataset, random transformations were dynamically applied to each training batch, allowing different augmented variants of the original images to be presented to the network across training epochs. The online augmentation pipeline incorporated both geometric and photometric transformations, including random rotation within ±30°, horizontal flipping, vertical flipping, random zoom within ±20%, shearing within ±0.2, brightness adjustment, and elastic transformation. Random rotation was sampled within the range of −30° to +30°, while random zoom was applied within a ±20% range. Shearing was performed within the range of −0.2 to +0.2. Horizontal and vertical flipping were used to expose the model to changes in lesion orientation. Brightness adjustment was applied to introduce variations in illumination, while elastic transformation was used to introduce controlled morphological deformation. The augmentation operations were applied stochastically during training, such that augmented samples could differ across training epochs. These transformations improve the robustness of the RM-LBAT-Net framework to variations in lesion orientation, scale, illumination, and morphology while preserving diagnostically relevant lesion characteristics. No augmentation was applied to the validation or test samples, thereby ensuring that model evaluation was performed using unmodified images. Figure 5 illustrates representative examples of the transformations generated through the online augmentation pipeline.

### 3.5. Model Development

The developed architecture incorporates a dual-stream learning approach that combines a Res2Net-50 branch for multi-scale local feature extraction with a MaxViT-Tiny (MaxViT-T) branch for global contextual representation learning. To further strengthen lesion-sensitive feature discrimination, the Deformable Lesion Boundary Attention Module (DLBAM) and Cross-Scale Adaptive Semantic Fusion (CSASF) mechanisms are incorporated for boundary-aware feature refinement and input-dependent semantic fusion, respectively. Together, these components enable the network to exploit complementary local, global, and lesion-boundary information for improved melanoma classification performance and generalization.

#### 3.5.1. Dual-Stream Feature Extraction

Following preprocessing and augmentation, the dermoscopic images are fed into the developed RM-LBAT-Net model. Melanoma lesions exhibit substantial variability in pigmentation distribution, texture heterogeneity, lesion asymmetry, border irregularity, and structural morphology. Therefore, a dual-stream feature extraction strategy is employed to simultaneously capture fine-grained local lesion characteristics and global contextual dependencies. As in Figure 6, the feature extraction stage consists of two parallel architectures: a Res2Net-50 branch and a MaxViT-T branch. The input dermoscopic image is denoted as:

*I* ∈ ℝ*^H^*^×*W*×*C*^ (2)

where *H*, *W*, and *C* are the parameters of image height, width, and channel respectively. Initially, a shallow convolutional embedding is performed to generate low-level spatial representations, shared as the common input to both branches:

*F*_0_ = *σ*(*Conv*(*I*)) (3)

where *Conv*(.) denotes convolutional transformation and *σ* represents nonlinear activation.

The generated feature representation *F*_0_ is simultaneously forwarded to the Res2Net-50 branch, which performs hierarchical multi-scale convolutional learning for discriminative local feature extraction. Res2Net-50 improves multi-scale representation capability by dividing feature channels into multiple subsets and processing them through residual hierarchical connections within a single residual block [34]. This mechanism enables simultaneous learning of fine and coarse lesion structures at multiple receptive fields. The Res2Net-50 branch progressively extracts clinically relevant dermoscopic characteristics including pigmentation irregularities, micro-textural variations, asymmetric lesion structures, border discontinuities, and fine morphological abnormalities.

The Res2Net-50 branch adopts the standard 26w4s configuration, with a base width of 26 and a scale factor of 4, distributed across four residual stages containing 3, 4, 6, and 3 blocks, respectively. The input feature map is divided into multiple scale-wise feature groups, as:

*F*_0_ = [*x*_1_, *x*_2_,…,*x_s_*] (4)

where *s* represents the number of scale groups. Hierarchical residual learning is then performed sequentially across scales, following a piecewise formulation in which the first group is passed through directly, the second group is convolved independently, and each subsequent group incorporates the output of the preceding scale:

(5) $$y_{i}=\left\{\begin{array}{cc} x_{i}, & i=1\\ \mathrm{Con}v_{i}\left(x_{i}\right), & i=2\\ \mathrm{Con}v_{i}\left(x_{i}+y_{i-1}\right), & 2<i\leq s \end{array}\right.$$

where *y_i_* denotes the output feature at scale *i*, *Conv_i_* represents the corresponding 3 × 3 convolution operation for that scale, and *y_i_*_−1_ corresponds to residual information propagated from the previous scale. The final Res2Net-50 output features are extracted using multi-scale feature fusion:

*F_Res_*_2*Net*_ = *Concat*(*y*_1_,*y*_2_,…,*y_s_*) (6)

This hierarchical residual learning strategy improves sensitivity toward subtle lesion abnormalities and enhances local multi-scale representation learning.

Simultaneously, the same feature representation *F*_0_ is forwarded to the MaxViT-T branch, which is a hybrid convolution–transformer architecture designed for efficient global contextual dependency modeling. MaxViT combines convolutional operations with local window attention and global grid attention mechanisms to record both proximal and distal semantic interactions within dermoscopic lesions [35]. This enables effective representation of lesion-wide structural patterns while maintaining computational efficiency. Initially, the input feature map is processed through a Mobile Inverted Bottleneck Convolution (MBConv) block to encode neighborhood-level spatial patterns and local lesion structures, as expressed:

*F_c_* = *MBConv*(*F*_0_) (7)

where *F*_0_ denotes the shallow feature representation obtained during the first phase of embedding.

Subsequently, local window self-attention is employed to model contextual interactions among neighboring lesion regions. The self-attention operation is formulated as:

(8) $$\mathrm{Attention}(Q,K,V)=\mathrm{Softmax}\left(\frac{QK^{T}}{\sqrt{d_{k}}}\right)V$$

where *Q*, *K*, and *V* signify query, key, and value matrices and *d_k_* is the attention scaling factor.

The resulting local contextual representation is:

*F_lw_* = *Attention*(*W_Q_F_c_*, *W_K_F_c_*, *W_V_F_c_*) (9)

where *W_Q_*, *W_K_*, and *W_V_* represent learnable projection matrices. This local-window attention mechanism captures fine-grained contextual relationships within localized lesion regions and improves representation of pigmentation variations, texture heterogeneity, and small structural abnormalities.

To further capture long-range dependencies and global semantic interactions, MaxViT applies grid attention across spatially distant regions of the lesion image, as expressed:

*F_grid_* = *Attention_grid_*(*F_lw_*) (10)

The grid attention mechanism enables information exchange between distant spatial locations, thereby improving representation of lesion asymmetry, pigmentation spread, border irregularities, and large-scale melanoma patterns.

Finally, the local and global contextual representations are aggregated to generate the final MaxViT-T feature embedding as:

*F_MaxViT_* = *Concat*(*F_lw_*, *F_grid_*) (11)

where *Concat*(.) denotes feature concatenation. The resulting representation simultaneously preserves localized semantic information and global contextual dependencies, enabling comprehensive characterization of complex melanoma lesions.

Before subsequent integration with the boundary-aware pathway, the Res2Net-50 and MaxViT-Tiny feature embeddings, *F_Res_*_2*Net*_ and *F_MaxViT_* are transformed into a common spatial and channel dimension using learnable 1 × 1 convolutional projection layers. Specifically, the projected representations are expressed as $F_{\mathrm{local}}^{p}$ and $F_{\mathrm{global}}^{p}$, respectively, where both representations share the same spatial resolution and channel dimension. This alignment enables their subsequent feature integration without dimensional incompatibility and provides a common semantic space for DLBAM processing. The exact feature-extraction stages, spatial resolutions, channel dimensions, and projection dimensions are reported in the implementation details subsection to ensure reproducibility. The aligned feature representations are subsequently forwarded to the DLBAM for explicit lesion-boundary enhancement and deformable spatial refinement.

#### 3.5.2. Deformable Lesion Boundary Attention Module (DLBAM) for Boundary-Aware Feature Refinement

Following dual-stream feature extraction, the representations generated by the Res2Net-50 and MaxViT-T branches are processed using the DLBAM to enhance lesion-boundary representation and improve sensitivity toward clinically significant contour abnormalities. Melanoma lesions commonly exhibit irregular borders, fragmented contours, asymmetric edge structures, and abrupt pigmentation transitions. However, conventional convolutional operations generally focus on dominant regional textures and often fail to explicitly emphasize diagnostically important lesion boundaries. Therefore, the developed DLBAM is designed to strengthen edge-sensitive representation learning through a unified lesion-boundary refinement pathway that integrates explicit gradient extraction, Laplacian edge enhancement, learnable edge feature transformation, and geometry-aware deformable spatial attention [36]. Initially, the integrated feature representation is expressed as in:

(12) $$F_{\mathrm{in}}=F_{\mathrm{local}}^{p}+F_{\mathrm{global}}^{p}$$

As shown in Figure 7, the first stage of DLBAM performs edge extraction on *F_in_* to capture lesion-sensitive contours and boundary discontinuities. Horizontal and vertical edge gradients are obtained using channel-wise (depthwise) Sobel filtering, where each channel of *F_in_* is convolved independently with the same 2D Sobel kernels, as in Equations (13) and (14):

*G_x_* = *F_in_* ∗ *S_x_* (13)

*G_y_* = *F_in_* ∗ *S_y_* (14)

where *S_x_* and *S_y_* denote horizontal and vertical Sobel kernels, respectively. The overall edge magnitude representation is subsequently obtained as:

(15) $$G=\sqrt{G_{x}^{2}+G_{y}^{2}}$$

The explicit extraction of directional gradients provides direct sensitivity to local intensity transitions occurring around lesion contours. This is particularly relevant for lesions with fragmented, asymmetric, or poorly defined boundaries, where boundary information may otherwise be diluted during conventional feature extraction.

To further emphasize abrupt lesion-boundary transitions and irregular contour structures, Laplacian enhancement is additionally incorporated, as:

*E_L_* = ∇^2^*F_in_* (16)

where ∇^2^ denotes the Laplacian operator. The extracted Sobel and Laplacian representations are concatenated and forwarded through learnable edge convolution operations to generate boundary-sensitive semantic features, as:

*F_edge_* = *Conv*([*G*, *E_L_*]) (17)

where [*G*, *E_L_*] represents feature concatenation. This operation improves representation of irregular lesion contours, asymmetric edge structures, and fragmented boundary regions associated with melanoma progression.

The methodological role of this stage is not to treat the Sobel and Laplacian responses as final attention maps, but to transform the explicitly extracted first- and second-order boundary cues into learnable semantic features. Thus, DLBAM combines complementary boundary information with trainable feature transformation before performing spatial refinement. This design allows the module to preserve explicit contour evidence while learning task-relevant boundary representations from the dermoscopic feature space.

Subsequently, the generated edge-sensitive features are processed using deformable spatial attention mechanisms. Unlike conventional fixed-grid convolution, deformable convolution dynamically adapts receptive fields according to lesion geometry and contour variability. The deformable convolution operation is mathematically represented as:

(18) $$F_{\mathrm{def}}(p)=\sum_{k=1}^{K}w_{k}\cdot F_{\mathrm{edge}}(p+p_{k}+\Delta p_{k})$$

where *p* denotes the spatial location, *p_k_* represents regular convolution sampling coordinates, Δ*p_k_* corresponds to learnable spatial offsets, and *w_k_* denotes convolutional weights. This adaptive sampling mechanism enables effective modeling of lesion-boundary deformation, contour irregularity, and spatial morphological variability.

The combination of explicit boundary extraction and deformable sampling provides two complementary forms of boundary modeling. The Sobel and Laplacian operations provide direct sensitivity to boundary transitions and local contour changes, whereas deformable convolution allows the spatial sampling pattern to adapt to nonuniform lesion geometry. Therefore, DLBAM does not rely solely on handcrafted edge responses or deformable convolution independently; instead, these operations are integrated into a sequential refinement pathway in which explicit boundary evidence is transformed into semantic features and subsequently refined using geometry-aware spatial sampling.

The deformable feature representation is subsequently transformed into a lesion-boundary attention map through sigmoid activation, as:

*A_boundary_* = *σ*(*Conv*(*F_def_*)) (19)

where *A_boundary_* denotes the boundary attention representation and *σ* represents sigmoid activation. Finally, lesion-sensitive feature refinement is performed by applying the generated attention representation to the extracted semantic features, as:

*F_DLBAM_* = *F_in_* ⊗ *A_boundary_* (20)

where ⊗ denotes element-wise multiplication. This boundary-aware refinement operation enhances the representation of diagnostically relevant lesion contours and provides boundary-sensitive semantic features for subsequent adaptive semantic fusion. Overall, the methodological contribution of DLBAM lies in integrating explicit first-order gradient cues, second-order Laplacian enhancement, learnable boundary feature transformation, and deformable spatial attention within a unified lesion-boundary refinement pathway. Rather than introducing these operations individually as novel components, DLBAM combines them to address complementary aspects of dermoscopic lesion boundaries, including boundary visibility, abrupt contour transitions, semantic boundary representation, and spatial morphological variability.

#### 3.5.3. Cross-Scale Adaptive Semantic Fusion (CSASF) and Classification Module

Following boundary-aware refinement through the DLBAM, the generated feature representations are forwarded to the Cross-Scale Adaptive Semantic Fusion and Classification module for adaptive integration of complementary semantic information and final melanoma prediction, as illustrated in Figure 8. Melanoma lesions exhibit substantial variability in pigmentation distribution, lesion morphology, structural asymmetry, texture characteristics, and boundary irregularities. Consequently, relying on a single feature representation may limit classification performance. Therefore, the CSASF module is designed to effectively combine local multi-scale features, global contextual representations, and boundary-aware semantic information into a unified discriminative embedding [37].

Initially, the local representations extracted by the Res2Net-50 branch, the global contextual features generated by the MaxViT branch, and the boundary-aware representations produced by the DLBAM are transformed into a common semantic space through channel projection operations. This alignment process ensures dimensional consistency and improves feature compatibility before fusion.

To enable input-dependent fusion, the aligned local, global, and boundary-aware representations are initially concatenated along the channel dimension to construct a joint semantic representation:

*F_C_* = [*F*_local_ ‖ *F*_global_ ‖ *F*_boundary_] (21)

where ‖ denotes feature concatenation. A compact descriptor of the current input lesion is then obtained using Global Average Pooling (GAP), as:

*z* = *GAP*(*F_C_*) (22)

where *z* represents the input-specific semantic descriptor obtained from the combined local, global, and boundary-aware representations. The descriptor is subsequently processed by a lightweight gating network to generate the fusion coefficients. The hidden representation is expressed as:

*h_g_* = *δ*(*W*_1_*z* + *b*_1_) (23)

where *W*_1_ and *b*_1_ are learnable weight and bias parameters, respectively, and *δ*(⋅) denotes the ReLU activation function. The hidden representation is then transformed into three fusion logits as:

*q* = *W*_2_*h_g_* + *b*_2_ (24)

where *W*_2_ and *b*_2_ are learnable parameters and *q* = [*q_l_*, *q_g_*, *q_B_*] denotes the logits corresponding to the local, global, and boundary-aware representations, respectively. The logits are normalized using the Softmax function to generate input-dependent fusion coefficients:

[*α*(*x*), *β*(*x*), *γ*(*x*)] = *Softmax*(*q*) (25)

where *α*(*x*), *β*(*x*), and *γ*(*x*) denote the input-dependent fusion coefficients for the local, global, and boundary-aware representations, respectively. The coefficients satisfy:

*α*(*x*) + *β*(*x*) + *γ*(*x*) = 1 (26)

Subsequently, the adaptive fusion of the three semantic representations is performed as:

*F*_fusion_ = *α*(*x*)*F*_local_ + *β*(*x*)*F*_global_ + *γ*(*x*)*F*_boundary_ (27)

Unlike globally shared learnable coefficients, these weights are generated from the feature representation of each input lesion. Consequently, the relative contribution of the three feature streams can vary across lesion samples according to their encoded semantic characteristics.

The generated fused representation preserves complementary lesion information across multiple semantic levels while reducing redundancy between feature streams. This input-conditioned integration simultaneously exploits local texture patterns, lesion-wide contextual relationships, and diagnostically significant boundary characteristics without imposing an identical fusion ratio on every lesion.

To further enhance semantic discrimination, residual feature refinement is employed as:

*F_ref_* = *F_fusion_* + *Conv*(*F_fusion_*) (28)

This operation strengthens feature propagation and improves representation continuity. Subsequently, channel recalibration is performed to emphasize informative semantic channels and suppress less relevant activations, as expressed:

*F_cal_* = *F_ref_* ⊗ *σ*(*W_c_*(*GAP*(*F_ref_*))) (29)

where *W_c_* is learnable channel weights. Through input-dependent fusion, residual refinement, and channel-wise recalibration, the framework generates a compact semantic representation suitable for melanoma characterization. Following channel recalibration, the refined feature maps are spatially aggregated into a succinct feature vector while preserving the most informative lesion characteristics, as:

*F_gap_* = *GAP*(*F_cal_*) (30)

The pooled feature vector is subsequently processed through FC layers to learn high-level discriminative embeddings required for melanoma classification, as:

*F_fc_* = *δ*(*W_f_F_gap_* + *b_f_*) (31)

where *W_f_* and *b_f_* are trainable weight and bias parameters, respectively.

Finally, Softmax activation is employed to compute the probability of each lesion category as:

(32) $$P(y_{i})=\frac{e^{z_{i}}}{\sum_{j=1}^{C}e^{z_{j}}}$$

where *P*(*y_i_*) is the probability of class *i*, *z_i_* is the predicted logit value, and *C* is the total output classes.

Each lesion is assigned to the class exhibiting the highest predicted probability, thereby producing the final benign or malignant classification. Through the integration of input-dependent adaptive feature fusion, residual semantic refinement, channel recalibration, and classification, RM-LBAT-Net constructs a discriminative representation by exploiting complementary local, global, and boundary-aware lesion features. Although the architectural configuration remains fixed, its classification performance depends on the selection of appropriate training and loss-related hyperparameters. Therefore, the developed RM-LBAT-Net is subsequently subjected to an adaptive bio-inspired hyperparameter optimization process using the proposed AC-APSO strategy.

#### 3.5.4. Adaptive Combined Artificial Protozoa–Starfish Optimization

The performance of RM-LBAT-Net is influenced not only by its architectural design but also by the selection of suitable training hyperparameters. In particular, the dropout rate, learning rate, batch size, weight-decay coefficient, and relative contribution of the hybrid loss components directly affect convergence, regularization, and classification performance. Empirical selection of these parameters may not provide an effective configuration for the proposed hybrid architecture. Therefore, a bio-inspired hyperparameter optimization strategy is introduced to automatically determine an effective training configuration for RM-LBAT-Net.

The proposed method, AC-APSO, combines the complementary search behaviors of the APO and SFOA. APO is inspired by protozoan survival behavior and employs autotrophic and heterotrophic foraging, dormancy, and reproduction mechanisms to support exploration and population diversification [38]. SFOA is inspired by the behavioral characteristics of starfish and incorporates exploration, preying, and regeneration mechanisms to provide global search and refinement of promising solutions [39]. The original search mechanisms of APO and SFOA are retained, while an adaptive coordination mechanism is introduced to regulate their relative contributions during optimization. The internal parameters of the original algorithms are retained as follows: in APO, the number of neighbor pairs *n_p_*, which determines how many neighboring solutions contribute to the foraging update, is set to 2, and the maximum proportion fraction *p_f_*_,max_, which limits the fraction of the population undergoing dormancy or reproduction in each iteration, is set to 0.10; in SFOA, the switching parameter *G_p_*, which determines the probability of selecting the exploration or exploitation phase, is set to 0.50.

The principal novelty of AC-APSO lies in the adaptive regulation of the relative contribution of APO and SFOA throughout the optimization process. Unlike a fixed combination or sequential application of the two optimization algorithms, the proposed strategy dynamically adjusts their relative influence according to population diversity, fitness improvement, and optimization stage. Greater APO influence is provided when the population becomes concentrated or optimization progress stagnates, thereby promoting search diversification. As promising hyperparameter regions become established, the relative influence of SFOA increases to support focused refinement.

Each candidate solution represents one possible training configuration of RM-LBAT-Net and is expressed as:

(33) $$X_{i}^{t}=\left[\eta_{i}^{t},B_{i}^{t},d_{i}^{t},\lambda_{i}^{t},\alpha_{i}^{t}\right]$$

where *i* denotes the candidate index, *t* represents the current optimization iteration, *η* denotes the learning rate, *B* denotes the batch size, *d* denotes the dropout rate, *λ* denotes the weight-decay coefficient, and $\alpha_{i}^{t}$ denotes the Focal Loss weighting coefficient.

The corresponding Categorical Cross-Entropy coefficient is defined as:

(34) $$\beta_{i}^{t}=1-\alpha_{i}^{t}$$

where *β* represents the Categorical Cross-Entropy weighting coefficient. Thus, only the Focal Loss coefficient is directly optimized, while the CCE coefficient is derived to maintain a normalized combination of the two loss components. Accordingly, the two loss coefficients satisfy $\alpha_{i}^{t}+\beta_{i}^{t}=1$.

The search ranges considered for the optimized hyperparameters are presented in Table 1. The architectural parameters of RM-LBAT-Net, including the Res2Net-50 scale configuration, residual-block configuration, MaxViT attention heads, and attention-window size, remain fixed throughout the optimization process and are therefore excluded from the AC-APSO search space.

The initial candidate population is generated within the predefined hyperparameter search space as:

(35) $$X_{i,j}^{0}=L_{j}+r_{i,j}\left(U_{j}-L_{j}\right)$$

where $X_{i,j}^{0}$ denotes the initial value of the *j*-th hyperparameter for candidate *i*, *L_j_* and *U_j_* represent the corresponding lower and upper bounds, respectively, and *r_i_*_,*j*_ is a uniformly distributed random number in the interval [0, 1].

Subsequently, every candidate configuration undergoes training on the training subset, followed by performance evaluation on the validation subset. To account for overall classification performance and balanced class-wise discrimination, validation accuracy, macro-F1 score, and AUC are jointly considered. The fitness function is as:

*F_i_* = *w_A_*(1 − *A_i_*) + *w_F_*(1 − *F*1*_i_*) + *w_C_*(1 − *AUC_i_*) (36)

where *A_i_*, *F*1*_i_*, and *AUC_i_* denote the validation accuracy, macro-F1 score, and AUC of candidate *i*, respectively. The coefficients w_A_, w_F_, and w_C_ represent their corresponding weighting factors and satisfy:

*w_A_* + *w_F_* + *w_C_* = 1 (37)

In this study, equal weighting is assigned to the three validation criteria, such that *w_A_* = *w_F_* = *w_C_* = 1/3. This provides balanced consideration of overall accuracy, class-balanced F1 performance, and threshold-independent discrimination. The complementary terms (1 − *A_i_*), (1 − *F*1*_i_*), and (1 − *AUC_i_*) convert the maximization-oriented metrics into a minimization objective. Therefore, a lower fitness value indicates a superior hyperparameter configuration.

The best candidate at iteration *t* is obtained as:

(38) $$X_{\mathrm{best},t}=X_{i^{*},t},\;\;\;\;\;\;i^{*}\;=\underset{i}{\arg\min}\;F_{i}$$

where *i*^∗^ denotes the index of the candidate with the minimum fitness value, and *X_best_*_,*t*_ is the corresponding candidate hyperparameter configuration at iteration *t*.

The APO component retains the original protozoan-inspired foraging, dormancy, and reproduction mechanisms. These mechanisms provide complementary search behavior and population diversification within the hyperparameter space. Consistent with the original APO and SFOA formulations, each candidate’s update also incorporates the global-best solution from the prior iteration to guide the search. The APO-generated candidate is represented generally as:

(39) $$X_{i,t}^{\mathrm{APO}}=A_{\mathrm{APO}}\left(X_{i,t},\;X_{\mathrm{best},t-1}\right)$$

where 𝒜*_APO_* denotes the original APO search operations and *X_best_*_,*t*−1_ denotes the global-best hyperparameter configuration obtained at the previous iteration. The APO component contributes alternative search directions and population diversification, particularly when candidate solutions become concentrated around limited regions of the search space.

The SFOA component retains the original exploration, preying, and regeneration mechanisms. These operations provide complementary global exploration and local refinement of promising hyperparameter configurations. The SFOA-generated candidate is:

(40) $$X_{i,t}^{\mathrm{SFOA}}=A_{\mathrm{SFOA}}\left(X_{i,t},\;X_{\mathrm{best},t-1}\right)$$

where 𝒜*_SFOA_* represents the original SFOA search operations, and *X_best_*_,*t*−1_ ensures that both APO- and SFOA-generated candidates are guided consistently by the same, already-available global-best solution at the start of iteration *t*.

The principal methodological contribution of AC-APSO is the adaptive coordination of the two bio-inspired optimization mechanisms. A fixed contribution of APO and SFOA may not provide an appropriate exploration–exploitation balance throughout the complete optimization process. Therefore, the proposed framework estimates the current population diversity and fitness improvement before determining the relative contribution of the two mechanisms.

The normalized population diversity is calculated as:

(41) $$D_{t}=\frac{1}{N}\sum_{i=1}^{N}\frac{{∥X_{i,t}-{\bar{X}}_{t}∥}_{2}}{{∥U-L∥}_{2}+\epsilon }$$

where *D_t_* denotes the normalized population diversity, *N* represents the population size, *L* = [*L*_1_, *L*_2_,…, *L_m_*] and *U* = [*U*_1_, *U*_2_,…, *U_m_*] denote the lower- and upper-bound vectors of the *m* optimized hyperparameters, ${\bar{X}}_{t}$ represents the population mean at iteration *t*, ‖⋅‖_2_ represents the Euclidean norm, and *ϵ* is a small positive constant.

The relative improvement in the best fitness is calculated as:

(42) $$I_{t}=\left\{\begin{array}{cc} 1, & t=1\\ \frac{|F_{\mathrm{best},t-1}-F_{\mathrm{best},t-2}|}{|F_{\mathrm{best},t-2}|+\epsilon }, & t>1 \end{array}\right.$$

where *I_t_* represents the relative fitness improvement based only on the two preceding iterations’ global-best fitness values, and *F_best_*_,*t*−1_, *F_best_*_,*t*−2_ denote the best fitness obtained at iterations *t* − 1 and *t* − 2, respectively. For the first iteration (*t* = 1), *I*_1_ is initialized to 1 in the absence of a prior iteration. A smaller value of *I_t_* indicates limited recent improvement in the optimization process.

The adaptive APO contribution is then determined by:

(43) $$w_{\mathrm{APO},t}=\mathrm{clip}\left[c_{1}\left(1-D_{t}\right)+c_{2}\left(1-I_{t}\right)+c_{3}\left(1-\frac{t}{T}\right),0,1\right]$$

where *w_APO_*_,*t*_ denotes the APO contribution, *c*_1_,*c*_2_,*c*_3_ are non-negative control coefficients, *t* is the current iteration, *T* is the maximum number of iterations, and clip(⋅) restricts the coefficient to the interval [0, 1]. The control coefficients are set to *c*_1_ = 0.4, *c*_2_ = 0.4, and *c*_3_ = 0.2, assigning greater importance to population diversity and fitness improvement while retaining a smaller contribution from the optimization stage.

The coefficients satisfy:

*c*_1_ + *c*_2_ + *c*_3_ = 1,  *c*_1_,*c*_2_,*c*_3_ ≥ 0 (44)

The first term increases APO influence when population diversity decreases, the second increases APO influence when fitness improvement becomes limited, and the third provides stronger APO influence during the early optimization stage. Consequently, the adaptive coefficient responds to both the spatial distribution of candidate solutions and the optimization progress.

The adaptive behavior of AC-APSO arises from the variation of *w*_APO,*t*_ across iterations, which is governed by the observed population diversity *D_t_* and fitness improvement *I_t_*, rather than from the control coefficients themselves. The coefficients *c*_1_, *c*_2_, and *c*_3_ are fixed weighting constants that determine the relative sensitivity of *w_APO_*_,*t*_ to each signal, in a manner analogous to the fixed decay rates of adaptive gradient-based optimizers, whose update behavior nevertheless adapts to the observed gradients. Equal weights were assigned to *c*_1_ and *c*_2_ because population diversity and fitness improvement provide complementary indicators of search stagnation, reflecting premature convergence in the solution space and stagnation in the objective space, respectively, and neither indicator was assumed a priori to be more informative than the other. A smaller weight was assigned to *c*_3_ because the iteration-dependent term follows a predetermined schedule that does not reflect the actual optimization state. Limiting its contribution to 0.2 ensures that 80% of the coefficient is determined by state-dependent signals, while the schedule term introduces a mild bias toward exploration during the early iterations. Furthermore, because *D_t_*, *I_t_*, and $1-\frac{t}{T}$ each lie within [0, 1], the constraint *c*_1_ + *c*_2_ + *c*_3_ = 1 guarantees that *w*_APO,*t*_ remains within [0, 1], so that the clipping operation in Equation (43) serves only as a numerical safeguard.

The final candidate is generated as:

(45) $$X_{i,t+1}=w_{\mathrm{APO},t}X_{i,t}^{\mathrm{APO}}+\left(1-w_{\mathrm{APO},t}\right)X_{i,t}^{\mathrm{SFOA}}$$

When *w_APO_*_,*t*_ approaches one, greater influence is assigned to the APO-generated candidate, promoting diversification. When *w_APO_*_,*t*_ approaches zero, greater influence is assigned to the SFOA-generated candidate, supporting focused refinement. Intermediate values allow both bio-inspired mechanisms to contribute cooperatively.

This adaptive coordination differentiates AC-APSO from a simple APO + SFOA combination. The relative contribution of the two search mechanisms is not fixed; instead, it changes according to the current optimization state. The adaptive mechanism therefore provides a dynamic exploration–exploitation balance during the hyperparameter optimization process.

Following candidate generation, the updated candidates are evaluated, and a convergence-change term is computed to determine whether the optimization has stagnated:

Δ*F_t_* = |*F_best_*_,*t*_ − *F_best_*_,*t*−1_| (46)

where Δ*F_t_* denotes the absolute change in global-best fitness between the current and previous iteration. This quantity is distinct from the fitness-improvement term *I_t_* in Equation (42): *I_t_* is computed from past iterations and used to determine the adaptive APO contribution *w_APO_*_,*t*_ for generating new candidates, whereas Δ*F_t_* is computed after candidate evaluation and used only for the early-stopping (patience) criterion. If Δ*F_t_* < *δ*, the stagnation counter is incremented (*q* = *q* + 1); otherwise, it is reset (*q* = 0). Optimization terminates when *q* ≥ *P*.

The updated continuous hyperparameters are constrained within their predefined feasible ranges using:

*X_i_*_,*j*,*t*+1_ = min[*U_j_*, max(*L_j_*, *X_i_*_,*j*,*t*+1_)] (47)

This operation ensures that all updated candidates remain within the feasible search space defined in Table 1.

Since batch size is a discrete parameter, the continuous optimization output is mapped to the nearest permissible value as:

(48) $$B_{i,t+1}=\underset{b\in \left\{16,32,64\right\}}{\mathrm{arg min}}\left|B_{i,t+1}^{*}-b\right|$$

where $B_{i,t+1}^{*}$ denotes the continuous batch-size output and *B_i_*_,*t*+1_ is the discrete batch-size value in {16, 32, 64} closest to $B_{i,t+1}^{*}$. This mapping ensures that the discrete nature of the batch-size parameter is maintained during candidate evaluation.

The updated candidates are evaluated using the validation fitness, and the global-best solution is updated according to:

(49) $$X_{\mathrm{best},t}=\left\{\begin{array}{cc} X_{i^{*},t}, & \underset{i}{\min}F_{i,t}<F_{\mathrm{best},t-1}\\ X_{\mathrm{best},t-1}, & \mathrm{otherwise} \end{array}\right.,i^{*}=\underset{i}{\arg\min}\;F_{i}$$

where *i*^∗^ is the index of the candidate with the lowest fitness among the entire updated population at iteration *t*. Accordingly, the global-best solution is updated only if the top-performing candidate in the current population achieves a lower fitness value than the previously stored global-best; otherwise, the global-best from the prior iteration remains unchanged.

The optimization process proceeds iteratively until the maximum number of iterations is attained or Δ*F_t_* remains below the predefined convergence threshold *δ* for the specified number of successive iterations, *P*. In this study, the population size is set to *N* = 20, the maximum number of optimization iterations is set to T = 30, the convergence threshold is set to 10^−4^, and an early-stopping patience of five consecutive iterations is used. Thus, optimization is terminated when Δ*F_t_* remains below 10^−4^ for five successive iterations, or when 30 iterations are completed.

The final optimized configuration is obtained as:

*X*^∗^ = [*η*^∗^, *B*^∗^, *d*^∗^, *λ*^∗^, *α*^∗^] (50)

where *η*^∗^, *B*^∗^, *d*^∗^, *λ*^∗^, and *α*^∗^ denote the optimized learning rate, batch size, dropout rate, weight-decay coefficient, and Focal Loss weighting coefficient, respectively. The corresponding Categorical Cross-Entropy coefficient is obtained as:

*β*^∗^ = 1 − *α*^∗^ (51)

where *β*^∗^ denotes the corresponding Categorical Cross-Entropy weighting coefficient. Therefore, the final hybrid-loss coefficients satisfy *α*^∗^ + *β*^∗^ = 1.

The optimized configuration is subsequently fixed and used for final RM-LBAT-Net training. Importantly, AC-APSO performs bio-inspired hyperparameter optimization, whereas Adam remains responsible for gradient-based optimization of the trainable network parameters. Thus, the two optimization processes operate at different levels: AC-APSO determines the training configuration, while Adam learns the network weights. AC-APSO therefore operates as an outer-level hyperparameter optimization process, while Adam performs the inner-level gradient-based optimization of the trainable RM-LBAT-Net parameters. Algorithm 1 shows the complete procedure of the proposed AC-APSO for automatically determining the optimal training and loss-related hyperparameters of RM-LBAT-Net. The overall flow of the proposed bio-inspired optimization process is presented in Figure 9.
**Algorithm 1.** AC-APSO for Hyperparameter OptimizationInput: Hyperparameter search space [*L*, *U*], population size *N* = 20, maximum iterations *T* = 30, control coefficients *c*_1_ = 0.4, *c*_2_ = 0.4, *c*_3_ = 0.2, fitness weights *w_A_* = *w_F_* = *w_C_* = 1/3, convergence threshold *δ* = 10^−4^, and patience *P* = 5. Output: Optimized hyperparameter configuration *X*^∗^.**Begin**  1.Initialize *N* candidate solutions within the predefined hyperparameter search space.  2.Train each candidate configuration on the training subset.  3.Evaluate each candidate using the RM-LBAT-Net validation subset.  4.Calculate validation accuracy, macro-F1 score, and AUC.  5.Calculate the fitness using the defined weighted fitness function.  6.Identify the candidate with the minimum fitness as the initial global-best solution.  7.**For** *t* = 1 to *T*:○Generate candidate solutions using the original APO search mechanisms guided by the global-best solution from the previous iteration.○Generate candidate solutions using the original SFOA search mechanisms guided by the global-best solution from the previous iteration.○Calculate population diversity *D_t_*.○Calculate relative fitness improvement *I_t_* using the fitness values from the two preceding iterations.○Calculate the adaptive APO contribution *w_APO_*_,*t*_.○Combine the APO and SFOA candidates.○Apply the boundary constraints.○Map the batch-size value to the nearest permissible value in {16, 32, 64}.○Train the updated candidates on the training subset.○Evaluate the updated candidates using the validation subset.○Calculate the updated fitness.○Update the global-best solution (select the candidate with minimum fitness across the population; retain the previous global-best if no improvement is found).○Calculate the convergence change Δ*F_t_* = |*F_best_*_,*t*_ − *F_best_*_,*t*−1_|.○If Δ*F_t_* is less than *δ*, set *q* = *q* + 1; otherwise, set *q* = 0.○If *q* ≥ *P*, terminate the optimization.
  8.End For  9.Return the final global-best solution *X*^∗^.**End**

#### 3.5.5. Training Strategy and Final Model Configuration

Following the AC-APSO optimization, the best hyperparameter configuration is fixed for final RM-LBAT-Net training. The trainable network parameters are optimized using the Adam optimizer, while the hybrid objective combines Focal Loss and Categorical Cross-Entropy (CCE) Loss.

The Focal Loss is expressed as:

(52) $$L_{F}=-\sum_{c=1}^{C}\alpha_{c}{\left(1-p_{c}\right)}^{\gamma }y_{c}\log\left(p_{c}\right)$$

where *C* is the number of output classes, *y_c_* is the ground-truth indicator for class *c*, *p_c_* is the predicted probability for class *c*, *α_c_* is the class-balancing factor, and *γ* is the focusing parameter.

The CCE Loss is defined as:

(53) $$L_{\mathrm{CE}}=-\sum_{c=1}^{C}y_{c}\log\left(p_{c}\right)$$

The final hybrid loss is as:

*L_hybrid_* = *α*^∗^*L_F_* + *β*^∗^*L_CE_* (54)

where *α*^∗^ and *β*^∗^ are the same AC-APSO-optimized Focal Loss and CCE weighting coefficients respectively.

The optimized parameters are fixed during final training. The model undergoes training for 50 epochs with continuous validation monitoring. The fixed architectural and training-independent settings are summarized in Table 2, the AC-APSO optimization settings are summarized in Table 3, whereas the AC-APSO-optimized training and loss-related hyperparameters are presented in Table 4.

The complete workflow of the developed RM-LBAT-Net framework is summarized in Algorithm 2.
**Algorithm 2.** RM-LBAT-Net Framework with AC-APSO-Based Hyperparameter Optimization**Input:** Dermoscopic melanoma image dataset *D* = {(*X_i_*,*y_i_*)} **Output:** Optimized and trained RM-LBAT-Net model for benign/malignant melanoma classification**Begin**  ❖**Dataset Acquisition**Load dermoscopic melanoma image dataset and construct dataset *D* = {(*X_i_*,*y_i_*)}.  ❖**Dataset Splitting**Load the predefined training and test subsets provided with the dataset. Use the predefined training subset for model development, with the stratified 85:15 training-validation split used for AC-APSO hyperparameter optimization and the resulting fixed configuration assessed through five-fold cross-validation. Reserve the predefined test subset for final independent evaluation.  ❖**Image Preprocessing**For each image *X_i_*:     ●Normalize pixel intensities.     ●Remove hair artifacts using DullRazor.     ●Apply CLAHE-based contrast enhancement.     ●$\mathrm{Generate}\;\mathrm{preprocessed}\;\mathrm{image}\;X_{i}^{\prime }$.  ❖**Data Augmentation**Apply online augmentation only to the training samples.  ❖**Dual-Stream Feature Extraction**
  **1.****Res2Net-50 Branch**
Perform shallow convolutional embedding. Split feature channels into multiple scale groups. Apply hierarchical residual multi-scale convolutions. Aggregate multi-scale representations. Generate local feature representation.  **2.****MaxViT-T Branch**Apply MBConv embedding. Perform local window self-attention. Perform global grid attention. Aggregate local and global contextual representations. Generate contextual feature representation.  ❖**Boundary-Aware Feature Refinement (DLBAM)**Project the Res2Net-50 and MaxViT-Tiny feature representations into a common spatial and channel dimension. Align and integrate the projected feature representations to construct the DLBAM input feature map. Extract lesion boundaries using Sobel filtering. Perform Laplacian edge enhancement. Apply learnable edge convolutions. Apply deformable convolution with learnable spatial offsets. Generate deformable boundary-attention maps using sigmoid activation. Refine the semantic features through element-wise attention. Generate boundary-aware feature representation.  ❖**Cross-Scale Adaptive Semantic Fusion (CSASF)**Project *F_l_*, *F_g_*, and *F_b_* into a common semantic space. Concatenate the aligned representations to construct a joint semantic representation. Apply GAP to obtain the input-specific semantic descriptor *z*(*x*). Process *z*(*x*) through a lightweight gating network. Generate three input-dependent fusion logits. Apply Softmax to generate input-dependent fusion coefficients *α*(*x*), *β*(*x*), and *γ*(*x*). Fuse the three semantic representations using: *F*_fusion_ = *α*(*x*)*F_l_* + *β*(*x*)*F_g_* + *γ*(*x*)*F_b_* Perform residual refinement. Apply channel recalibration. Generate refined semantic representation.  ❖**Classification**Apply GAP. Pass pooled features through FC layers. Apply Softmax activation. Generate classification probabilities.  ❖**AC-APSO Hyperparameter Optimization**Initialize the candidate population within the predefined hyperparameter search ranges. For each candidate configuration:  ●Configure RM-LBAT-Net using the candidate hyperparameters.  ●Train the candidate model using Adam for gradient-based optimization of network parameters.  ●Apply the hybrid Focal Loss–CCE objective.  ●Evaluate validation accuracy, macro-F1 score, and AUC.  ●Calculate the candidate fitness.**For** *t* = 1 to *T*:  ●Generate APO-based candidate solution.  ●Generate SFOA-based candidate solution.  ●Calculate population diversity *D_t_* and fitness improvement *I_t_*.  ●Determine the adaptive APO contribution *w*_APO,*t*_  ●Combine the APO and SFOA candidates: $X_{i,t+1}=w_{\mathrm{APO},t}X_{i,t}^{\mathrm{APO}}+\left(1-w_{\mathrm{APO},t}\right)X_{i,t}^{\mathrm{SFOA}}.$  ●Apply boundary constraints and batch-size mapping.  ●Train and evaluate the updated candidates.  ●Update the population and global-best solution.  ●Calculate the convergence change Δ*F_t_*; increment the stagnation counter *q* if Δ*F_t_* < *δ*, otherwise reset *q* = 0.  ●If *q* ≥ *P*, terminate the optimization early.**End For** Obtain the optimal hyperparameter configuration *X*^∗^.  ❖**Final Training**Fix the optimized configuration *X*^∗^ and train RM-LBAT-Net using Adam and the hybrid Focal Loss–CCE objective.  ❖**Prediction**Predict lesion category:     ○Benign     ○Malignant  ❖**Model Evaluation**Evaluate the optimized RM-LBAT-Net on the independent test subset using the predefined performance metrics.  ❖**Model Deployment****End**

### 3.6. Training Configuration

The final RM-LBAT-Net is trained using the optimized configuration obtained through AC-APSO. Whereas each candidate configuration was trained for 10 epochs during AC-APSO-based optimization to limit computational cost, the final selected configuration is trained for 50 epochs using Adam, with continuous monitoring of validation performance. Online augmentation is applied only to the training samples, whereas the validation and test samples remain unaugmented. The optimized parameters are retained throughout final training. The fixed architectural parameters of RM-LBAT-Net are maintained without modification. After final training, the optimized model is evaluated on the independent test subset using the performance measures described in the subsequent evaluation section. The optimization and training procedures are maintained as two distinct stages: AC-APSO performs outer-level hyperparameter optimization using validation performance, whereas Adam performs inner-level gradient-based optimization of the trainable RM-LBAT-Net parameters. The external ISIC 2016 dataset was excluded from hyperparameter optimization and model selection and was reserved exclusively for external validation.

### 3.7. Implementation Details

Python was used to develop the proposed RM-LBAT-Net architecture, while TensorFlow 2.15.0 with the Keras API was used for network implementation, training, and experimental evaluation. All experiments, including AC-APSO-based hyperparameter optimization and final model training, were conducted on the Google Colaboratory platform using an NVIDIA Tesla T4 GPU (NVIDIA Corporation, Santa Clara, CA, USA) with 16 GB of VRAM and 32 GB of system memory. The computational environment employed Python 3.10, CUDA 12.2, and cuDNN 8.9, providing GPU-accelerated execution for tensor operations, convolutional processing, and neural network training. Owing to their computational cost, the individual optimization runs were executed in parallel across multiple Colab sessions, each using the same GPU configuration, with long runs resumed from saved checkpoints. Each optimization run comprises at most 620 candidate evaluations of 10 training epochs followed by validation, corresponding to approximately 6200 training epochs per run. The additional computation introduced by the adaptive coordination mechanism, namely the calculation of population diversity and fitness improvement at each iteration, is negligible compared with candidate training. Since the optimization is performed only once and offline, it does not affect the inference cost of the final model. The same software and hardware configuration, together with the fixed random seed of 42, was maintained throughout the optimization, ablation, cross-validation, and final evaluation experiments to ensure consistency of the reported results.

### 3.8. Evaluation Metrics

The efficacy of the proposed AC-APSO-optimized RM-LBAT-Net framework is assessed through a range of performance metrics, as:

(55) $$\mathrm{Accuracy}=\frac{\mathrm{TP}+\mathrm{TN}}{\mathrm{TP}+\mathrm{TN}+\mathrm{FP}+\mathrm{FN}}$$

(56) $$\mathrm{Precision}=\frac{\mathrm{TP}}{\mathrm{TP}+\mathrm{FP}}$$

(57) $$\mathrm{Recall}=\frac{\mathrm{TP}}{\mathrm{TP}+\mathrm{FN}}$$

(58) $$F1\text{-}\mathrm{Score}=\frac{2\times \mathrm{Precision}\times \mathrm{Recall}}{\mathrm{Precision}+\mathrm{Recall}}$$

where *TP*, *TN*, *FP*, and *FN* correspond to malignant lesions correctly classified as malignant, benign lesions correctly classified as benign, benign lesions incorrectly classified as malignant, and malignant lesions incorrectly classified as benign, respectively. In this binary classification task, the malignant class is considered the positive class, while the benign class is considered the negative class; accordingly, precision, recall, and F1-score are computed with respect to the malignant class. To quantify the uncertainty of the reported estimates, 95% confidence intervals were computed using the Wilson score interval for accuracy, precision, and recall, and a bootstrap procedure with 100,000 resamples of the confusion matrix for the F1-score.

## 4. Results and Discussions

### 4.1. Training and Validation

The accuracy curves of the proposed AC-APSO-optimized RM-LBAT-Net are presented in Figure 10, illustrating the learning progression and convergence behavior of the model over 50 epochs. At the beginning of training, the training and validation accuracies are approximately 0.49 and 0.54, respectively, in the initial epoch. The steady improvement in both accuracy values as training advances demonstrates that the network is progressively learning discriminative features characteristic of the dermoscopic images. By epoch 10, the training and validation accuracies reach approximately 0.80 and 0.79, respectively. After epoch 20, both curves exceed 0.95 and continue to converge toward the final training stages. The near-overlap of both curves observed in later epochs suggests that the optimized framework converges stably, avoids significant overfitting, and generalizes consistently.

The loss curves of the proposed AC-APSO-optimized RM-LBAT-Net are presented in Figure 11, illustrating the convergence behavior of the training process over 50 epochs. At the beginning of training, the training and validation losses are approximately 0.98 and decrease sharply during the initial epochs, indicating effective optimization of the network parameters. After epoch 10, the training and validation loss values reach approximately 0.25 and 0.32, respectively. As training progresses, both losses continue to decrease, reaching approximately 0.02–0.03 around epoch 25, indicating stable convergence under the optimized training configuration and hybrid loss objective. During the final epochs, the loss curves remain stable with minimal fluctuation. At the 50th epoch, training loss reaches roughly 0.02, while validation loss stabilizes near 0.01, demonstrating consistent convergence of the optimized model with limited divergence between the training and validation behavior.

### 4.2. Model Performance

Table 5 presents the class-wise classification efficiency of the developed AC-APSO-optimized RM-LBAT-Net framework. The optimized model achieves an overall accuracy of 98.85%, demonstrating its effectiveness in distinguishing benign and malignant melanoma lesions. The close agreement among the evaluated classification metrics indicates consistent predictive performance across the two lesion categories. For benign lesions, the precision, recall, and F1-score are 0.9900, 0.9870, and 0.9885, respectively, whereas the corresponding values for malignant lesions are 0.9870, 0.9900, and 0.9885, respectively.

The proposed AC-APSO-optimized framework achieves 98.85% accuracy, while the macro-averaged precision, recall, and F1-score also reach 98.85%. The comparable performance across the benign and malignant categories indicates balanced classification capability, with no substantial degradation toward either class. These findings collectively demonstrate the efficacy of integrating multi-scale feature extraction through Res2Net-50, global contextual representation through MaxViT-T, boundary-aware feature refinement using DLBAM, and adaptive semantic fusion through CSASF, together with AC-APSO-based optimization of the training configuration.

With an accuracy of 98.85%, along with precision, recall, and F1-score values of 98.70%, 99.00%, and 98.85%, respectively, the proposed AC-APSO-optimized RM-LBAT-Net framework demonstrates excellent classification performance in distinguishing benign from malignant cases. The slightly higher recall of 99.00% indicates the model’s strong capability to correctly identify malignant cases, while the high precision of 98.70% reflects a low rate of incorrect positive predictions. The F1-score of 98.85% further confirms a well-balanced trade-off between precision and recall. The corresponding 95% confidence intervals were 98.28–99.23% for accuracy, 97.80–99.24% for precision, 98.17–99.46% for recall, and 98.36–99.30% for the F1-score, indicating that the performance estimates remain consistently high within the statistical uncertainty associated with the 2000-image test set. Figure 12 presents the overall performance metrics of the proposed framework.

### 4.3. Misclassification and Error Analysis

Figure 13 presents the confusion matrix of the proposed AC-APSO-optimized RM-LBAT-Net framework. The optimized model correctly classifies 987 out of 1000 benign lesions and 990 out of 1000 malignant lesions, demonstrating strong discriminative capability and consistent lesion-sensitive representation learning. 13 benign lesions are incorrectly classified as malignant, whereas 10 malignant lesions are misclassified as benign, corresponding to a false-positive rate of 1.3% and a false-negative rate of 1.0%. The relatively low numbers of FP and FN predictions indicate that the proposed framework effectively distinguishes the two lesion categories. This performance can be attributed to the complementary multi-scale representation provided by Res2Net-50, global contextual learning through MaxViT-T, boundary-aware refinement using DLBAM, adaptive semantic fusion through CSASF, and the optimized training configuration obtained using AC-APSO.

Although limited in number, the two error types differ considerably in clinical consequence. The 13 false-positive cases would, in a clinical workflow, lead to additional examination or unnecessary excision of benign lesions, whereas the 10 false-negative cases represent missed melanomas, which may delay diagnosis and treatment and are therefore the more clinically critical error. False-negative predictions in dermoscopic melanoma classification typically arise from malignant lesions that lack the characteristic features emphasized by the model, such as early-stage or small melanomas with relatively regular borders, lesions with limited pigment variation, and lesions whose dermoscopic appearance closely resembles that of benign nevi. Because RM-LBAT-Net explicitly emphasizes boundary irregularity and pigmentation heterogeneity through DLBAM and CSASF, melanomas exhibiting weak boundary or color cues may provide insufficient discriminative evidence for correct classification. However, since the dataset does not provide clinical metadata, histopathological subtype, or lesion-thickness information, a subtype-specific analysis of these errors could not be performed. In clinical screening applications, where sensitivity is prioritized, the decision threshold could be adjusted to further reduce false-negative predictions at the cost of a moderate increase in false positives.

### 4.4. ROC Curve Analysis

Figure 14 presents the ROC analysis of the proposed AC-APSO-optimized RM-LBAT-Net classifier. The ROC curve closely follows the upper-left region of the plot, indicating high sensitivity with a low FP rate across the classification thresholds. The obtained AUC of 0.9996 demonstrates the strong discriminative capability of the optimized classifier in distinguishing benign and malignant melanoma lesions.

### 4.5. Prediction Visualization

Figure 15 presents representative prediction cases obtained from the proposed AC-APSO-optimized RM-LBAT-Net using the independent test dataset. The optimized framework achieves accurate classification across lesions exhibiting variations in shape, pigmentation, border characteristics, texture, and morphology. These predictions demonstrate the ability of the framework to learn discriminative melanoma-sensitive representations under challenging imaging conditions, including variations in illumination and the presence of hair artifacts. The observed classification performance is supported by the complementary multi-scale, contextual, and boundary-aware representations learned through Res2Net-50, MaxViT-T, and DLBAM, together with adaptive semantic fusion through CSASF and the optimized training configuration obtained using AC-APSO.

### 4.6. Grad-CAM Analysis for Visualizing Model Decisions

Figure 16 presents Grad-CAM visualizations, which identify the image areas contributing most to the classification outcomes of the proposed AC-APSO-optimized RM-LBAT-Net. Grad-CAM was applied to the final spatial feature representation after CSASF-based semantic fusion, residual refinement, and channel recalibration, immediately before the final GAP operation. This layer was selected because it retains spatially distributed high-level semantic information while remaining directly connected to the final classification decision. The high-intensity regions represented by red and yellow indicate stronger activation contributions to the model’s prediction, while blue regions correspond to comparatively lower activation. A qualitative assessment of the activation patterns reveals three consistent characteristics. First, in all presented cases, the high-activation regions are confined to the lesion area, whereas the surrounding skin and the image periphery receive low activation, suggesting that the classification decisions are not driven by background content. Second, benign lesions exhibit compact, centrally concentrated activation, corresponding to their relatively uniform pigmentation and regular outline. Third, malignant lesions exhibit broader and multi-focal activation extending across regions of heterogeneous pigmentation and toward the lesion margins, which is consistent with the emphasis of DLBAM on irregular boundaries and of CSASF on the integration of local and global lesion characteristics. These patterns correspond to dermoscopic features commonly associated with melanoma, such as asymmetry, color variegation, and border irregularity.

The corresponding overlay visualizations further illustrate the spatial association between the model’s activations and the lesion regions in both classes. These observations provide qualitative evidence that the proposed framework bases its predictions primarily on lesion-associated visual information when distinguishing benign and malignant dermoscopic images, thereby providing qualitative insight into the model’s classification behavior. However, Grad-CAM heatmaps are coarse, post hoc approximations of model attention, and in this study, they were neither compared quantitatively against expert lesion annotations or segmentation masks nor evaluated by dermatologists. Therefore, these visualizations should be regarded as qualitative interpretability aids rather than as direct clinical validation of the model’s decision-making.

### 4.7. Ablation Study

To evaluate the impact of the architectural elements and the proposed AC-APSO-based hyperparameter optimization strategy, component-wise and optimization-wise ablation experiments were performed using the identical training data and evaluation protocol adopted for the proposed framework. The same preprocessing pipeline, training procedure, number of training epochs, and random seed were maintained across all configurations, with only the architectural components or the hyperparameter configuration under evaluation being varied, to ensure a consistent comparison. The ablation results were used to quantify the contribution of the individual architectural components and the proposed optimization strategy.

#### 4.7.1. Component-Wise Ablation Analysis

The component-wise ablation analysis evaluates the progressive impact of the primary architectural elements of RM-LBAT-Net. The experiments begin with the Res2Net-50 branch and progressively incorporate MaxViT-T, DLBAM, and CSASF. To separate the contribution of each architectural component from that of hyperparameter optimization, each architectural variant was evaluated under two training configurations. In the first, M1–M4 were trained using the same fixed, manually selected configuration, comprising a learning rate of 0.00020, a batch size of 32, a dropout rate of 0.20, a weight decay of 5 × 10^−6^, and loss weights of α = 0.60 and β = 0.40. In the second, the corresponding variants A1–A4 were trained using the configuration selected by AC-APSO for the complete RM-LBAT-Net, comprising a learning rate of 0.00032, a batch size of 32, a dropout rate of 0.25, a weight decay of 1 × 10^−5^, and loss weights of α = 0.70 and β = 0.30. Under both configurations, all variants were trained for 50 epochs with the same random seed and evaluated on the same independent test subset. Because the optimized configuration was selected for the complete architecture, it is not necessarily optimal for the reduced variants; the comparison therefore reflects the contribution of each component under a common optimized setting. The resulting performance and the corresponding confusion-matrix counts are summarized in Table 6.

Under the fixed configuration, the baseline configuration employing only the Res2Net-50 backbone (M1) yields an accuracy of 90.10%, with corresponding precision, recall, and F1-score of 90.18%, 90.00%, and 90.09%, respectively, underscoring the efficacy of multi-scale feature extraction in representing lesion characteristics. The incorporation of MaxViT-T (M2) results in a marked improvement, elevating the accuracy to 95.10%, with precision, recall, and F1-score of 95.19%, 95.00%, and 95.10%, respectively, thereby validating the benefit of integrating local multi-scale representations with global contextual information. Subsequent integration of DLBAM (M3) further enhances performance, increasing the accuracy to 97.10%, with precision, recall, and F1-score of 97.19%, 97.00%, and 97.10%, respectively, demonstrating the contribution of boundary-aware feature refinement to overall model efficacy. The addition of CSASF in M4 further improves the accuracy to 97.25%, with a precision of 97.49%, recall of 97.00%, and F1-score of 97.24%. Under the AC-APSO-selected configuration, the same progressive trend is observed, with accuracy increasing from 93.25% for A1 to 96.35% for A2, 98.05% for A3, and 98.85% for the complete RM-LBAT-Net (A4), which also achieves a precision of 98.70%, recall of 99.00%, and F1-score of 98.85%. The incorporation of MaxViT-T, DLBAM, and CSASF improves accuracy by 5.00, 2.00, and 0.15 percentage points under the fixed configuration and by 3.10, 1.70, and 0.80 percentage points under the optimized configuration, respectively, indicating that each component contributes under both training settings and that the contribution of CSASF becomes more evident under the optimized configuration. In addition, the optimized configuration improves the accuracy of every architectural variant, with gains of 3.15, 1.25, 0.95, and 1.60 percentage points for the four variants. The confusion-matrix counts further show that the number of misclassified malignant lesions decreases from 100 in M1 to 10 in A4. Overall, these results indicate that the performance of the complete framework arises from the combination of the architectural components and AC-APSO-based hyperparameter optimization rather than from either factor alone. The corresponding confusion matrices of M1–M4 and A1–A4 are provided in Supplementary Figures S1 and S2, respectively.

#### 4.7.2. Optimization-Wise Ablation Analysis

The optimization-wise ablation study evaluates the contribution of the proposed AC-APSO strategy while keeping the complete RM-LBAT-Net architecture unchanged. All optimization strategies were evaluated under identical conditions, including the same population size, number of iterations, candidate-training budget, search space, fitness function, and random seed. The non-adaptive combination O4 combined the APO- and SFOA-generated candidates with a fixed equal weight of 0.5, whereas AC-APSO updates this weight at each iteration according to the optimization state. The configuration selected by each strategy was subsequently trained for 50 epochs and evaluated on the same independent test subset. The results and the corresponding confusion-matrix counts are presented in Table 7.

As in Table 7, the non-optimized configuration (O1), which corresponds to the fixed, manually selected configuration of M4 in the component-wise ablation, achieves an accuracy of 97.25%, providing the baseline performance of the complete RM-LBAT-Net without hyperparameter optimization. Applying APO (O2) improves the accuracy to 97.50%, demonstrating the benefit of bio-inspired hyperparameter search. SFOA (O3) further improves the accuracy to 97.75%, indicating its effectiveness in identifying favorable hyperparameter configurations. The combined APO + SFOA configuration (O4) achieves 98.20% accuracy, demonstrating the benefit of utilizing complementary search mechanisms. The proposed AC-APSO configuration (O5) ultimately achieves an accuracy of 98.85% and corresponding precision, recall, and F1-score of 98.70%, 99.00%, and 98.85%. The same ordering is reflected in the best validation fitness, which decreases from 0.112 for O1 to 0.094, 0.082, 0.069, and 0.058 for O2–O5, respectively, indicating that the improvements observed on the test subset follow the validation-based optimization objective. Correspondingly, the number of misclassified malignant lesions decreases from 30 in O1 to 10 in O5. The improvement from O4 to O5 demonstrates the contribution of the adaptive coordination mechanism in dynamically balancing APO-based diversification and SFOA-based refinement during hyperparameter optimization. AC-APSO continued to improve the best validation fitness until the final iteration, whereas APO, SFOA, and the fixed combination reached their best fitness at iterations 24, 26, and 28, respectively, at the cost of a moderate increase in optimization time. Configurations O2–O5 span the range from the individual search mechanisms (O2 and O3) to a fixed equal combination (O4) and the state-dependent adaptive combination (O5), all evaluated under identical conditions. Because O4 and O5 differ only in whether the weight is fixed or updated according to the optimization state, their comparison isolates the effect of the adaptive coordination mechanism, which reduced the best validation fitness from 0.069 to 0.058 and the number of misclassified malignant lesions from 19 to 10. As each optimization strategy was executed once, owing to the computational cost of a single run, the comparison reflects single-run outcomes and does not quantify the run-to-run variability inherent to stochastic metaheuristics. Nevertheless, the consistent ranking of the five configurations across the best validation fitness and all test metrics supports the reliability of the observed trends. The corresponding confusion matrices of O1–O5 are provided in Supplementary Figure S3.

#### 4.7.3. Convergence Analysis of AC-APSO

Figure 17 illustrates the convergence behavior of the proposed AC-APSO hyperparameter optimization strategy over 30 iterations. Since the optimization problem is formulated as a minimization objective, lower fitness values correspond to better hyperparameter configurations. The fitness value decreases rapidly during the initial iterations, from approximately 0.176 at iteration 1 to 0.100 at iteration 10, indicating rapid improvement in the candidate solutions during the early optimization stage. Between iterations 10 and 20, the fitness continues to decrease from 0.100 to 0.071, demonstrating further refinement of the candidate hyperparameter configurations. After iteration 20, the rate of improvement gradually decreases, with the fitness reaching approximately 0.058 at iteration 30. This progressively smaller change in fitness indicates convergence toward a stable low-fitness configuration. Overall, the continuously decreasing and gradually stabilizing fitness curve demonstrates the convergence behavior of the proposed AC-APSO strategy for hyperparameter optimization of RM-LBAT-Net. Because each candidate was trained for 10 epochs during optimization, the fitness values in Figure 17 reflect reduced-budget validation performance and are therefore higher than the error levels attained by the final configuration trained for 50 epochs.

### 4.8. K-Fold Cross-Validation

A five-fold cross-validation was performed exclusively on the development data to assess the stability of the selected RM-LBAT-Net configuration. The independent 2000-image test subset was completely excluded from cross-validation, hyperparameter optimization, model selection, and all training procedures and was assigned entirely for the final independent evaluation. The development data were divided into five stratified folds, with four folds used for model training and the remaining fold serving as the held-out fold in each iteration. The hyperparameter configuration obtained through the development-stage AC-APSO optimization procedure described in Section 3.5.4 was held fixed across all five folds. Thus, cross-validation was performed as a fixed-configuration stability assessment rather than as an independent hyperparameter-selection procedure, and AC-APSO was not re-optimized within individual folds. The held-out fold was not used for model training in the corresponding iteration. The same preprocessing procedures and training-set-only data augmentation strategy were consistently applied across all folds. The resulting fold-wise performance is presented in Table 8.

Fold-wise accuracies of 98.6%, 98.7%, 98.9%, 98.8%, and 99.0% were achieved, indicating consistent performance across the five development-data partitions. Across the five folds, the model achieved a mean accuracy of 98.80 ± 0.16%, precision of 98.30 ± 0.16%, recall of 97.94 ± 0.11%, F1-score of 98.10 ± 0.16%, and AUC of 0.9996 ± 0.0002, reported as mean ± standard deviation, where the standard deviation was computed as the sample standard deviation across the five folds. The standard deviations of accuracy, precision, recall, and F1-score remained below 0.2 percentage points, the fold-wise AUC varied only within 0.9992–0.9998, and the fold-wise accuracy varied only within 98.6–99.0%, indicating limited variation in performance across the folds and suggesting that the observed results are not strongly dependent on a particular data partition.

The curves presented in Figure 18a–e illustrate the learning behavior and convergence stability of the AC-APSO-optimized RM-LBAT-Net across the five cross-validation folds. Figure 18a shows progressive increases in training and validation accuracy during the initial epochs, accompanied by a corresponding reduction in loss. Figure 18b demonstrates smooth convergence with a small difference between training and validation performance. Figure 18c shows comparatively higher validation accuracy during the later epochs, whereas Figure 18d exhibits stable accuracy improvement and progressively decreasing loss. Figure 18e similarly demonstrates smooth convergence with limited fluctuations between training and validation performance. Overall, the learning curves provide qualitative evidence of consistent convergence across the five folds and limited sensitivity to the particular data partition. Importantly, these cross-validation results represent an assessment of model stability on the development data and should not be interpreted as an additional evaluation of the independent test set.

### 4.9. Hyperparameter Sensitivity Analysis

A sensitivity analysis was performed to assess the influence of the hyperparameters chosen by AC-APSO on the classification efficacy of RM-LBAT-Net, wherein each hyperparameter was varied independently while the other parameters remained fixed at their optimum settings. All hyperparameter sensitivity experiments were conducted using the development/validation data, while the independent test subset was reserved exclusively for the final evaluation. The selected hyperparameter configuration was fixed before evaluation on the independent test subset.

The sensitivity analysis demonstrates that RM-LBAT-Net performs most effectively at the hyperparameter configuration selected by AC-APSO within the investigated ranges. As shown in Table 9, for the learning rate, performance improves as the learning rate increases toward the selected value of 0.00032, achieving the highest observed validation accuracy of 98.82% and macro-F1 of 98.81%. Further increases result in a gradual performance decline. Regarding batch size, the selected value of 32 yields the highest observed validation performance, whereas batch sizes of 16 and 64 produce slightly lower results. For the dropout rate, performance increases toward the selected value of 0.25, while higher dropout rates result in slightly lower performance. The weight-decay analysis shows that the selected value of 1 × 10^−5^ provides the highest observed validation performance. For the Focal Loss weighting coefficient, α = 0.70 achieves the highest observed validation performance compared with the neighboring values investigated. Overall, the results suggest that the AC-APSO-selected configuration (learning rate 0.00032, batch size 32, dropout rate 0.25, weight decay 1 × 10^−5^, Focal Loss weight α = 0.70, and CCE weight β = 0.30) provides a favorable operating point within the investigated hyperparameter ranges.

### 4.10. External Dataset Validation Using ISIC

To examine the cross-dataset generalization capability of the proposed model, external validation was carried out using the ISIC 2016 dataset [40]. The lesion-classification task defines two classes: benign, representing non-malignant lesions, and malignant, representing melanoma. This binary class definition is consistent with the benign-versus-malignant classification objective adopted in the primary dataset. The AC-APSO-optimized RM-LBAT-Net model, trained exclusively on the primary dermoscopic dataset, was directly evaluated on the 379-image ISIC 2016 test set without additional retraining or fine-tuning. The external test data were excluded from model training, validation, hyperparameter optimization, and model selection. The same preprocessing pipeline, including image resizing to 224 × 224 pixels and the preprocessing operations was applied before inference, while the learned model parameters remained unchanged. The predictions were evaluated against the corresponding ISIC 2016 ground-truth malignancy labels. As summarized in Table 10, the model attained an accuracy of 97.36%, precision of 90.12%, recall of 97.33%, and F1-score of 93.59%; the comparatively wide confidence intervals reflect the smaller size of the external test set, particularly the limited number of malignant cases. The corresponding confusion matrix is shown in Figure 19. These results provide preliminary evidence of promising cross-dataset generalization, while further evaluation on larger and more diverse multi-center datasets is warranted [41].

The generalization results obtained on the independent test subset and the external dataset are also relevant to the selection stage of AC-APSO. Since the fitness of each candidate configuration was computed on the same validation subset throughout the optimization process, the validation-based estimates used to rank candidates may be optimistically biased toward that subset. This effect is confined to the selection stage, as all performance measures reported in this study were obtained on data that were not involved in optimization or model selection, namely the independent 2000-image test subset and the external ISIC 2016 dataset. Any adaptation of the selected configuration to the validation subset would therefore be reflected as reduced test performance rather than as an inflated performance estimate. The stability of the selected configuration across five development-data partitions (98.80 ± 0.16% accuracy) and its accuracy of 97.36% on the external dataset indicate that its effectiveness is not specific to the validation subset used during optimization. The restriction of the search space to five training-related hyperparameters and the use of a fitness function combining three complementary metrics further limit the extent to which the selection process can exploit random fluctuations in a single validation measure. Nested cross-validation, in which the complete optimization is repeated within each outer fold, would allow the magnitude of this selection effect to be quantified directly; however, it would require approximately 3100 candidate trainings across five outer folds, corresponding to more than 250 h of GPU computation, which was not computationally feasible in the present study. Similarly, reserving an additional hold-out subset exclusively for final model selection would require further partitioning of the 1781-image validation subset, reducing the number of images available for fitness evaluation and thereby increasing the variance of the validation estimates that guide the optimization.

### 4.11. Head-to-Head Performance Benchmarking

To estimate the efficacy of the proposed melanoma classification framework, a head-to-head comparison was performed with representative CNN-based, transfer-learning, and transformer-based architectures, including the contemporary ConvNeXtV2-Tiny and Swin Transformer V2-Tiny models, under a common evaluation protocol. All baseline models were trained using the same development dataset, preprocessing pipeline, input resolution of 224 × 224 pixels, training-only online augmentation, and binary classification task as the proposed framework. ResNet50, EfficientNet-B0, ConvNeXtV2-Tiny, and Swin Transformer V2-Tiny were initialized with ImageNet-pretrained weights and fine-tuned on the melanoma dataset. The ViT model was similarly initialized with weights pretrained on ImageNet and subsequently fine-tuned for the target classification task. The custom CNN was trained from random initialization. For all baseline models, the final classification layer was configured for two output classes using Softmax activation. To ensure a consistent comparison, the baseline models were trained using Adam with a learning rate of 0.00032, a batch size of 32, and 50 training epochs. The same training-set augmentation was applied to the baseline models, while validation and test images remained unaugmented. No additional hyperparameter optimization was performed for the baseline models. The baseline configurations were fixed before evaluation, and the same random seed of 42 was used for model initialization, data ordering, and other stochastic operations. For the transfer-learning models, the pretrained backbone parameters were fine-tuned rather than kept permanently frozen, with the task-specific two-class classification head trained jointly with the backbone. To separate the contribution of the architecture from that of hyperparameter optimization, RM-LBAT-Net trained with the fixed configuration (M4) is also included in the comparison. The baseline models were evaluated using the same independent test subset and the same performance measures used for RM-LBAT-Net. In addition, 95% confidence intervals of accuracy were computed using the Wilson score method to quantify the uncertainty of the test-set estimates.

Among the baseline architectures evaluated in Table 11, Swin Transformer V2-Tiny delivers the best performance with an accuracy of 97.05%, followed by ConvNeXtV2-Tiny at 96.40%, ViT at 95.65%, and EfficientNet-B0 at 94.80%, whereas ResNet50 and the custom CNN achieve 89.20% and 87.85%, respectively. The proposed AC-APSO-optimized RM-LBAT-Net achieves the highest performance, with an accuracy of 98.85%, precision of 98.70%, recall of 99.00%, and F1-score of 98.85%. Compared with the strongest contemporary model, Swin Transformer V2-Tiny, the proposed framework improves accuracy by 1.80 percentage points and reduces the number of misclassified malignant lesions from 32 to 10. Moreover, the 95% confidence interval of the proposed framework (98.28–99.23%) does not overlap with that of any compared model. When trained with the same fixed configuration, RM-LBAT-Net (M4) attains an accuracy of 97.25%, which is comparable to that of Swin Transformer V2-Tiny, with overlapping confidence intervals. This indicates that the advantage of the proposed framework arises from the combination of the RM-LBAT-Net architecture and AC-APSO-based hyperparameter optimization rather than from the architecture alone. This performance is associated with the complementary integration of Res2Net-50-based multi-scale feature extraction, MaxViT-T-based contextual representation learning, DLBAM-based boundary refinement, and CSASF-based adaptive semantic fusion, while its isolated contribution is examined separately in the ablation study.

### 4.12. Comparative Analysis with State-of-the-Art Methods

Table 12 presents a comparative analysis of the proposed melanoma classification framework with several recently reported approaches. The compared methods include transfer learning-based, conventional CNN-based, spiking neural network-based, and hybrid transformer-based architectures. DenseNet201, EfficientNet, Deep Spiking CNN, Xception, and the EfficientNet + ViT + Swin Transformer framework reported accuracies of 87.88%, 92.50%, 95.07%, 96.48%, and 96.90%, respectively, in their corresponding studies. The proposed AC-APSO-optimized RM-LBAT-Net achieved an accuracy of 98.85% under the experimental protocol adopted in the present study.

Recent studies have also explored diverse architectural and optimization strategies for dermoscopic lesion classification. ConvNeXt, Vision Transformer, and Swin Transformer models evaluated on the same Kaggle dermoscopic dataset employed in the present study achieved a best accuracy of 91.50% [28], whereas a hybrid framework integrating U-Net-based segmentation, Inception-ResNet-v2, and a Vision Transformer achieved an accuracy of 98.65% on the ISIC 2020 dataset [29]. Metaheuristic-optimized CNN classifiers have similarly been investigated for melanoma detection. A comparative analysis of CNN models optimized using CS, FA, WOA, PSO, GWO, and CSA reported a best accuracy of 91.25% [30], while a CNN optimized using a hybrid GWO–PSO strategy achieved an accuracy of 95.30% [31]. Furthermore, a morphology-guided attention network incorporating explainability and uncertainty estimation achieved an accuracy of 99.12% for seven-class skin lesion classification on the HAM10000 dataset [32], exceeding the accuracy obtained by the proposed framework; however, this evaluation was confined to a single dataset and addressed a different classification task.

The proposed framework demonstrates competitive performance relative to the reported results of the methods included in Table 12. However, this cross-study comparison should be interpreted with caution because the reported results were obtained under different experimental conditions, including variations in datasets, sample sizes, preprocessing procedures, training configurations, and evaluation protocols. Because the datasets differ in size, class composition, image sources, and classification difficulty, accuracy values obtained on different datasets are not directly comparable. Therefore, the accuracy values presented should not be interpreted as a strictly controlled head-to-head comparison. Rather, they provide an indication of the competitiveness of the proposed framework relative to the reported results of the selected existing approaches.

Under the experimental conditions adopted in this study, the performance of the proposed framework is attributed to the complementary integration of hierarchical multi-scale feature extraction, global contextual learning, deformable lesion-boundary refinement, input-dependent semantic fusion, and adaptive hyperparameter optimization. Res2Net-50 facilitates multi-scale lesion representation, while MaxViT-Tiny captures global contextual dependencies and lesion-wide structural relationships. DLBAM enhances boundary-related feature representation, whereas CSASF dynamically integrates local, global, and boundary-aware semantic information. In addition, AC-APSO determines the training configuration through adaptive coordination of the APO and SFOA search mechanisms. These components collectively contribute to the performance achieved by the proposed framework under the experimental conditions of this study.

## 5. Conclusions

This research introduced an AC-APSO-optimized RM-LBAT-Net framework for automated melanoma lesion classification from dermoscopic images. The framework integrates Res2Net-50-based multi-scale feature extraction, MaxViT-Tiny-based global contextual learning, DLBAM, and input-dependent CSASF, while AC-APSO determines the training configuration by adaptively coordinating the complementary search behaviors of APO and SFOA. The optimized framework achieved 98.85% accuracy, 98.70% precision, 99.00% recall, and 98.85% F1-score. The controlled ablation analyses showed that each architectural component contributed under both fixed and optimized training configurations, and that the adaptive weighting of AC-APSO outperformed the individual and fixed-weight combinations of APO and SFOA. Five-fold cross-validation further confirmed stable performance across different dataset partitions, with a mean accuracy of 98.80 ± 0.16%, a mean recall of 97.94 ± 0.11%, and a mean AUC of 0.9996 ± 0.0002. Grad-CAM visualizations indicated that the optimized framework primarily focused on lesion regions, providing qualitative insight into its classification behavior, although these visualizations do not constitute clinical validation of the model’s decision-making. External validation on the ISIC 2016 dataset yielded an accuracy of 97.36%, precision of 90.12%, recall of 97.33%, and F1-score of 93.59%, indicating that the framework maintains high sensitivity to malignant lesions on an independent dermoscopic dataset. In a head-to-head comparison under an identical experimental protocol, the proposed framework achieved higher performance than contemporary architectures, including ConvNeXtV2-Tiny and Swin Transformer V2-Tiny. The state-of-the-art comparison further showed that the proposed framework achieved competitive performance relative to the reported results of the approaches included in the comparative table. However, as these cross-study results were obtained under different datasets and experimental protocols, they indicate the competitiveness of RM-LBAT-Net rather than definitive evidence of superiority.

Despite these promising results, prospective clinical validation and evaluation across larger multi-institutional cohorts with diverse patient demographics, imaging devices, acquisition protocols, and rare melanoma subtypes have not yet been conducted. In addition, although no exact or pixel-identical duplicates were identified between the training and test sets, the primary dataset is provided at the image level without patient- or lesion-level identifiers; therefore, the possibility of near-duplicate images or multiple images of the same lesion across the partitions could not be fully ruled out. Furthermore, candidate configurations in AC-APSO were evaluated repeatedly on a single validation subset, and nested cross-validation, which would directly quantify the associated selection effect, was not performed owing to its computational cost. Similarly, each optimization strategy was evaluated in a single run, and the run-to-run variability of AC-APSO relative to the compared metaheuristics was therefore not quantified. Moreover, the dual-stream architecture and bio-inspired hyperparameter optimization increase computational requirements, which may limit deployment on resource-constrained clinical systems. Future research will therefore focus on prospective multi-institutional clinical validation, evaluation across broader patient and imaging populations with lesion-level data partitioning, nested cross-validation of the hyperparameter optimization procedure, evaluation of the optimization strategies over multiple independent runs with statistical significance testing, systematic evaluation of alternative control-coefficient configurations of AC-APSO, development of lightweight and computationally efficient variants for real-time applications, and integration of multimodal clinical information.

## Figures and Tables

**Figure 1:**
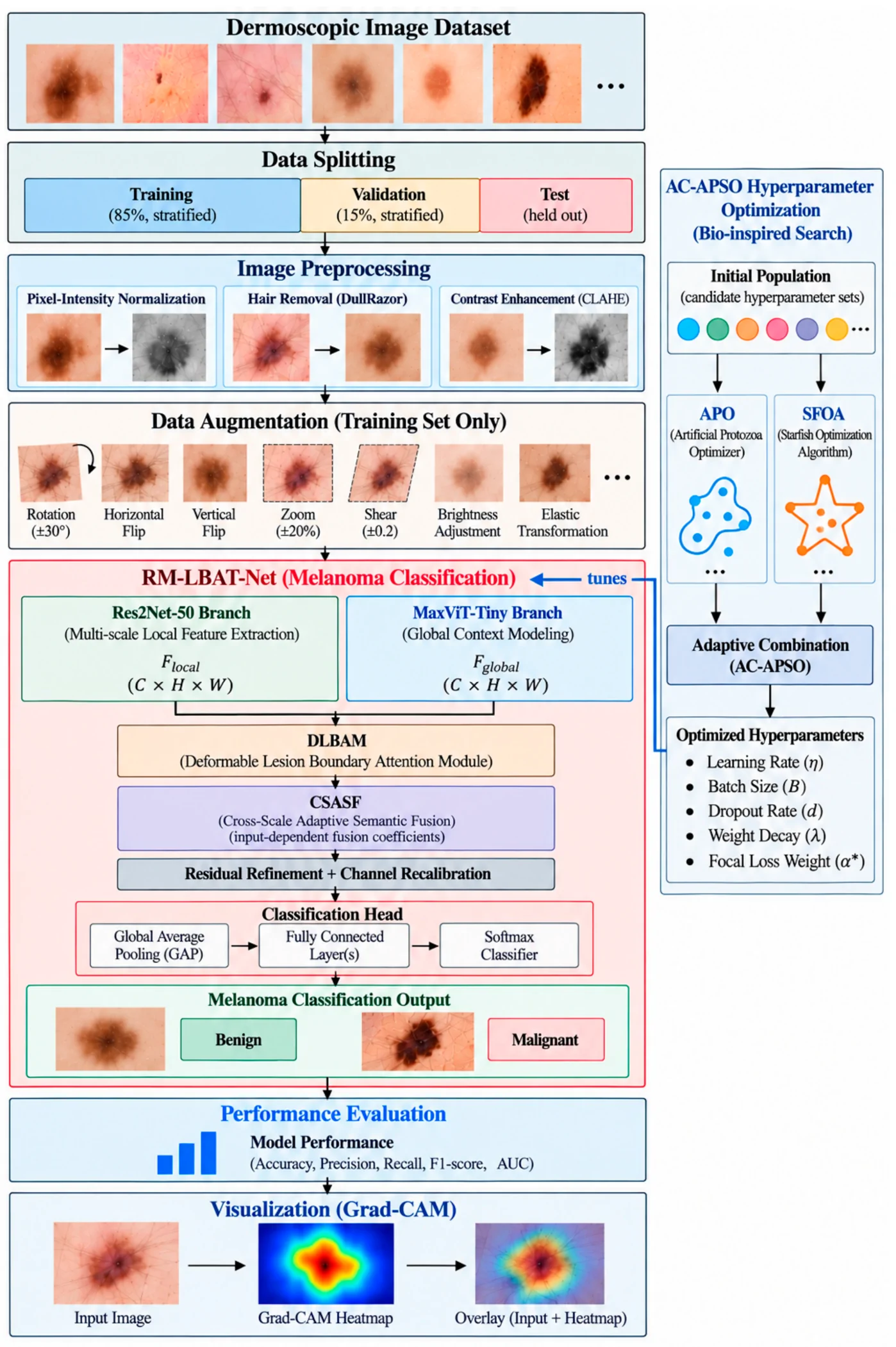
Overall pipeline of the proposed AC-APSO-optimized RM-LBAT-Net framework.

**Figure 2:**
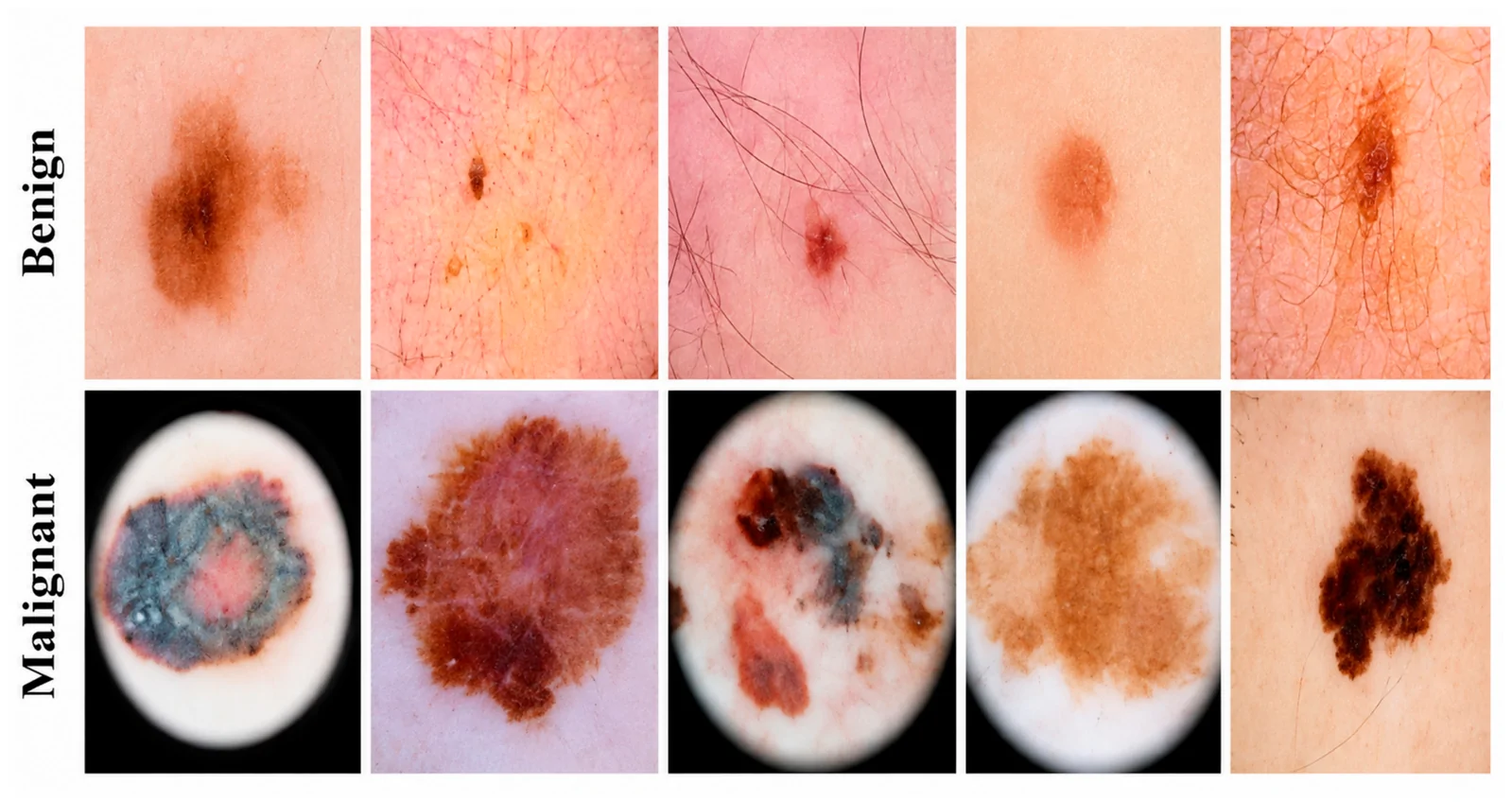
Sample images.

**Figure 3:**
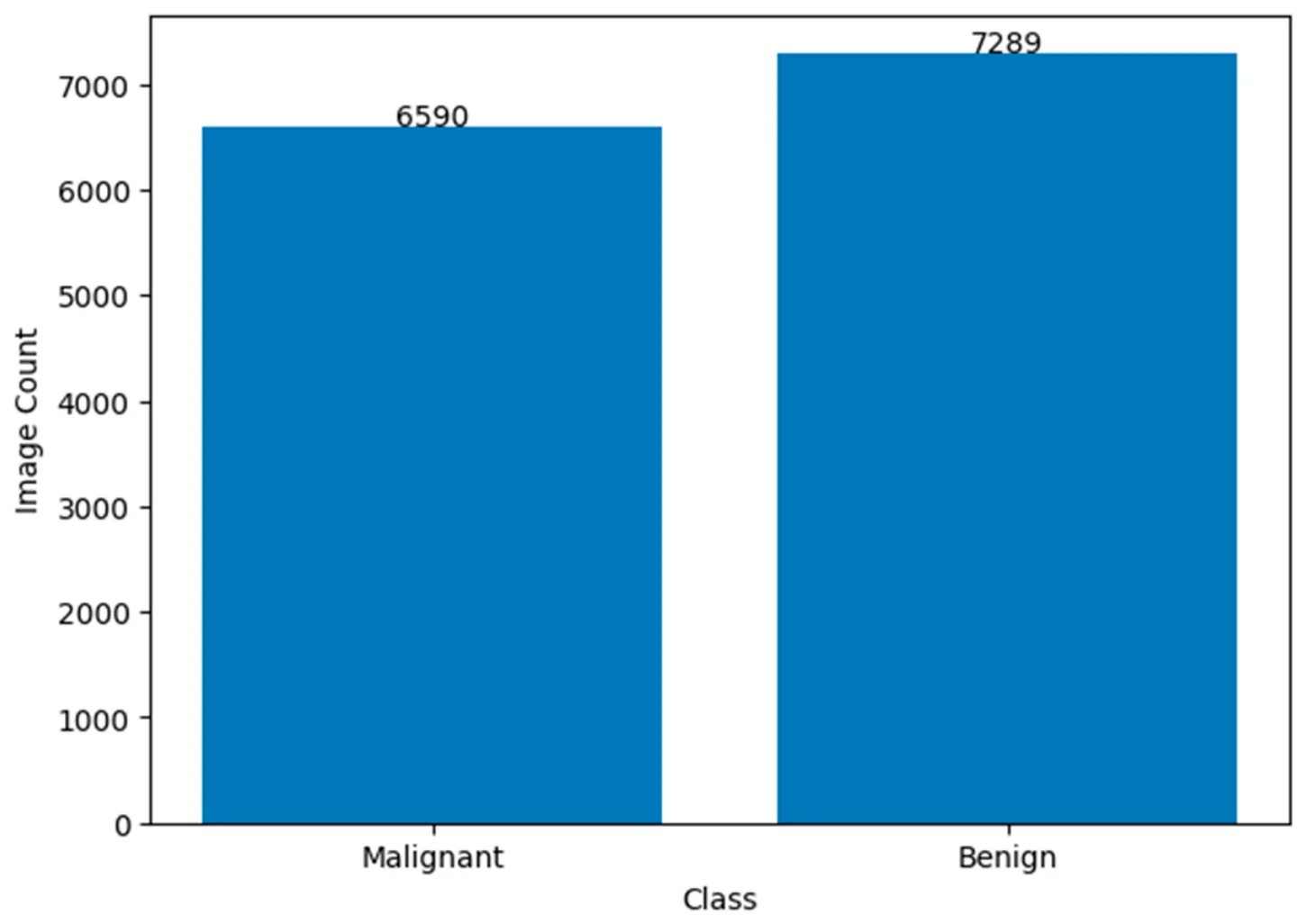
Class distribution of images.

**Figure 4:**
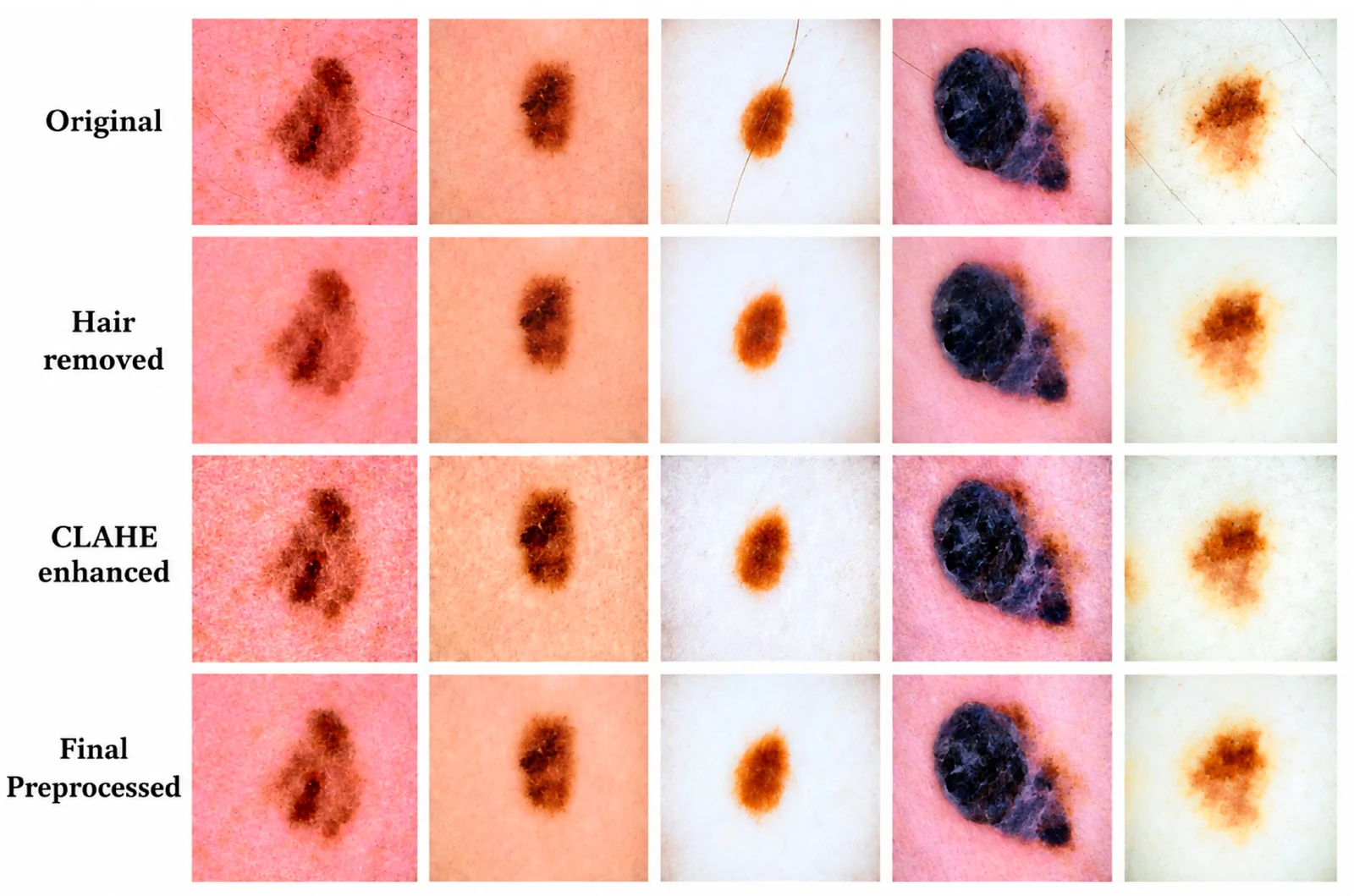
Sequential preprocessing stages.

**Figure 5:**
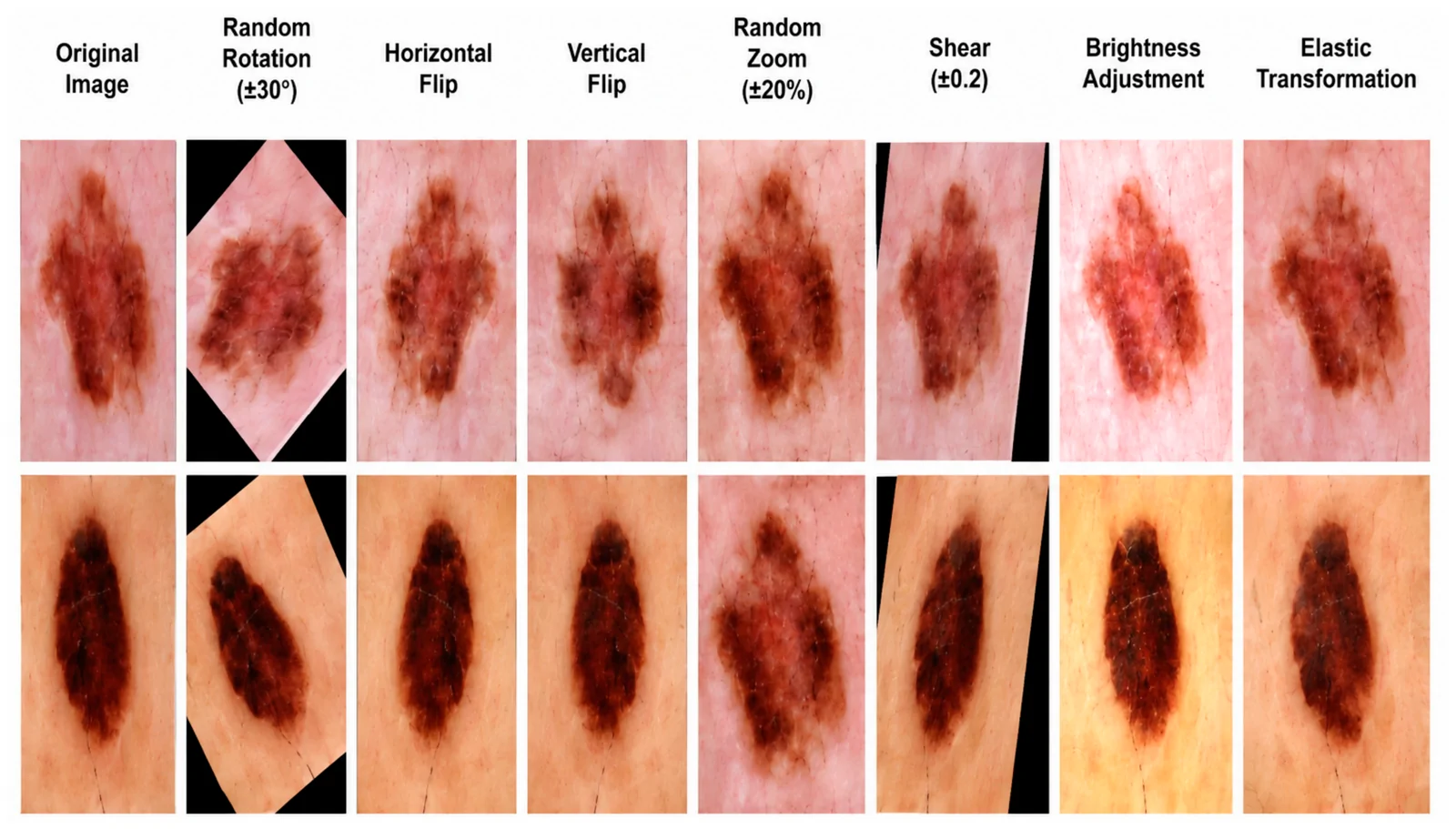
Representative images generated using online data augmentation.

**Figure 6:**
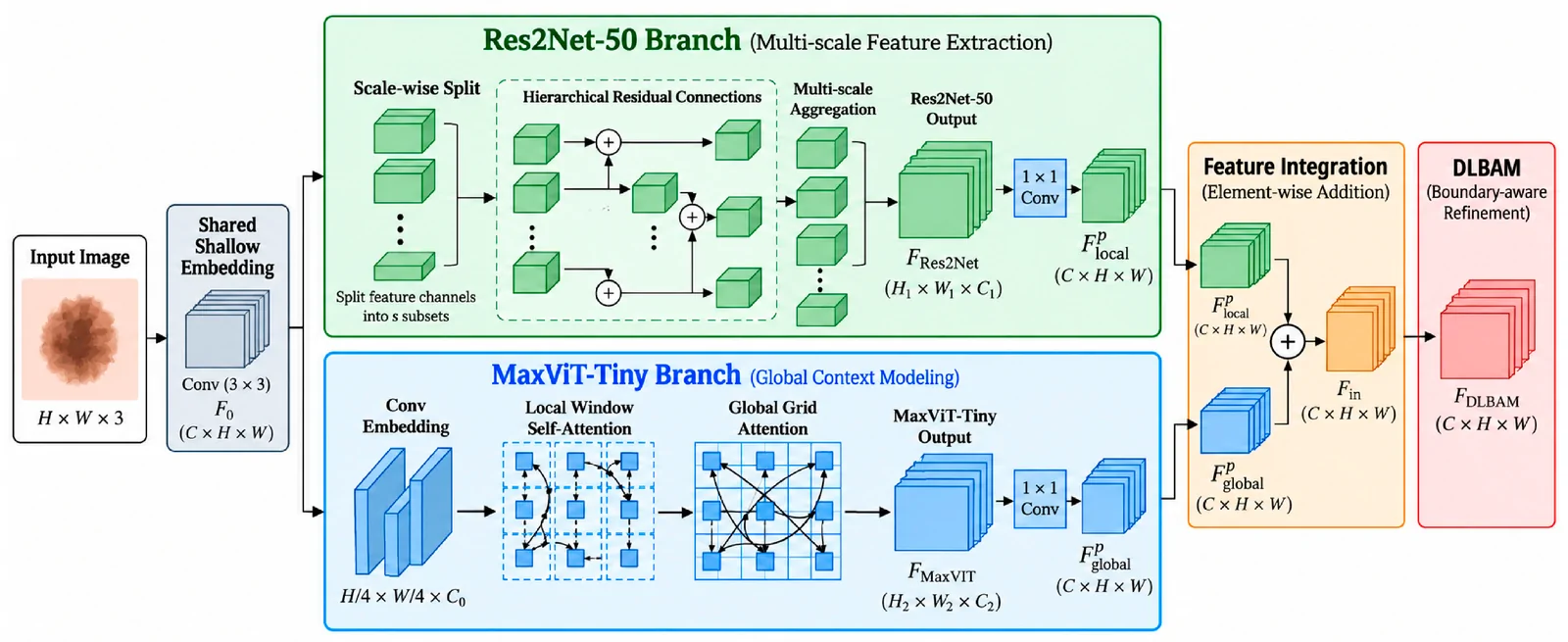
Dual-stream feature extraction framework.

**Figure 7:**
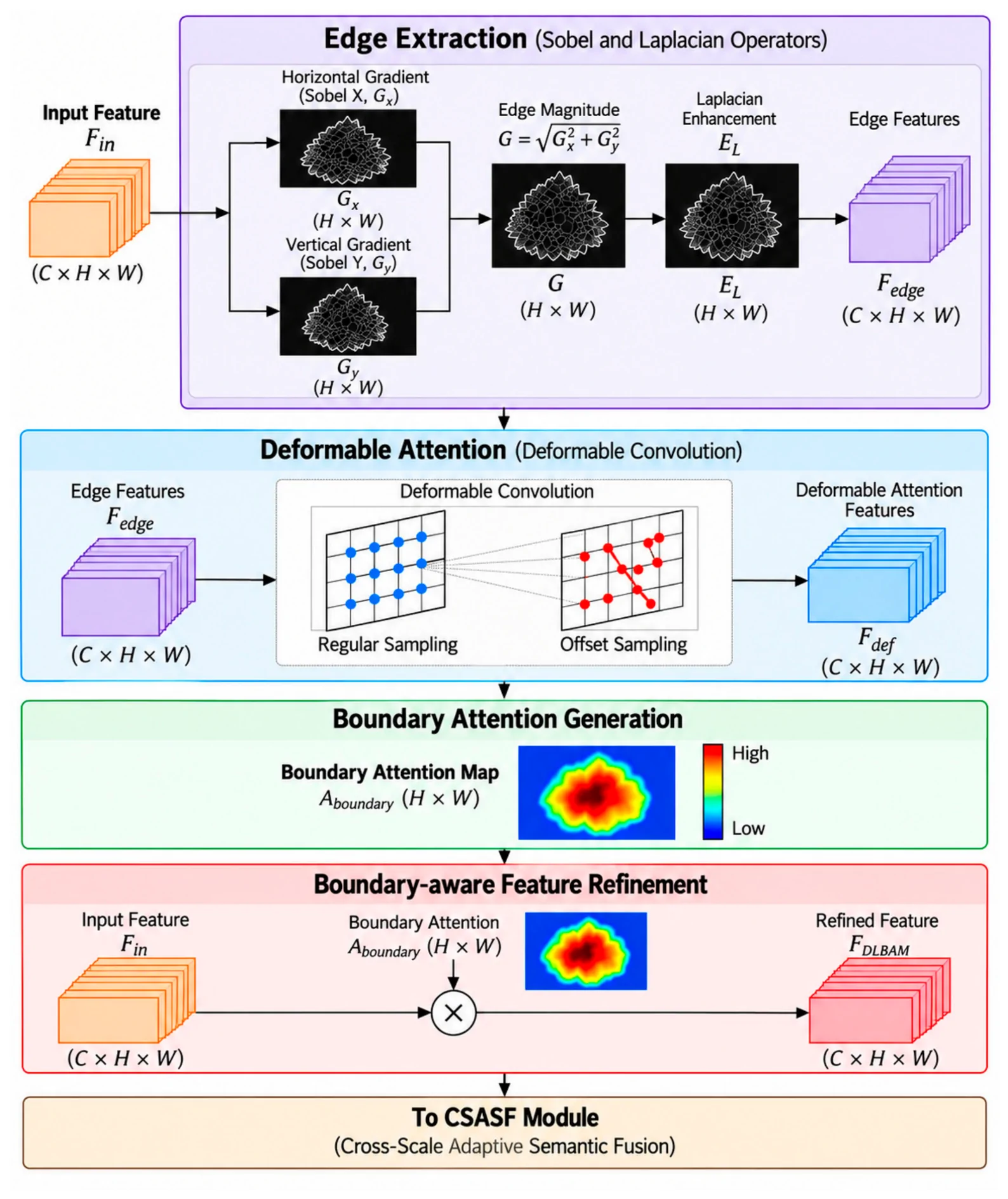
DLBAM for boundary-aware feature refinement.

**Figure 8:**
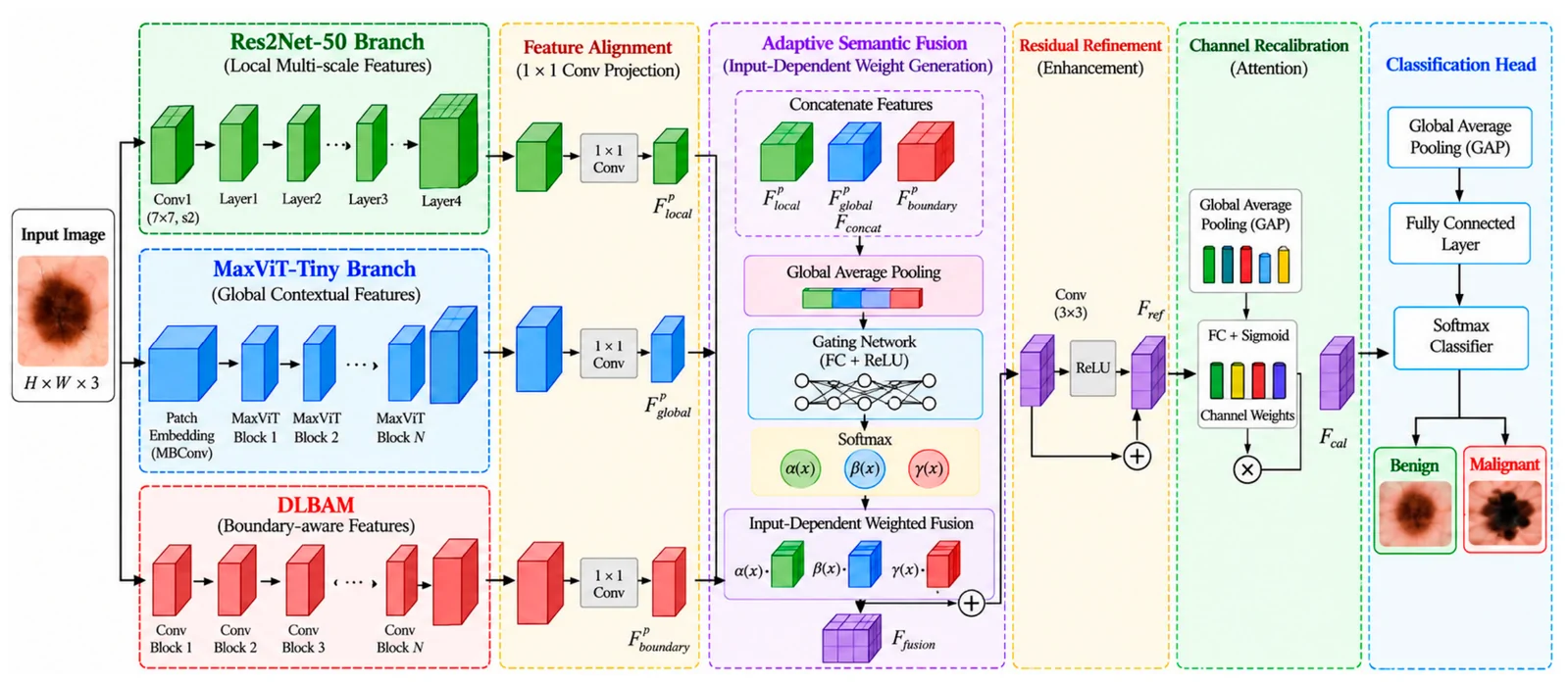
CSASF-based feature fusion and melanoma classification framework.

**Figure 9:**
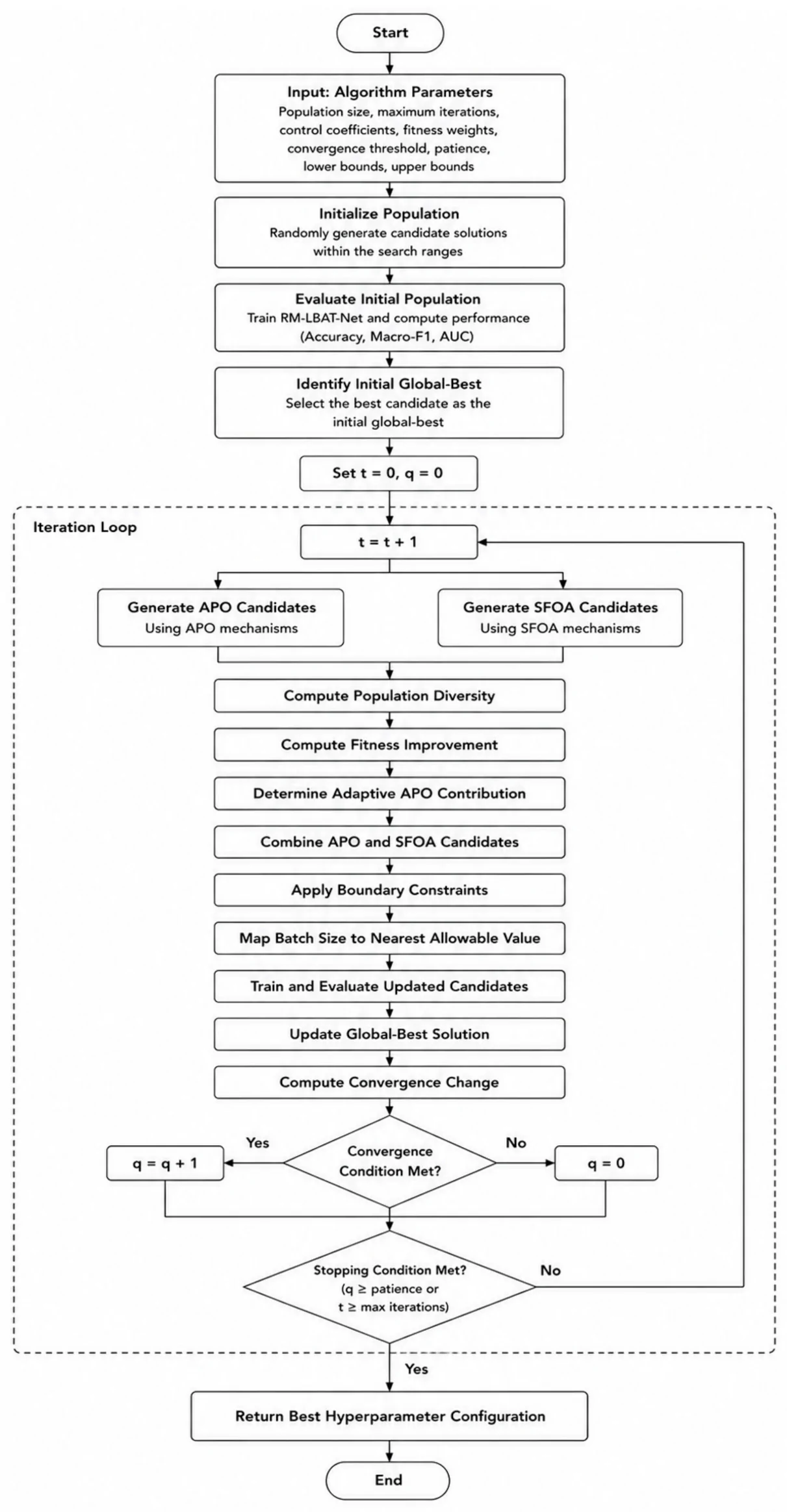
Overall flowchart of the proposed AC-APSO-based hyperparameter optimization framework for RM-LBAT-Net.

**Figure 10:**
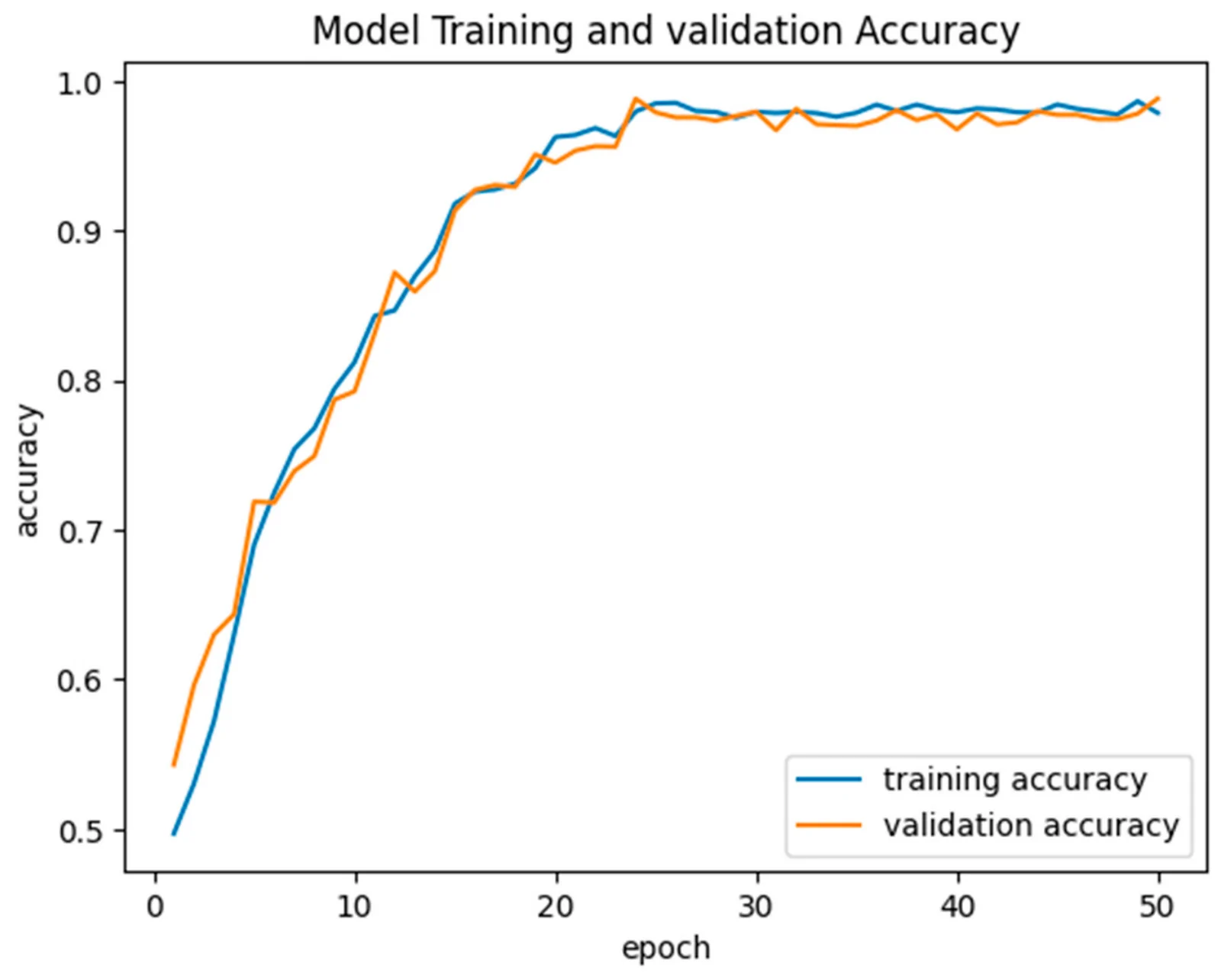
Training and validation accuracy profiles.

**Figure 11:**
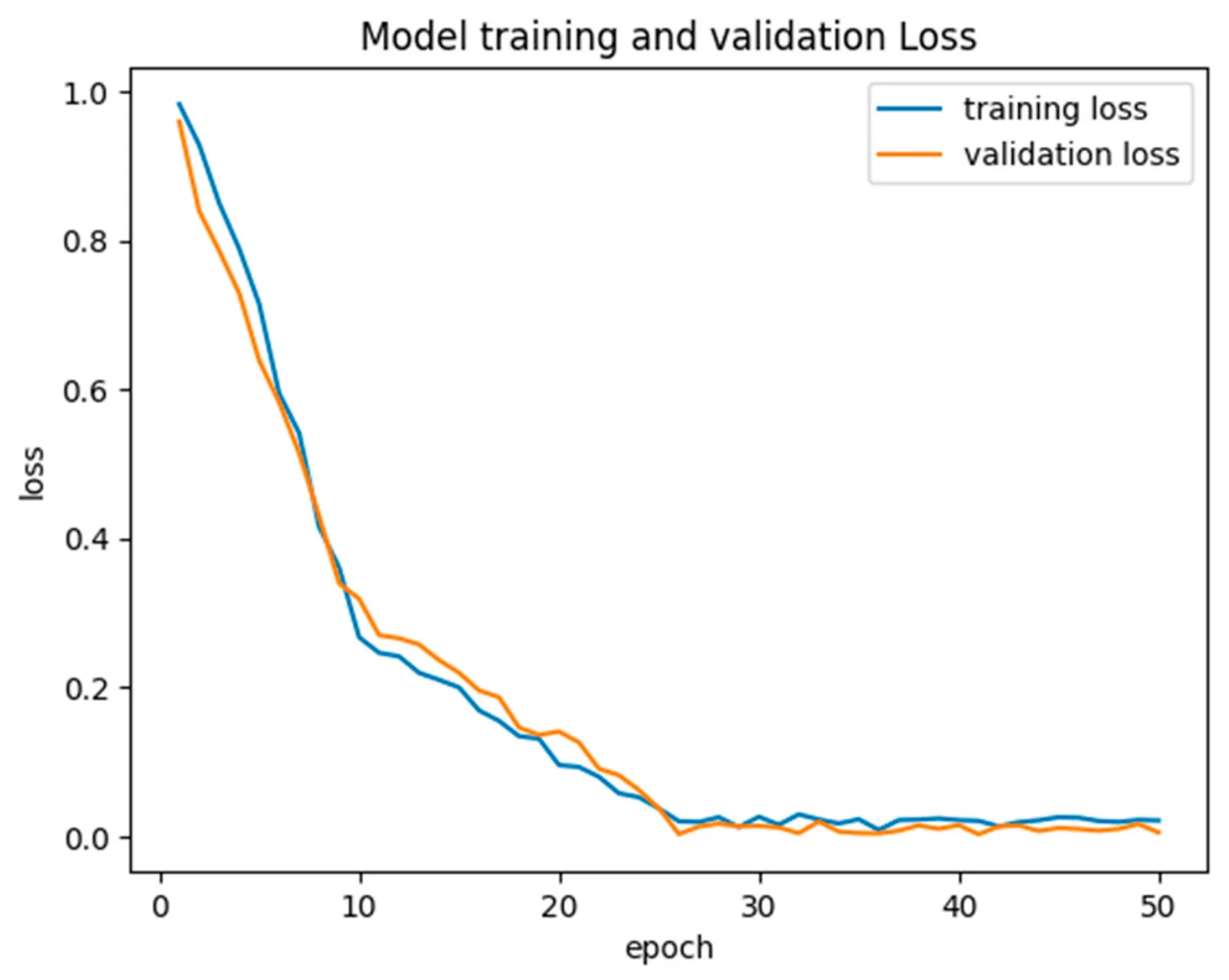
Training and validation loss profiles.

**Figure 12:**
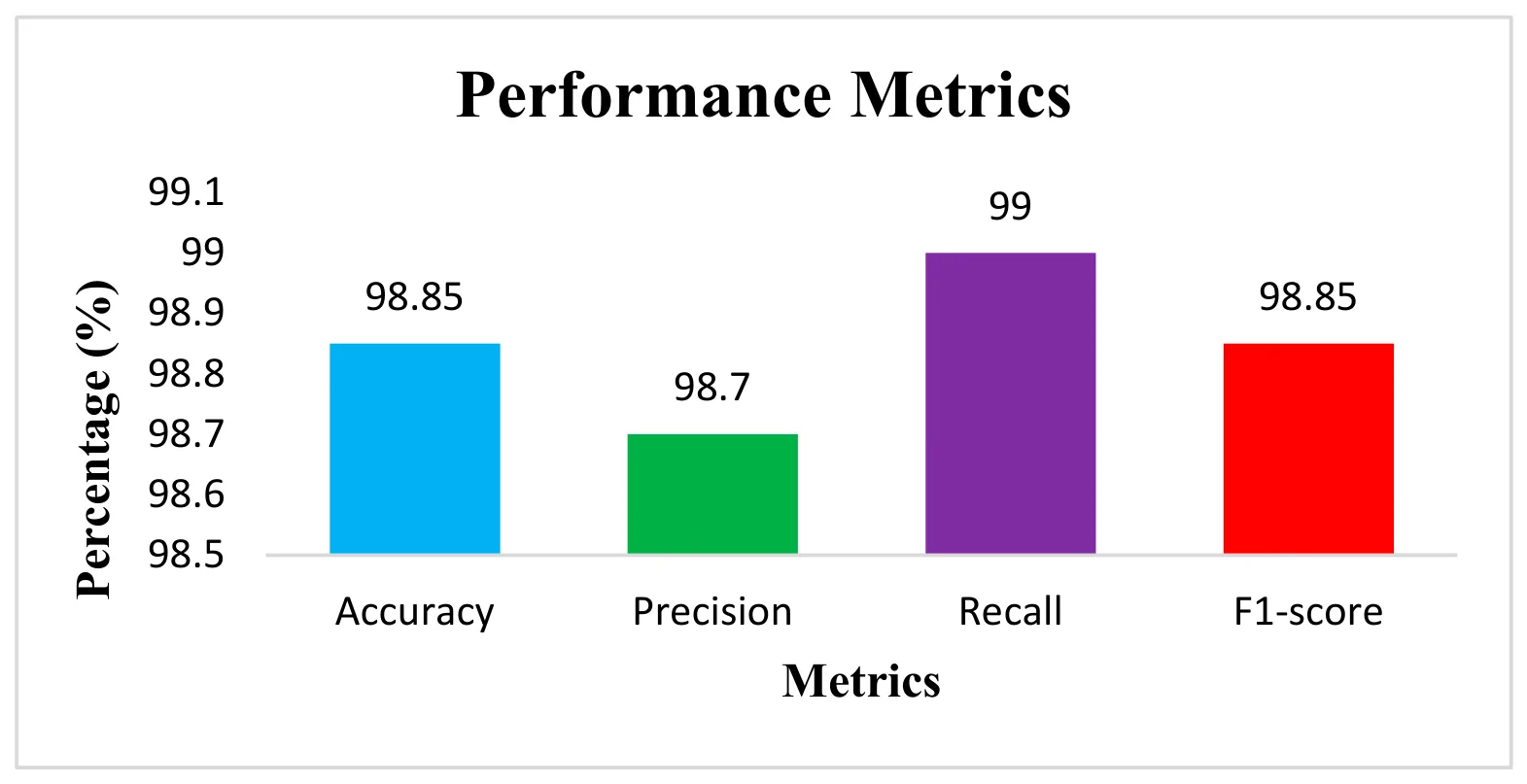
Overall performance metrics of the proposed AC-APSO-optimized RM-LBAT-Net framework.

**Figure 13:**
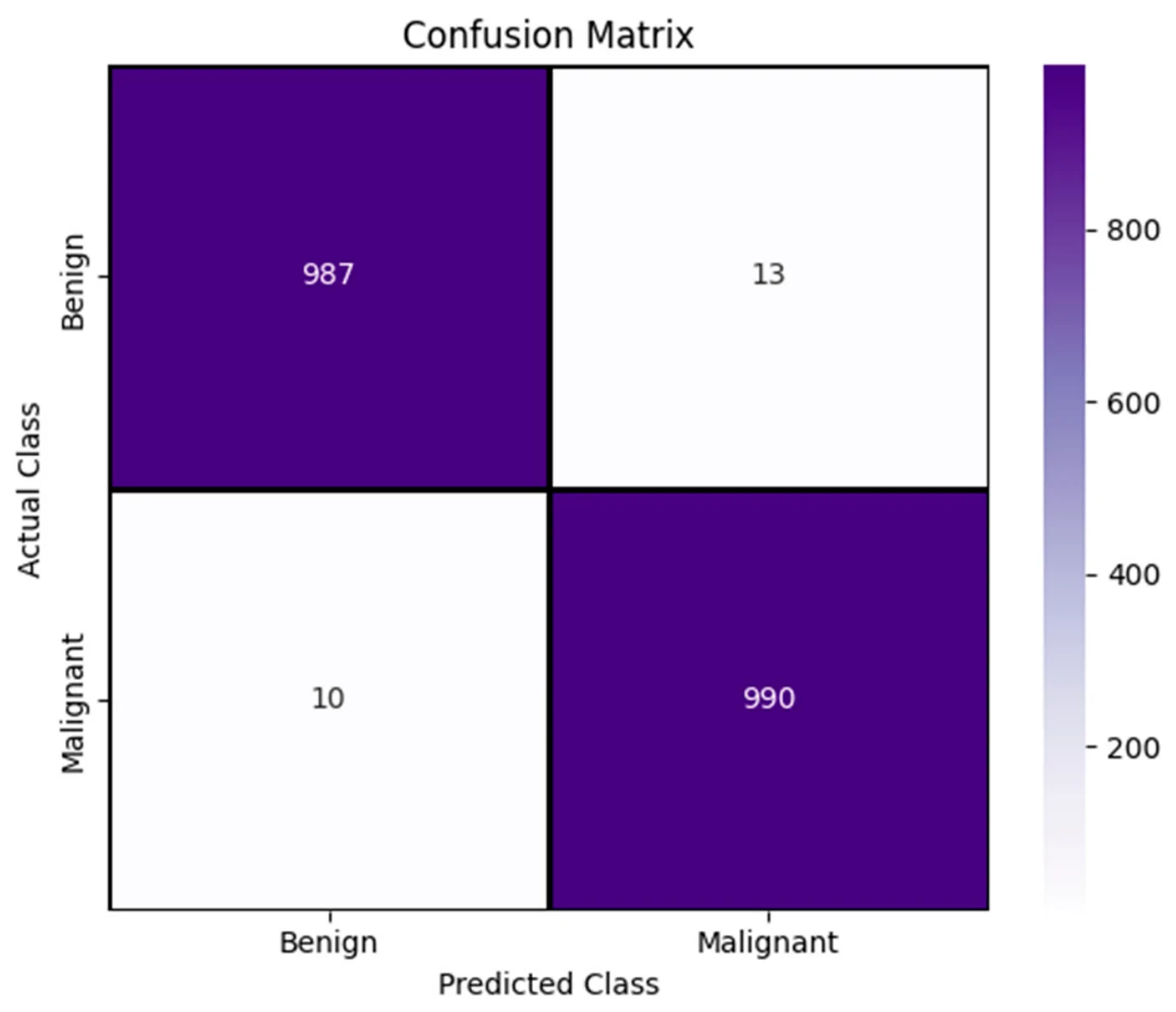
Confusion matrix.

**Figure 14:**
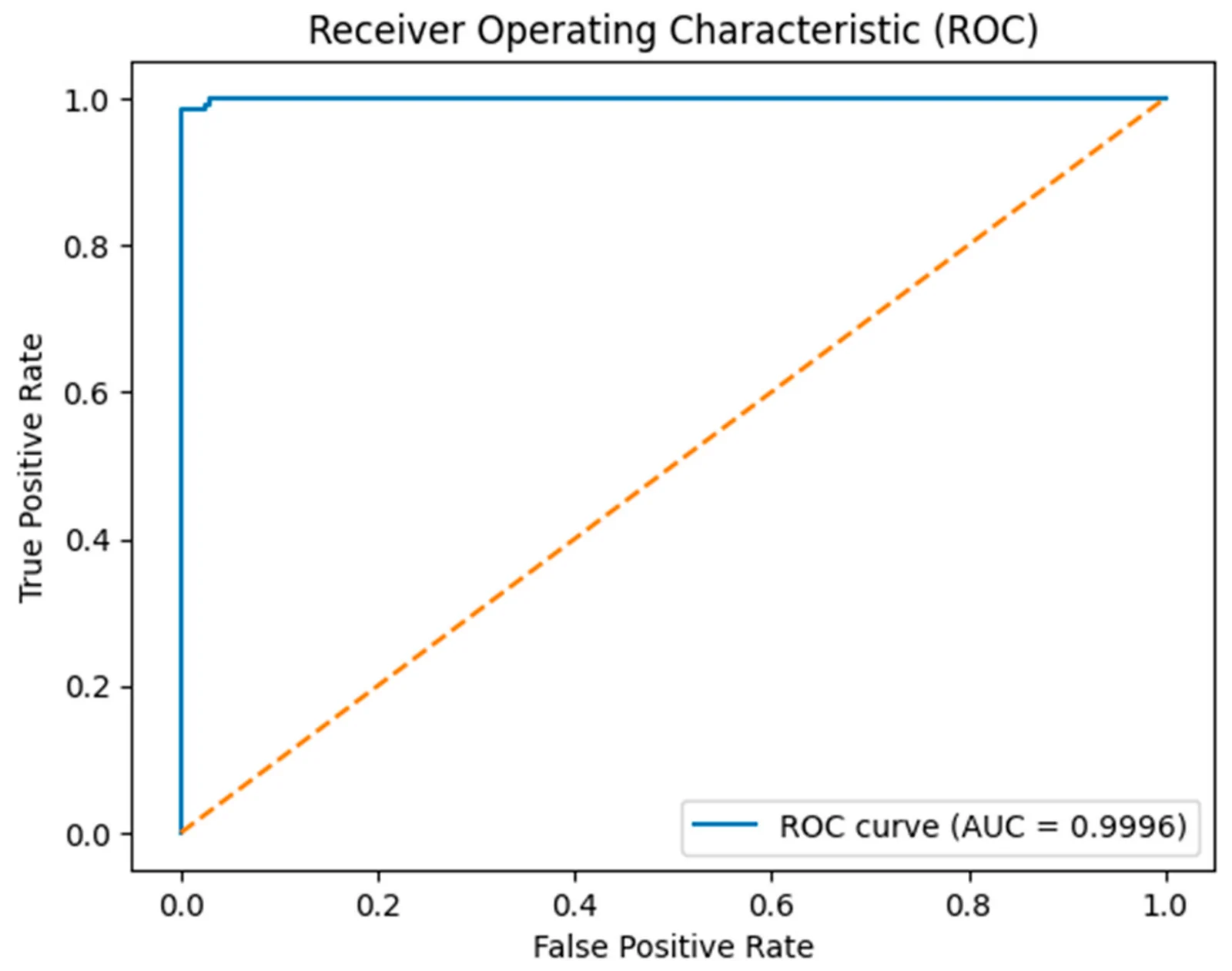
ROC curve.

**Figure 15:**
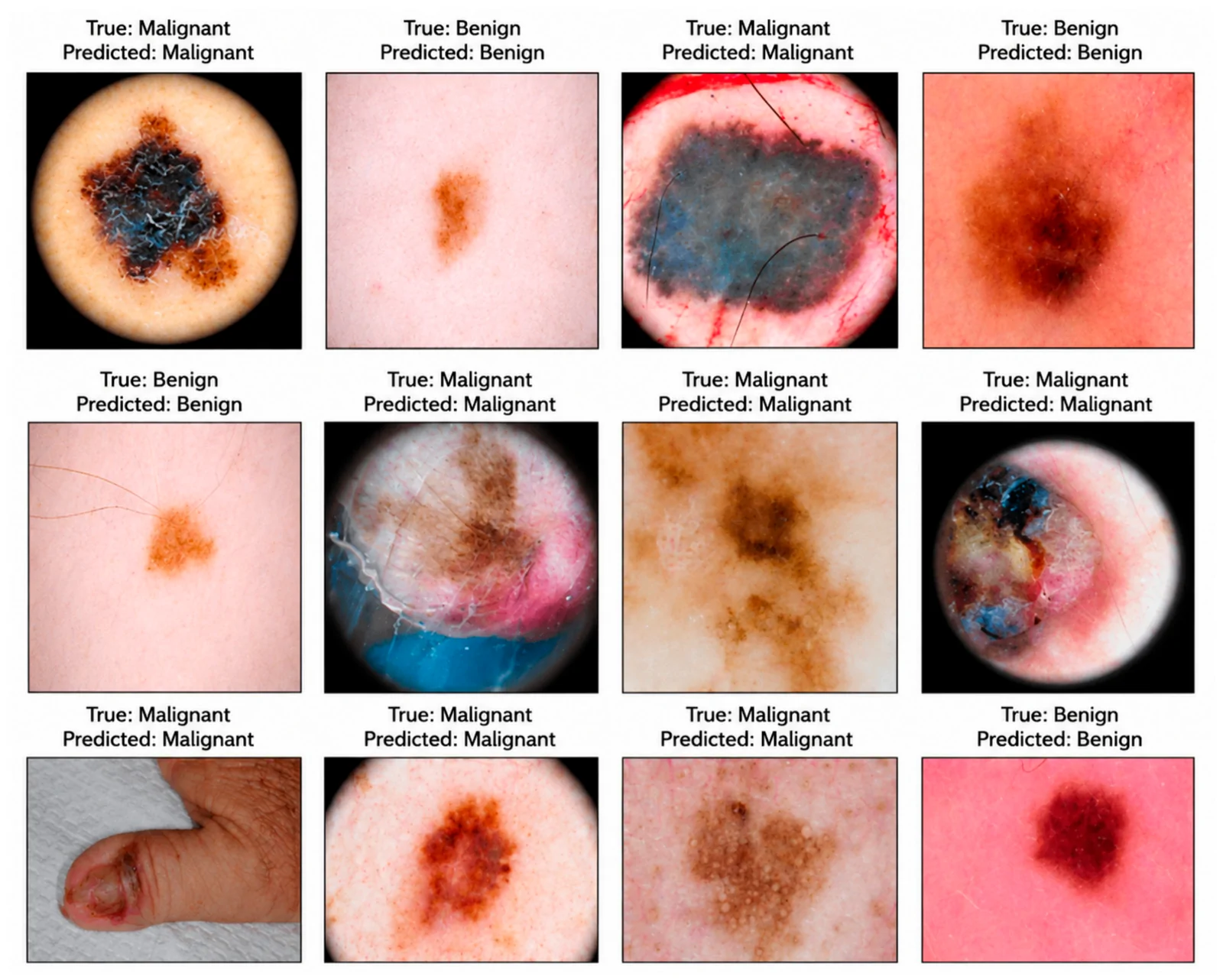
Prediction visualization of benign and malignant dermoscopic lesions.

**Figure 16:**
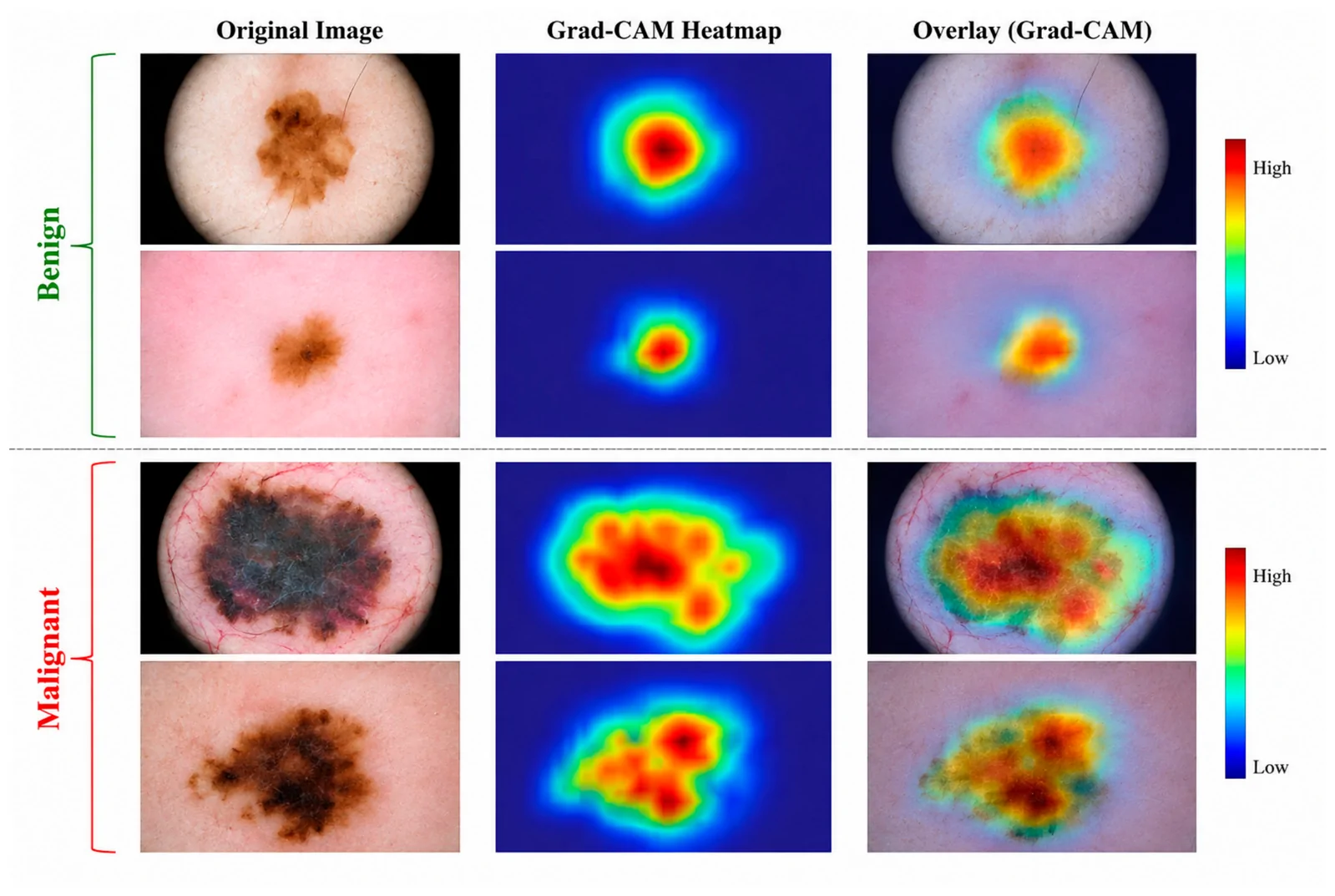
Grad-CAM visualization of lesion-sensitive regions in dermoscopic images.

**Figure 17:**
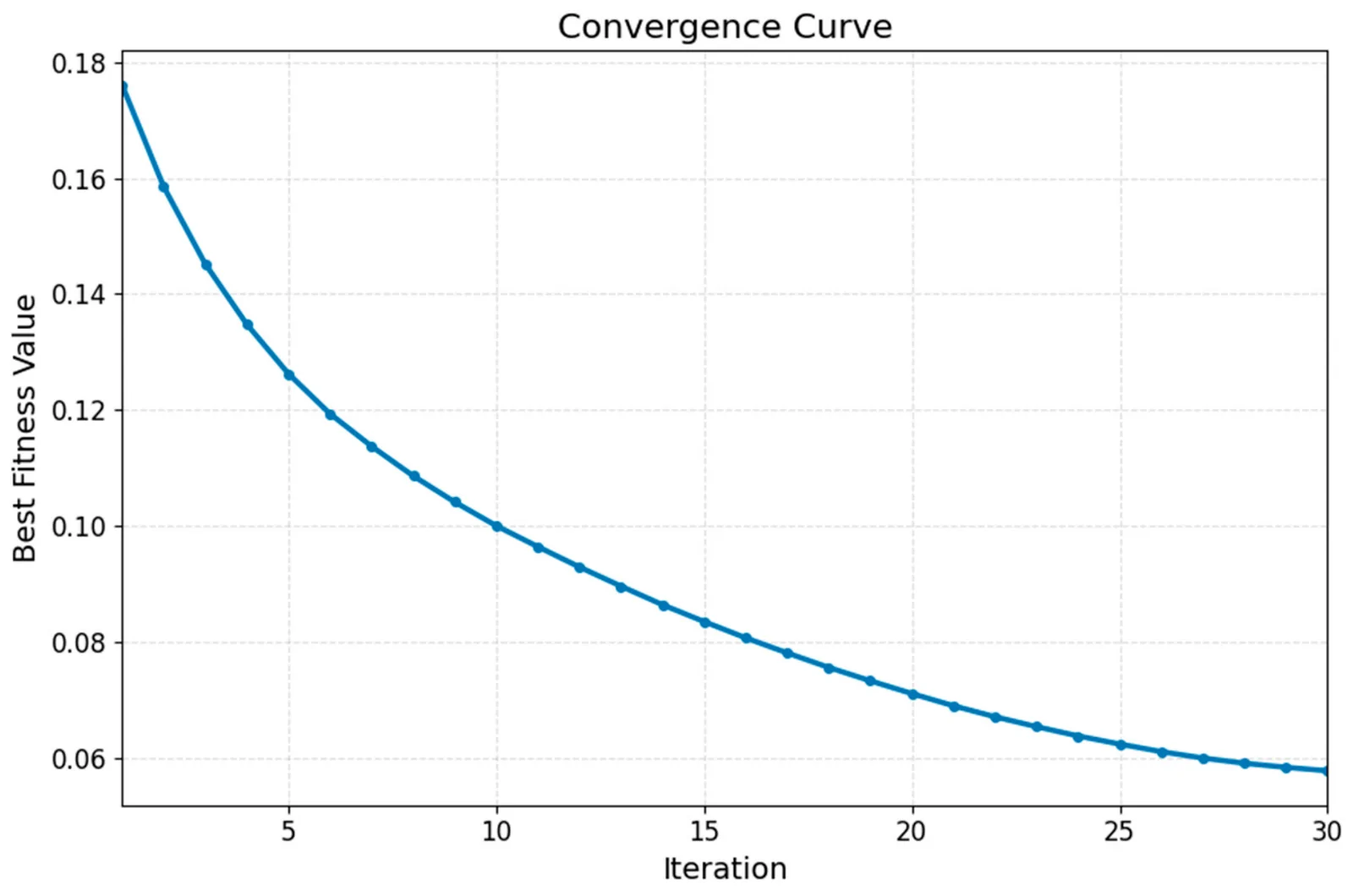
AC-APSO convergence curve.

**Figure 18:**
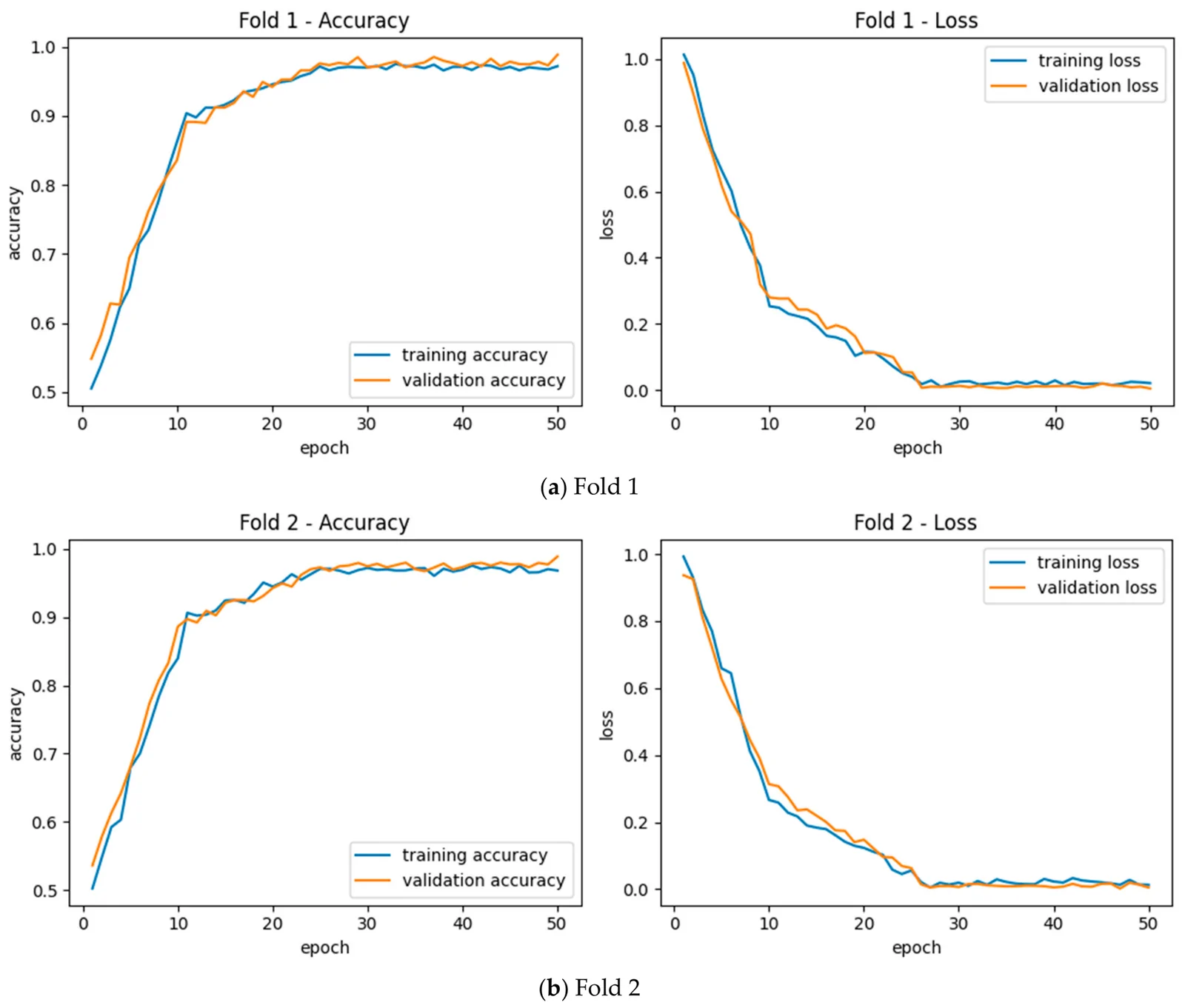

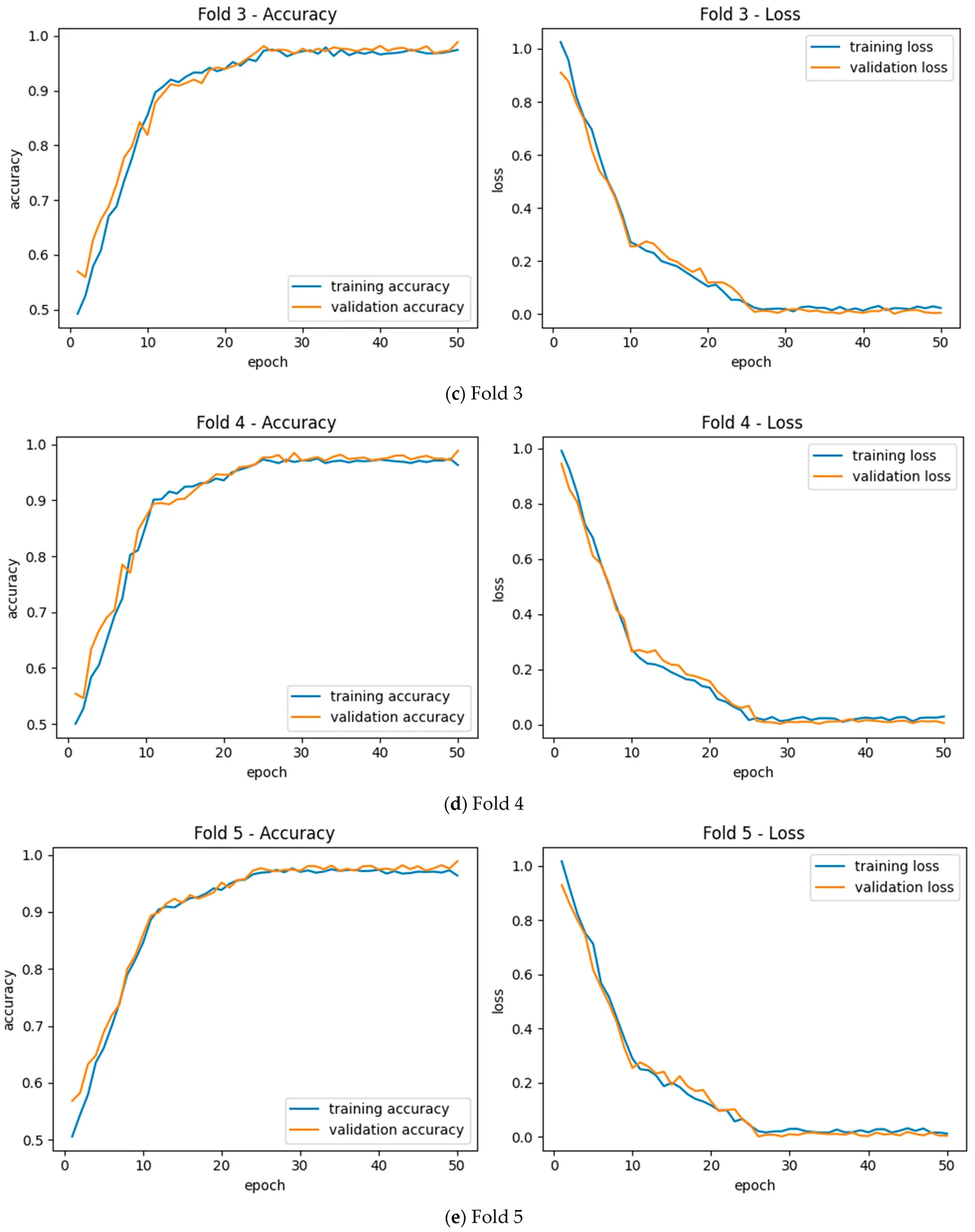
Accuracy and loss curves for training and validation across the 5-fold cross-validation.

**Figure 19:**
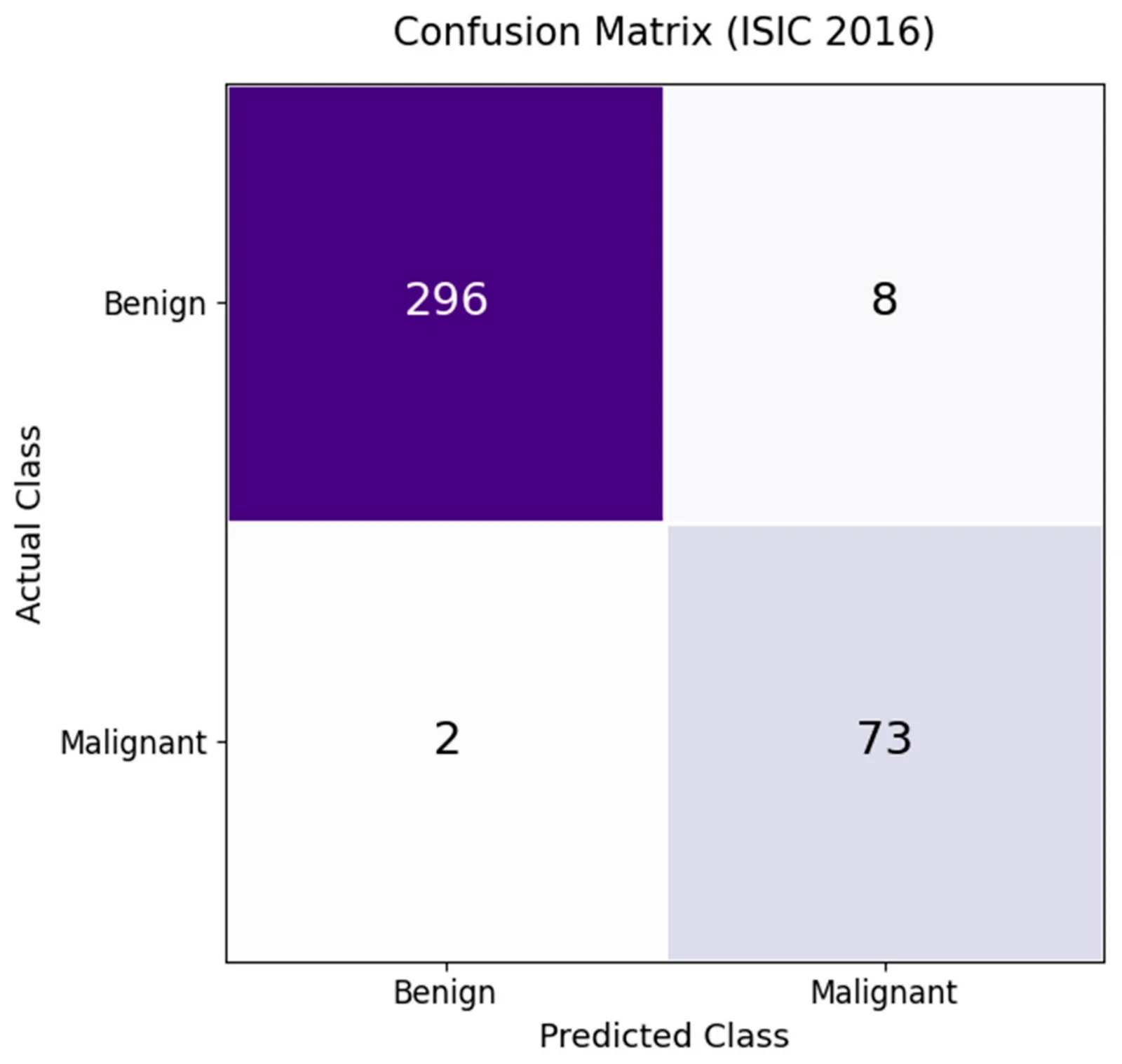
Confusion matrix of the AC-APSO-optimized RM-LBAT-Net on the ISIC 2016 external evaluation dataset.

**Table 1:** Search ranges of hyperparameters optimized using AC-APSO.

Hyperparameter	Search Range	Parameter Type
Learning Rate	1 × 10^−5^–1 × 10^−3^	Continuous
Batch Size	{16, 32, 64}	Discrete
Dropout Rate	0.10–0.50	Continuous
Weight Decay	1 × 10^−6^–1 × 10^−4^	Continuous
Focal Loss Weight	0.50–0.90	Continuous

**Table 2:** Fixed training and architectural configuration of RM-LBAT-Net.

Parameter	Configuration
Optimizer	Adam
Epochs (final training)	50
Res2Net-50 Variant	26w4s
Res2Net-50 Base Width	26
Res2Net-50 Scale Factor	4
Res2Net-50 Stage Depths	[3, 4, 6, 3]
MaxViT-Tiny Attention Heads	8
MaxViT-Tiny Window Size	7 × 7
Activation Function	ReLU
Output Activation	Softmax

**Table 3:** AC-APSO optimization settings.

Parameter	Setting
Population size (*N*)	20
Maximum iterations (*T*)	30
Training epochs per candidate evaluation	10
Fitness criteria and weights	Validation accuracy, macro-F1, AUC; 1/3 each
Adaptive APO coefficients	*c*_1_ = 0.4, *c*_2_ = 0.4, *c*_3_ = 0.2
APO neighbor pairs (*n_p_*)	2
APO maximum proportion fraction (*p_f_*_,max_)	0.10
SFOA switching parameter (*G_p_*)	0.50
Convergence threshold (*δ*)	10^−4^
Early-stopping patience (*P*)	5 iterations
Stopping criterion	30 iterations reached, or Δ*F_t_* < 10^−4^ for 5 consecutive iterations
Random seed	42

**Table 4:** AC-APSO-derived optimal values for training hyperparameters.

Hyperparameter	Optimized Value
Learning Rate	0.00032
Batch Size	32
Dropout Rate	0.25
Weight Decay	0.00001
Focal Loss Weight *α*^∗^	0.70

**Table 5:** Classification performance of the AC-APSO-optimized RM-LBAT-Net framework.

Class	Precision	Recall	F1-Score
Benign	0.9900	0.9870	0.9885
Malignant	0.9870	0.9900	0.9885
Accuracy	0.9885		
Macro Avg	0.9885	0.9885	0.9885
Weighted Avg	0.9885	0.9885	0.9885

**Table 6:** Component-wise ablation results under fixed and AC-APSO-selected training configurations.

Model	Architecture	Configuration	TN	FP	FN	TP	Accuracy (%)	Precision (%)	Recall (%)	F1-Score (%)
M1	Res2Net-50	Fixed	902	98	100	900	90.10	90.18	90.00	90.09
M2	+MaxViT-T	Fixed	952	48	50	950	95.10	95.19	95.00	95.10
M3	+DLBAM	Fixed	972	28	30	970	97.10	97.19	97.00	97.10
M4	+CSASF (complete)	Fixed	975	25	30	970	97.25	97.49	97.00	97.24
A1	Res2Net-50	AC-APSO	934	66	69	931	93.25	93.38	93.10	93.24
A2	+MaxViT-T	AC-APSO	965	35	38	962	96.35	96.49	96.20	96.34
A3	+DLBAM	AC-APSO	982	18	21	979	98.05	98.19	97.90	98.05
A4	+CSASF (complete)	AC-APSO	987	13	10	990	98.85	98.70	99.00	98.85

Note: Each “+” denotes the addition of the component to the preceding configuration. TN, FP, FN, and TP are computed on the 2000-image test subset with malignant as the positive class.

**Table 7:** Optimization-wise ablation analysis of the proposed AC-APSO strategy.

Config.	Optimization Strategy	Best Val. Fitness ↓	TN	FP	FN	TP	Acc. (%)	Prec. (%)	Rec. (%)	F1 (%)	Best Iter.	Time (h)
O1	No optimization (fixed)	0.112	975	25	30	970	97.25	97.49	97.00	97.24	—	—
O2	APO	0.094	977	23	27	973	97.50	97.69	97.30	97.50	24	42.6
O3	SFOA	0.082	979	21	24	976	97.75	97.89	97.60	97.75	26	44.1
O4	APO + SFOA	0.069	983	17	19	981	98.20	98.30	98.10	98.20	28	47.3
O5	AC-APSO (Proposed)	0.058	987	13	10	990	98.85	98.70	99.00	98.85	30	51.6

Note: ↓ indicates that lower values are better. Optimization time denotes the total run time of each optimization run on an NVIDIA Tesla T4 GPU.

**Table 8:** Five-fold cross-validation results.

Fold	Accuracy (%)	Precision (%)	Recall (%)	F1-Score (%)	AUC
Fold 1	98.6	98.1	97.8	97.9	0.9992
Fold 2	98.7	98.3	97.9	98.1	0.9995
Fold 3	98.9	98.4	98.0	98.2	0.9997
Fold 4	98.8	98.2	97.9	98.0	0.9996
Fold 5	99.0	98.5	98.1	98.3	0.9998
Mean ± SD	98.80 ± 0.16	98.30 ± 0.16	97.94 ± 0.11	98.10 ± 0.16	0.9996 ± 0.0002

Note: SD: sample standard deviation computed across the five folds.

**Table 9:** Hyperparameter Sensitivity Analysis of AC-APSO-Optimized RM-LBAT-Net.

Hyperparameter	Value	Validation Accuracy (%)	Validation Macro-F1 (%)
Learning Rate	0.00010	96.91	96.89
	0.00020	97.81	97.80
	**0.00032**	**98.82**	**98.81**
	0.00050	98.26	98.25
	0.00100	97.42	97.40
Batch Size	16	98.15	98.14
	**32**	**98.82**	**98.81**
	64	98.32	98.31
Dropout Rate	0.10	98.26	98.25
	0.20	98.54	98.53
	**0.25**	**98.82**	**98.81**
	0.30	98.48	98.48
	0.50	97.64	97.63
Weight Decay	1 × 10^−6^	98.32	98.31
	5 × 10^−6^	98.54	98.53
	**1 × 10^−5^**	**98.82**	**98.81**
	5 × 10^−5^	98.37	98.36
	1 × 10^−4^	97.92	97.91
Focal Loss Weight (α)	0.50	98.09	98.08
	0.60	98.43	98.42
	**0.70**	**98.82**	**98.81**
	0.80	98.48	98.48
	0.90	98.03	98.02

Bold values indicate the optimal hyperparameter settings identified by the AC-APSO.

**Table 10:** External validation performance on the ISIC 2016 lesion-classification dataset.

Metric	Value (%)	95% CI (%)
Accuracy	97.36	95.21–98.56
Precision	90.12	81.70–94.91
Recall	97.33	90.79–99.27
F1-score	93.59	89.19–97.18

**Table 11:** Head-to-head comparison with baseline and contemporary models.

Model	TN	FP	FN	TP	Accuracy (%)	95% CI (%)	Precision (%)	Recall (%)	F1-Score (%)
CNN (Custom)	881	119	124	876	87.85	86.35–89.21	88.04	87.60	87.82
ResNet50	894	106	110	890	89.20	87.76–90.49	89.36	89.00	89.18
EfficientNet-B0	950	50	54	946	94.80	93.74–95.69	94.98	94.60	94.79
ViT	959	41	46	954	95.65	94.67–96.46	95.88	95.40	95.64
ConvNeXtV2-T	966	34	38	962	96.40	95.49–97.13	96.59	96.20	96.39
SwinV2-T	973	27	32	968	97.05	96.21–97.71	97.29	96.80	97.04
RM-LBAT-Net (M4, fixed)	975	25	30	970	97.25	96.44–97.88	97.49	97.00	97.24
Proposed (AC-APSO)	987	13	10	990	98.85	98.28–99.23	98.70	99.00	98.85

**Table 12:** Comparative analysis with state-of-the-art melanoma detection approaches.

Ref	Year	Methodology	Accuracy (%)	Limitations
Alrabai et al. [13]	2026	DenseNet201	87.88	Dataset homogeneity; no computational efficiency analysis
Hermosilla et al. [30]	2026	Metaheuristic-optimized CNN (CS, FA, WOA, PSO, GWO, CSA)	91.25	Shallow CNN backbone; single-dataset evaluation
Aksoy et al. [28]	2024	ConvNeXt Base	91.50	No explainability or uncertainty analysis; image acquisition conditions not considered
Nguyen et al. [14]	2025	EfficientNet	92.50	Sensitivity to image variability; no clinical metadata integration
Mittal et al. [16]	2024	Deep Spiking CNN	95.07	Reliance on handcrafted feature engineering; limited interpretability
Nasiri et al. [31]	2026	GWO–PSO-optimized CNN	95.30	No external validation reported
Mahmud et al. [15]	2025	Xception	96.48	Limited dataset diversity; computational complexity
Chiu et al. [24]	2025	EfficientNet + ViT + Swin Transformer	96.90	High computational complexity; limited generalizability
Mateen et al. [29]	2024	U-Net + Inception-ResNet-v2 + ViT	98.65	Requires a separate segmentation stage; multi-stage pipeline with high computational cost
Sajid et al. [32]	2026	Morphology-guided attention network with explainability and uncertainty estimation (7-class)	99.12	Evaluation limited to HAM10000; further validation on larger and more diverse datasets required
Proposed Model		RM-LBAT-Net	98.85	

Note: Accuracy values of the compared methods are taken from the original publications and were obtained using different datasets, data partitions, preprocessing procedures, and evaluation protocols. These values are therefore not directly comparable and do not constitute a controlled head-to-head comparison.

## Data Availability

The primary dermoscopic image dataset used in this study is publicly available on Kaggle: Melanoma Cancer Dataset (https://www.kaggle.com/datasets/bhaveshmittal/melanoma-cancer-dataset) (accessed on 18 February 2026). The dataset used for external validation, ISIC 2016 Challenge—Task 3: Lesion Classification, is publicly available through the International Skin Imaging Collaboration (ISIC) Archive (accessed on 6 March 2026).

## Supplementary Materials

The following supporting information can be downloaded at: https://www.mdpi.com/article/10.3390/biomimetics11100701/s1, Figure S1: Confusion matrices of the component-wise ablation configurations under the fixed training configuration: (a) M1 (Res2Net-50), (b) M2 (Res2Net-50 + MaxViT-T), (c) M3 (Res2Net-50 + MaxViT-T + DLBAM), and (d) M4 (Complete RM-LBAT-Net); Figure S2: Confusion matrices of the component-wise ablation configurations under the fixed training configuration: (a) M1 (Res2Net-50), (b) M2 (Res2Net-50 + MaxViT-T), (c) M3 (Res2Net-50 + MaxViT-T + DLBAM), and (d) M4 (Complete RM-LBAT-Net); Figure S3: Confusion matrices of the optimization-wise ablation configurations: (a) O1 (No optimization), (b) O2 (APO), (c) O3 (SFOA), (d) O4 (APO + SFOA), and (e) O5 (AC-APSO).
