# Supplementary material for: A Dual-Stream Feature Learning Framework with Adaptive Bio-Inspired Protozoa–Starfish Optimization for Melanoma Classification
Source: Biomimetics (Basel). 2026 Oct 2;11(10):701. doi: 10.3390/biomimetics11100701 (PMC13702299; doi:10.3390/biomimetics11100701)
Supplement: Supplementary file 1 [file biomimetics-11-00701-s001.zip › biomimetics-4585415-supplementary.pdf]

Supplementary Files

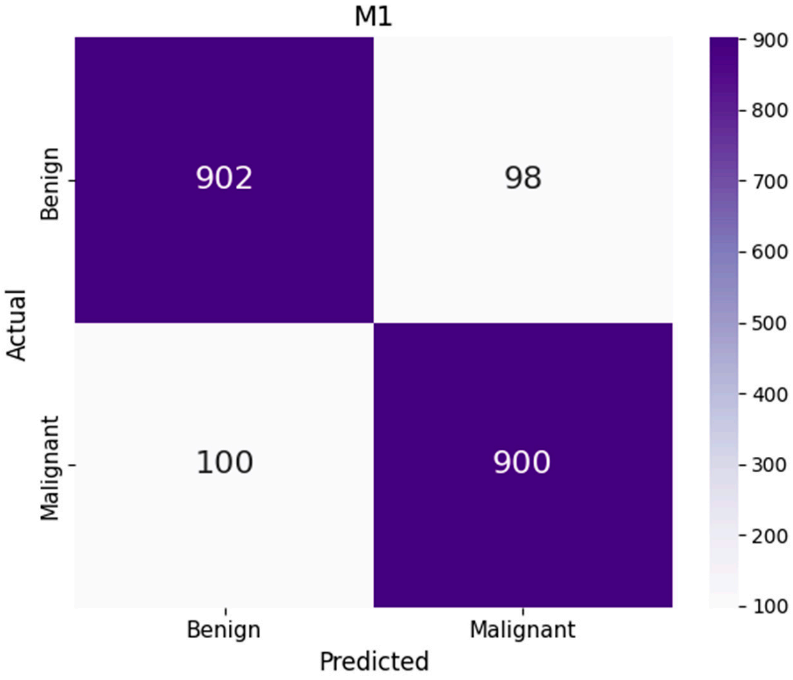

(a)

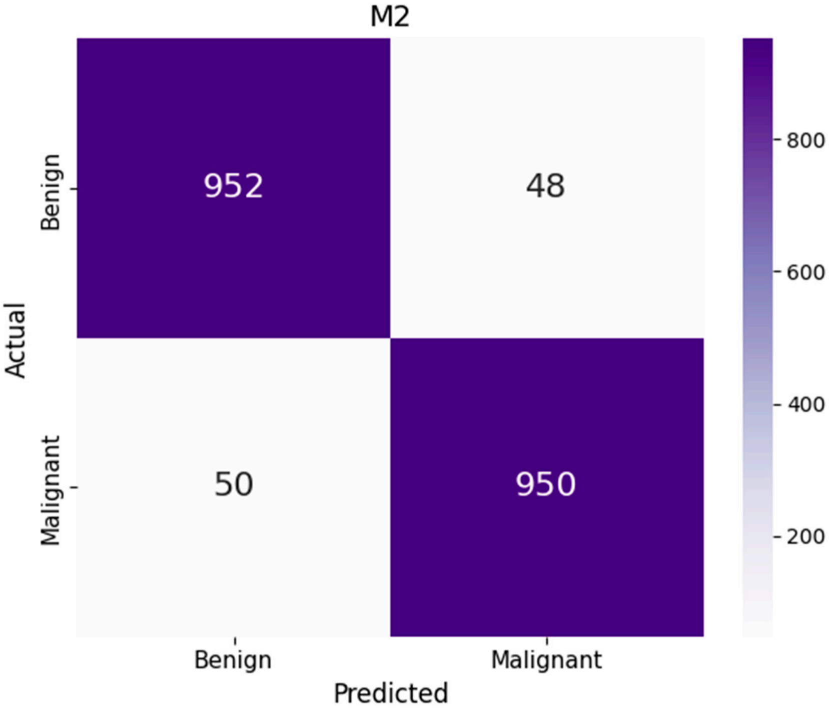

(b)

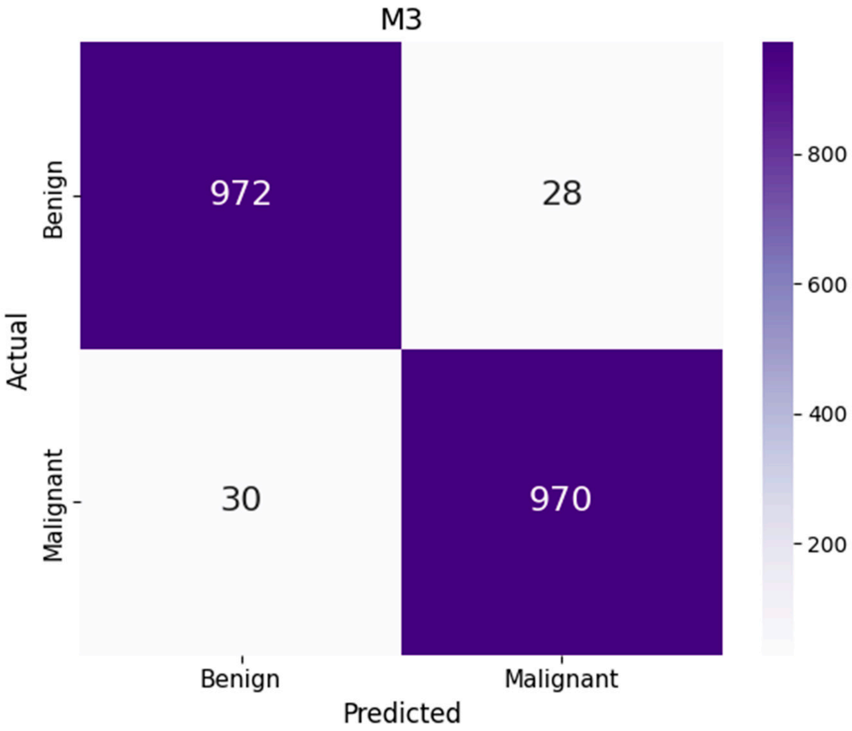

(c)

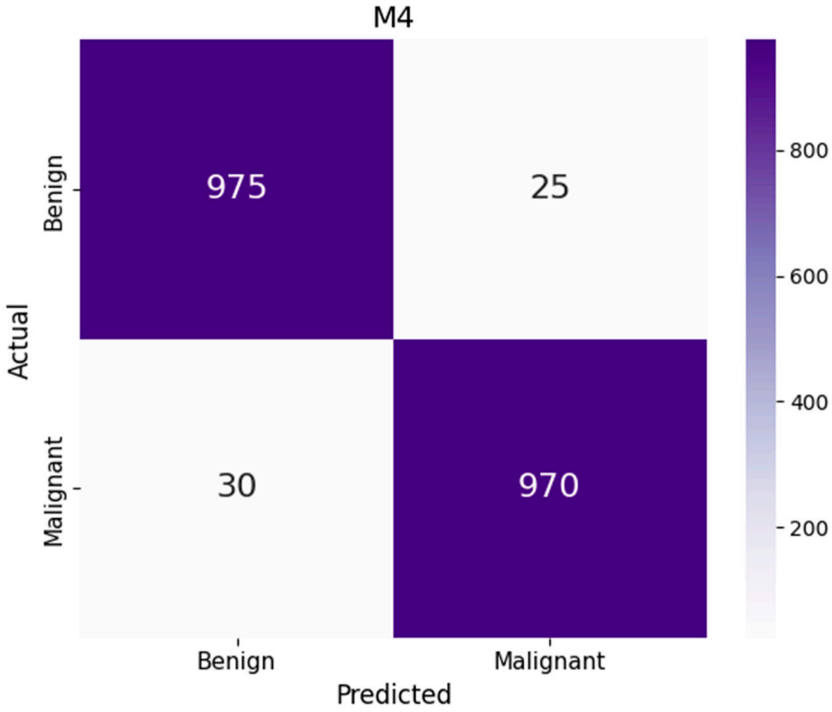

(d)

Figure S1: Confusion matrices of the component-wise ablation configurations under the fixed training configuration: (a) M1 (Res2Net-50), (b) M2 (Res2Net-50 + MaxViT-T), (c) M3 (Res2Net-50 + MaxViT-T + DLBAM), and (d) M4 (Complete RM-LBAT-Net).

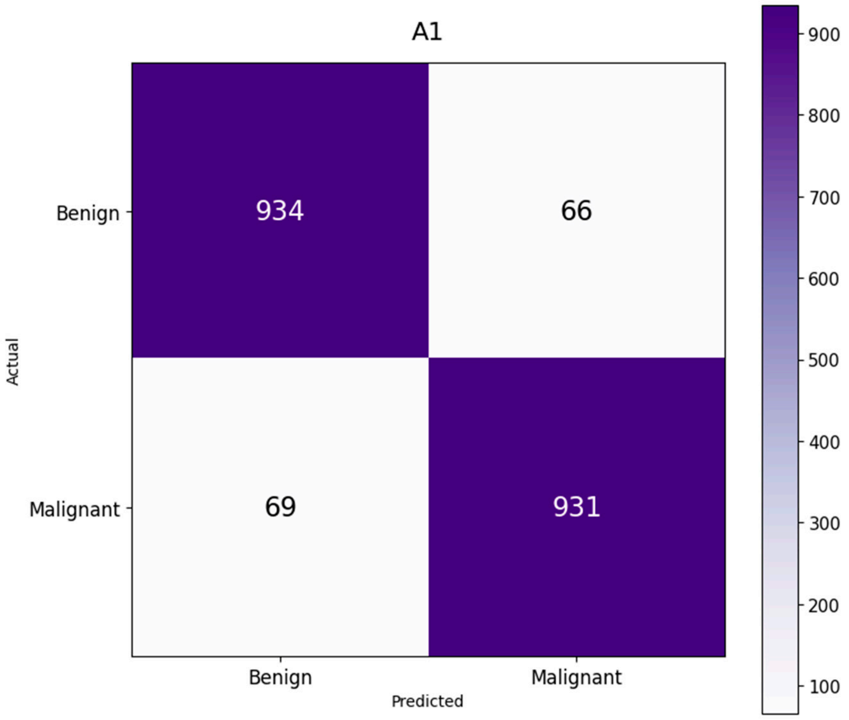
A1
Benign
Malignant
Actual
934
66
69
931
Benign
Malignant
Predicted
900
800
700
600
500
400
300
200
100

(a)

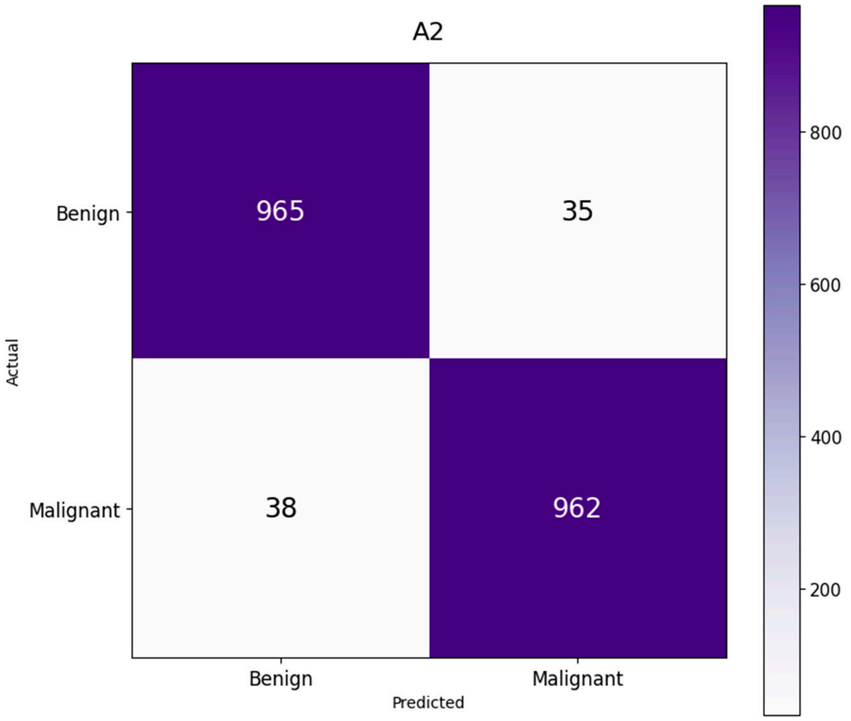
A2
Benign
Malignant
Actual
965
35
38
962
Benign
Malignant
Predicted
800
600
400
200

(b)

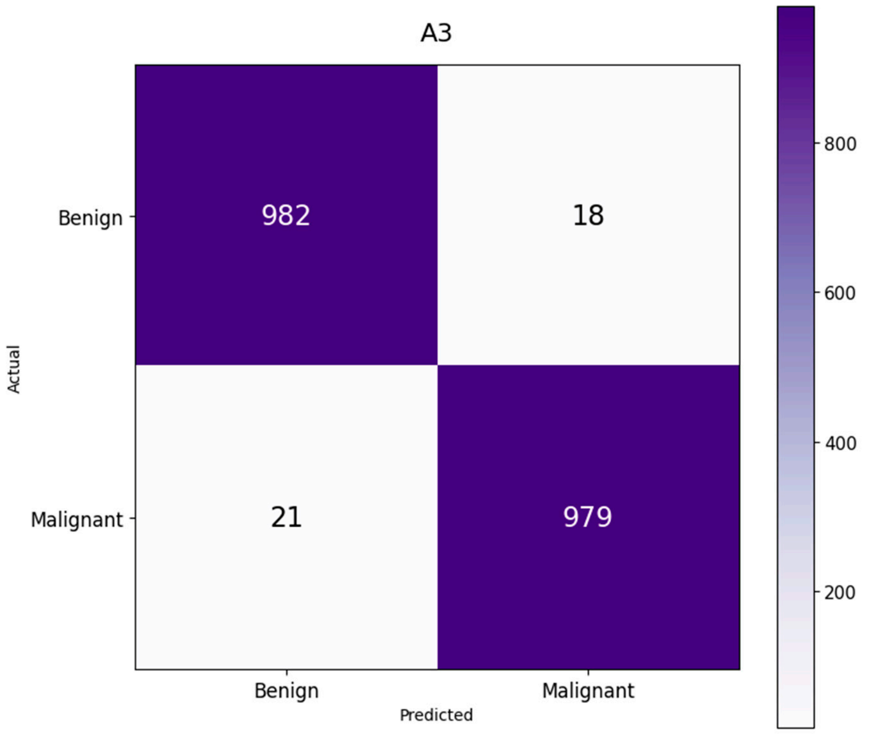

(c)

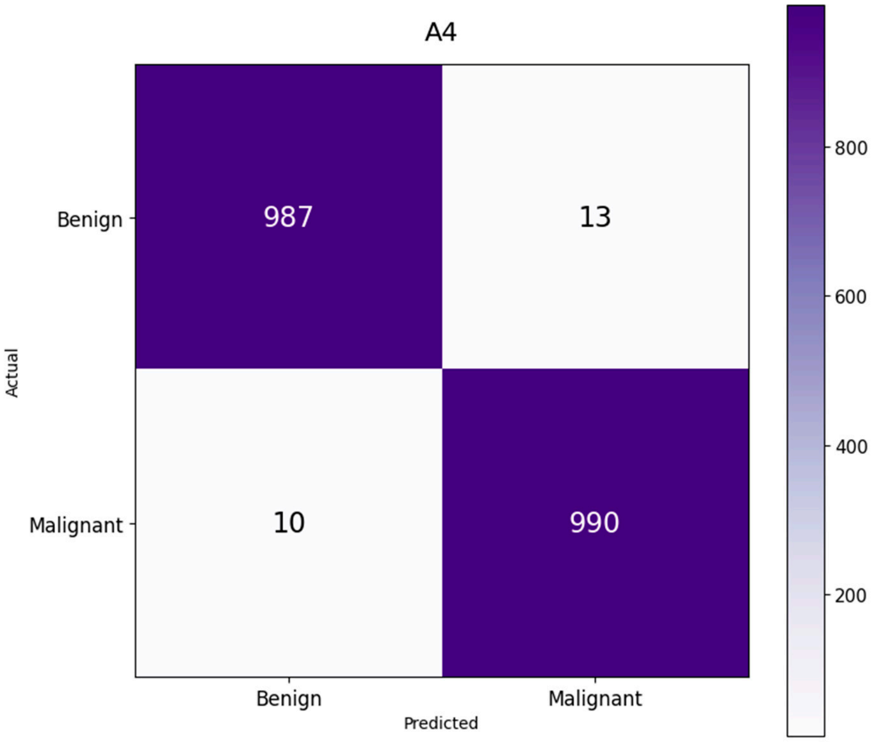

(d)

Figure S2: Confusion matrices of the component-wise ablation configurations under the AC-APSO-selected training configuration: (a) A1 (Res2Net-50+ AC-APSO), (b) A2 (Res2Net-50 + MaxViT-T+ AC-APSO), (c) A3 (Res2Net-50 +

MaxViT-T + DLBAM+ AC-APSO), and (d) A4 (Complete RM-LBAT-Net+ AC-APSO).

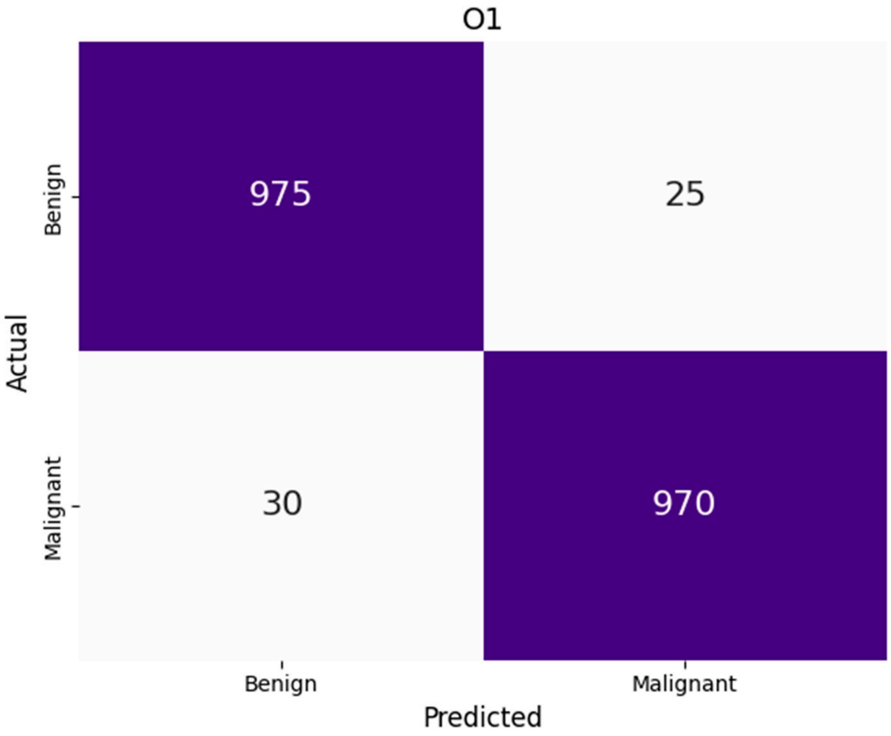

(a)

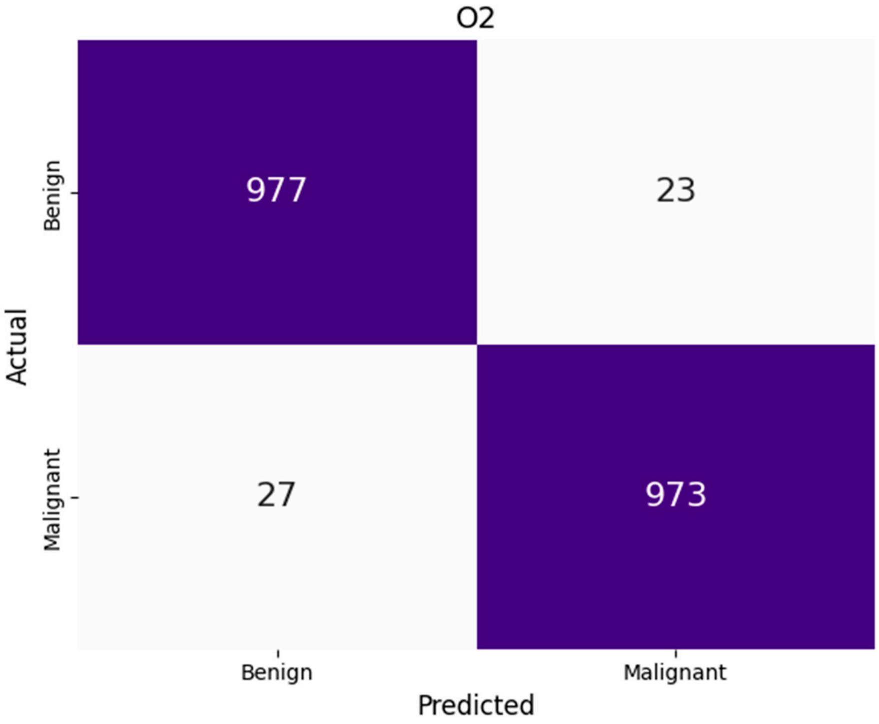

(b)

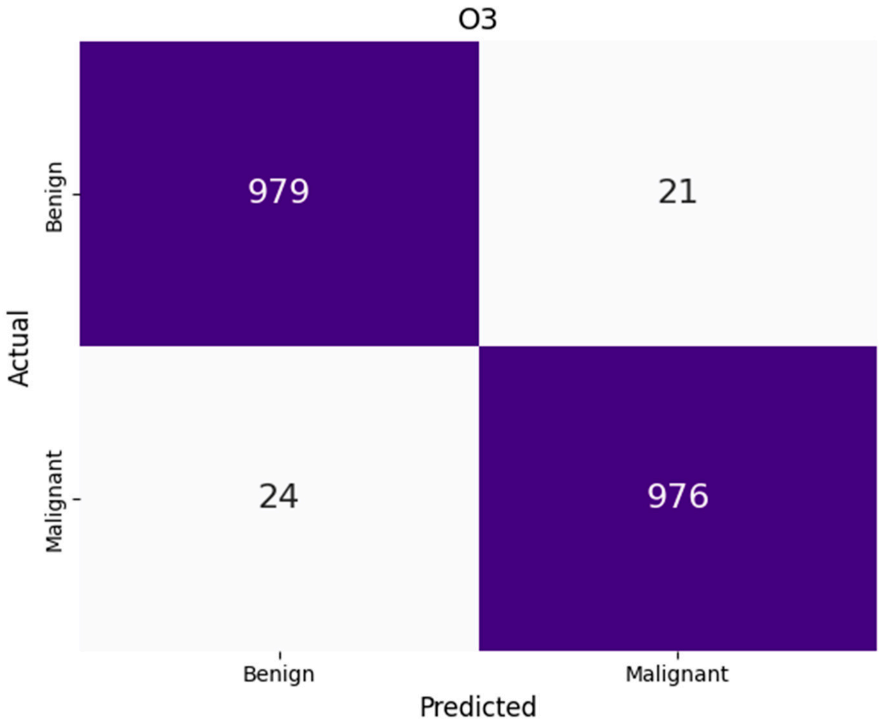
O3
979
21
24
976
Benign
Malignant
Actual
Benign
Malignant
Predicted

(c)

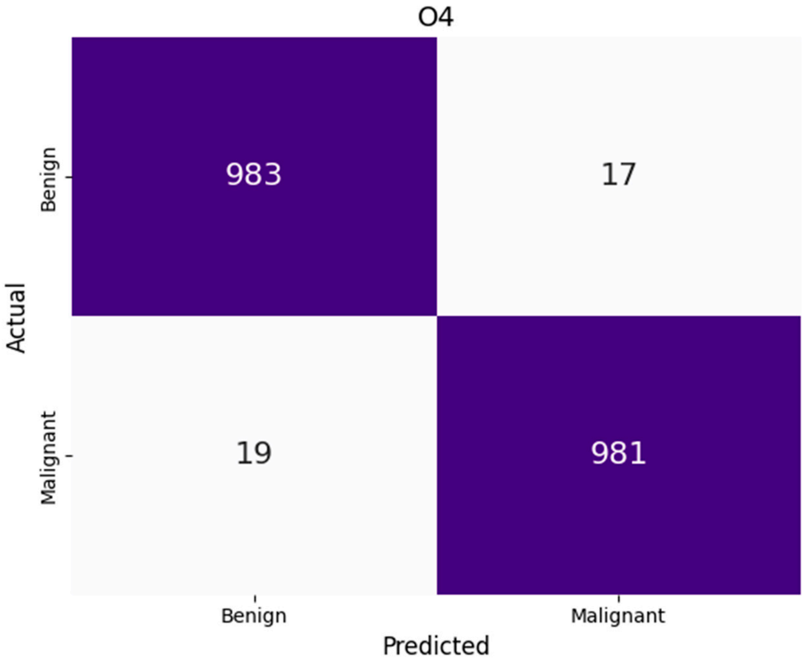
O4
983
17
19
981
Benign
Malignant
Actual
Benign
Malignant
Predicted

(d)

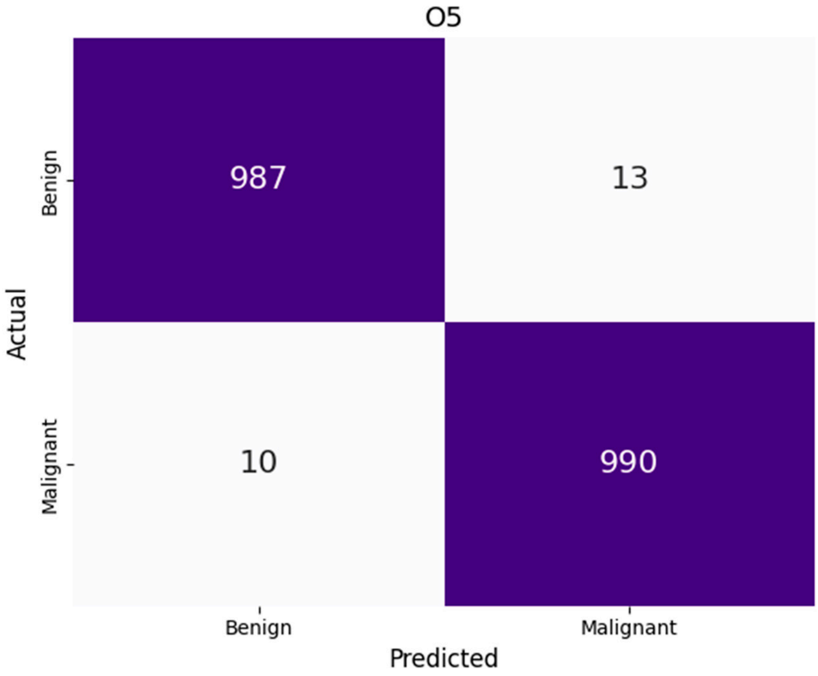

(e)

Figure S3: Confusion matrices of the optimization-wise ablation configurations: (a) O1 (No optimization), (b) O2 (APO), (c) O3 (SFOA), (d) O4 (APO + SFOA), and (e) O5 (AC-APSO).
